# Developing technologies to assess vascular ageing: a roadmap from VascAgeNet

**DOI:** 10.1088/1361-6579/ad548e

**Published:** 2024-12-30

**Authors:** Serena Zanelli, Davide Agnoletti, Jordi Alastruey, John Allen, Elisabetta Bianchini, Vasiliki Bikia, Pierre Boutouyrie, Rosa Maria Bruno, Rachel Climie, Djammaleddine Djeldjli, Eugenia Gkaliagkousi, Alessandro Giudici, Kristina Gopcevic, Andrea Grillo, Andrea Guala, Bernhard Hametner, Jayaraj Joseph, Parmis Karimpour, Vimarsha Kodithuwakku, Panicos A Kyriacou, Antonios Lazaridis, Mai Tone Lønnebakken, Maria Raffaella Martina, Christopher Clemens Mayer, P M Nabeel, Petras Navickas, János Nemcsik, Stefan Orter, Chloe Park, Telmo Pereira, Giacomo Pucci, Ana Belen Amado Rey, Paolo Salvi, Ana Carolina Gonçalves Seabra, Ute Seeland, Thomas van Sloten, Bart Spronck, Gerard Stansby, Indra Steens, Thomas Stieglitz, Isabella Tan, Dave Veerasingham, Siegfried Wassertheurer, Thomas Weber, Berend E Westerhof, Peter H Charlton

**Affiliations:** 1 Laboratoire Analyse, Géométrie et Applications, Université Sorbonne Paris Nord, Paris, France; 2 Axelife, Paris, France; 3 Department of Public Health and Primary Care, University of Cambridge, Cambridge CB1 8RN, United Kingdom; 4 Research Centre for Biomedical Engineering, City, University of London, London EC1V 0HB, United Kingdom; 5 Institute of Clinical Physiology, Italian National Research Council (CNR), Pisa, Italy; 6 Vall d’Hebron Institut de Recerca (VHIR), Barcelona, Spain; 7 CIBER-CV, Instituto de Salud Carlos III, Madrid, Spain; 8 University Hospital Galway, Cardiothoracic Surgery, Galway, Ireland; 9 Clinic of Cardiac and Vascular Diseases, Faculty of Medicine, Vilnius University, Vilnius, Lithuania; 10 Polytechnic University of Coimbra, Coimbra Health School, Rua 5 de Outubro—S. Martinho do Bispo, Apartado 7006, 3046-854 Coimbra, Portugal; 11 Department of Medical, Surgical and Health Sciences, University of Trieste, Trieste, Italy; 12 Istituto Auxologico Italiano, IRCCS, Milan, Italy; 13 Macquarie University, Sydney, Australia; 14 The George Institute for Global Health, Sydney, Australia; 15 LCOMS, Université de Lorraine, F-57000 Metz, France; 16 Faculty of Medicine, Aristotle University of Thessaloniki, Thessaloniki, Greece; 17 Faculty of Medicine, University of Belgrade, Belgrade, Serbia; 18 Department of Family Medicine, Semmelweis University, Budapest, Hungary; 19 Department of Biomedical Engineering, School of Biomedical Engineering and Imaging Sciences, King’s College London, London SE1 7EU, United Kingdom; 20 Laboratory for Biomedical Microtechnology, Department of Microsystems Engineering—IMTEK, IMBIT—NeuroProbes, BrainLinks-BrainTools Center, University of Freiburg, Freiburg, Germany; 21 Bernstein Center Freiburg, University of Freiburg, Freiburg, Germany; 22 Department of Biomedical Engineering, Cardiovascular Research Institute Maastricht (CARIM), Maastricht University, Maastricht, Netherlands; 23 GROW Research Institute for Oncology and Reproduction, Maastricht University, Maastricht, Netherlands; 24 Macquarie Medical School, Faculty of Medicine, Health and Human Sciences, Macquarie University, Sydney, Australia; 25 INSERM U970 Team 7, Paris Cardiovascular Research Centre; 26 PARCC, University Paris Descartes, AP-HP, Pharmacology Unit, Hôpital Européen Georges Pompidou, 56 Rue Leblanc, Paris 75015, France; 27 Cardiology Department, Klinikum Wels-Grieskirchen, Wels, Austria; 28 Center for Health & Bioresources, Medical Signal Analysis, AIT Austrian Institute of Technology GmbH, Vienna, Austria; 29 Healthcare Technology Innovation Centre, IIT Madras, Chennai 600 113, India; 30 Department of Electrical Engineering, Indian Institute of Technology Madras, Chennai 600 036, India; 31 Research Centre for Intelligent Healthcare, Coventry University, Coventry CV1 5RW, United Kingdom; 32 Faculty of Medical Sciences, Newcastle University, Newcastle upon Tyne NE2 4HH, United Kingdom; 33 MRC Unit for Lifelong Health and Ageing at UCL, 1–19 Torrington Place, London WC1E 7HB, UK; 34 Department of Medicine and Surgery, University of Perugia, Perugia, Italy; 35 Unit of Internal Medicine, ‘Santa Maria’ Terni Hospital, Terni, Italy; 36 Department of Medical and Surgical Sciences, University of Bologna, Bologna, Italy; 37 IRCCS Azienda Ospedaliero-Universitaria di Bologna Policlinico Sant’Orsola, Bologna, Italy; 38 Department of Internal Medicine, Maastricht University, Maastricht, The Netherlands; 39 Department of Vascular Medicine, University Medical Center Utrecht, Utrecht, The Netherlands; 40 Northern Vascular Centre, Freeman Hospital, Newcastle upon Tyne NE7 7DN, United Kingdom; 41 Menzies Institute for Medical Research, University of Tasmania, Hobart, Australia; 42 Stanford University, Stanford, California, United States; 43 Swiss Federal Institute of Technology of Lausanne, Lausanne, Switzerland; 44 Department of Heart Disease, Haukeland University Hospital and Department of Clinical Science, University of Bergen, Bergen, Norway; 45 Institute of Social Medicine, Epidemiology and Health Economics, Charitè—Universitätsmedizin Berlin, Humboldt-Universität zu Berlin, and Berlin Institute of Health, Berlin, Germany; 46 Department of Pulmonary Medicine, Amsterdam Cardiovascular Sciences, Amsterdam UMC, Vrije Universiteit Amsterdam, Amsterdam, The Netherlands; 47 Department of Neonatology, Radboud University Medical Center, Radboud Institute for Health Sciences, Amalia Children’s Hospital, Nijmegen, The Netherlands; 48 Cardiovascular Medicine Unit, IRCCS Azienda Ospedaliero-Universitaria di Bologna, 40138 Bologna, Italy

**Keywords:** ageing, arterial stiffness, blood pressure, cardiovascular, commercialisation, prevention, pulse wave velocity

## Abstract

Vascular ageing (vascular ageing) is the deterioration of arterial structure and function which occurs naturally with age, and which can be accelerated with disease. Measurements of vascular ageing are emerging as markers of cardiovascular risk, with potential applications in disease diagnosis and prognosis, and for guiding treatments. However, vascular ageing is not yet routinely assessed in clinical practice. A key step towards this is the development of technologies to assess vascular ageing. In this Roadmap, experts discuss several aspects of this process, including: measurement technologies; the development pipeline; clinical applications; and future research directions. The Roadmap summarises the state of the art, outlines the major challenges to overcome, and identifies potential future research directions to address these challenges.

## 1. Introduction

### Serena Zanelli^1,2^ and Peter H Charlton^3,4^

^1^ Laboratoire Analyze, Geometrie et Applications, University Sorbonne Paris Nord, Paris, France

^2^ Axelife, Paris, France

^3^ Department of Public Health and Primary Care, University of Cambridge, Cambridge CB1 8RN, United Kingdom

^4^ Research Centre for Biomedical Engineering, City, University of London, London EC1V 0HB, United Kingdom

Cardiovascular disease (CVD) is the leading cause of death worldwide, accounting for approximately 17.9 million deaths per year (Roth *et al* 2017). Vascular ageing (VA) is a concept capturing the changes to the vascular structure and function which occur naturally with age (Nilsson 2015) and can be accelerated in disease (Climie *et al* 2023). Specific aspects of vascular ageing can be measured, such as changes in arterial elasticity or vessel lumen area. It may be helpful to assess vascular ageing in clinical practice, as measurements of vascular ageing have been found to be predictive of cardiovascular (CV) events and all-cause mortality, independently of classical CV risk factors (Vlachopoulos *et al* 2010, Ben-Shlomo *et al* 2014). However, vascular ageing is not yet widely assessed in routine practice (Climie *et al* 2023). A key step towards incorporating vascular ageing assessment in routine practice is the development of technologies to assess vascular ageing (Climie *et al* 2020).

### Overview

**Figure 1.**
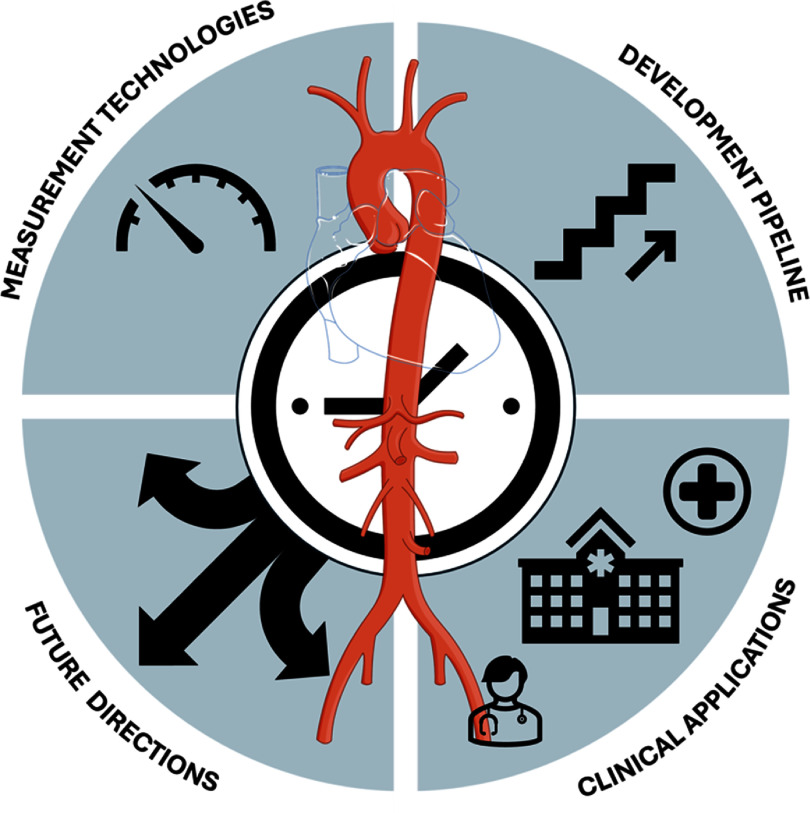
The four areas of the Roadmap. Source: Reproduced from Servier Medical Art. CC BY 4.0.

In this Roadmap, experts provide their perspectives on the development of technologies to assess vascular ageing. Its purpose is to guide future research and development (R&D) in the field, focusing on specific areas of opportunity for the research community. The Roadmap consists of short sections on topics ranging from specific measurement technologies to particular clinical applications. Each section stands alone, offering a summary of the state of the art, the major challenges to overcome, and the scientific and technological breakthroughs that could potentially address these challenges. The sections are categorised into four areas, as illustrated in figure 1.

(1) **Measurement technologies:** A wide range of measurement technologies to assess vascular ageing are presented, including imaging techniques, technologies suitable for use in the clinic, and wearable technologies.
(2) **Development pipeline:** The methods used to develop devices and translate them into clinical practice are discussed. The methods span the development pipeline, from early-stage research to commercialisation.
(3) **Clinical applications:** Key clinical applications of vascular ageing technologies are presented, encompassing diseases affecting the heart, brain, and blood vessels.
(4) **Future research directions:** Key areas for future research and innovation are discussed.

The sections are each self-contained, allowing readers to delve directly into specific sections of interest, without the need to read the entire Roadmap from beginning to end.

### Themes

Some common themes emerge throughout the Roadmap:

• **The ideal measurement technology:** The authors of the Roadmap are clear in their view that there are several measurement technologies available for assessing vascular age with differing capabilities and advantages and disadvantages. Yet, none of these technologies are seen as the ideal solution. Across the Roadmap, the ideal technology emerges as one which is: low cost; safe (i.e. non-invasive and without the need for ionising radiation); operator-independent; easy to use (with minimal training or expertise required); portable; well tolerated by patients; and quick to take measurements.
• **Requirements for clinical use:** The case for adopting a technology into clinical practice is much strengthened by: validation (evidence that its measurements are reliable, including their repeatability and reproducibility); evidence for its clinical utility (showing added value in comparison to standard care); evidence for its acceptability (that the device is acceptable to patients); and evidence for its cost-effectiveness.
• **Identifying potential use cases:** Several potential use cases for vascular ageing technologies emerge through the Roadmap, including: prognosis and risk stratification; diagnosis (including early diagnosis of diseases potentially through screening, distinguishing between different diseases, and prompting treatment); targeting therapies according to vascular ageing measurements; and phenotyping patients.
• **Communicating with stakeholders in the development process:** Several sections of the Roadmap refer to communicating with stakeholders in the development process, highlighting the need to: facilitate communication between different stakeholders; promote awareness of the importance of vascular ageing; gaining trust from patients and device users; engage policy makers; and effectively communicate with patients about the risks associated with vascular ageing. There is opportunity to develop the academic research culture to increase the level of public and patient involvement in the development process.
• **Opportunities for innovative device design:** Some authors present a vision of innovative devices which measure multiple vascular ageing parameters simultaneously, or even investigate multiple diseases at the same time. Such devices could use multiple sensing modalities to achieve this.
• **The benefits of modelling:** Several sections highlight the potential benefits of using CV models in the development process, such as to aid the development of pulse wave analysis (PWA) algorithms or to help better understand the physiological origins of pulse waves (PW).

### Challenges and solutions

Several key challenges and potential solutions emerge from the Roadmap:

• **Conducting clinical studies:** Clinical studies will underpin the translation of vascular ageing technologies into clinical practice. Validation studies are required to assess agreement between vascular ageing measurements and reference parameters. Studies assessing the effectiveness and cost-effectiveness of interventions based on vascular ageing measurements will be essential. Such studies should include a diversity of subjects to understand the clinical utility of technologies across different groups (e.g. across the sexes, across ages, across different body types, and across different pathologies such as atrial fibrillation (AF)). Pragmatic study designs may help facilitate large-scale studies whilst minimising study costs.
• **Ensuring measurements are accurate:** There are several challenges to ensuring measurements of vascular ageing are accurate. First, signals are susceptible to artefacts which can obscure the phenomena of interest. Automated quality assessment algorithms can identify artefact-free measurements suitable for analysis. Second, physiological variability leads to beat-by-beat variability in signals and the phenomena of interest, which should be accounted for in analyses. Third, measurements can be operator-dependent, and (semi-) automation of data acquisition and/or analysis may help reduce inter-operator variability (and have the added benefit of reducing the level of training required).
• **Obtaining measurements in daily life:** Assessments of vascular ageing have traditionally been largely confined to clinical settings. Wearable and non-contact devices may provide opportunity to perform assessments unobtrusively and longitudinally in daily life, providing clinicians with a more holistic view of the vascular status of the monitored subject. These technologies could enable measurements across large numbers of subjects, and facilitate large-scale clinical studies.
• **Creating datasets:** Datasets facilitate the development of vascular ageing technologies, whether during device design (e.g. development of signal processing algorithms), clinical translation (e.g. development of reference values), or the assessment of clinical utility and cost-effectiveness. The field would benefit from more freely available datasets, which should include a diversity of subjects, and may include raw physiological signals for algorithm development. In addition, generative models could be used to generate data starting from a smaller dataset. Synthetic data could then be used to refine vascular ageing assessment techniques or to investigate fields where the real-world data are unbalanced (e.g. imbalances in sex and gender).
• **Establishing reference values:** There is a need for reference values for vascular structure and function assessments, which should be age- and sex-specific, and be created not just for adults but also young people.
• **Developing standards and definitions:** The field would benefit from further development of standards and definitions, such as: reference standard(s) for vascular ageing assessments; device validation protocols; measurement protocols; and definitions of key concepts such as early vascular ageing (EVA). The development of scientific consensus documents would greatly aid this process.
• **Developing medical devices:** Several Roadmap sections discuss the challenges of developing technologies to assess vascular ageing, which are largely common to all medical device development. These include: the difficulties of commercialising academic research; the need to meet regulatory requirements; and the cost of developing devices. These challenges can be addressed through education about the development pipeline, including an awareness of what is required at each stage and when translational activities should be incorporated.
• **Furthering our understanding of underlying theories:** It is beneficial to further our understanding of the theories behind measurement technologies, such as the physiological mechanisms underpinning measurements, and the assumptions on which measurements are based (e.g. the assumptions behind single-point and two-point measurements, and the dependency of measurements on age and blood pressure (BP)). A more detailed understanding could help translate technologies into pressing clinical needs, such as establishing the basis for links between arterial stiffness and dementia. Physiological modelling may provide valuable insights to advance our understanding.

### Outlook

Technologies to assess vascular ageing have potential to be valuable tools to aid diagnosis and prognosis, and to help guide treatments. Whilst much progress has been made in the development of such technologies, several challenges remain to translate vascular ageing assessments in routine clinical practice. The research directions outlined in this Roadmap will help to address these challenges. Ultimately, the successful development of vascular ageing technologies will be a multi-disciplinary effort, requiring the input of academic and commercial researchers, clinicians, and patients alike.

Vascular ageing research is particularly timely given its potential role in tackling various age-related diseases, in the light of ageing populations. Industry collaboration is essential for translating vascular ageing research into tangible products and services that benefit patients, clinicians, and society as a whole. By leveraging the expertise and resources of industry partners, researchers can accelerate the translation of scientific discoveries into clinical practice and address unmet needs in the field of vascular ageing.

### Acknowledgments

This work was supported by COST Action CA18216 VascAgeNet, supported by COST (European Cooperation in Science and Technology, www.cost.eu). P H C acknowledges funding from the British Heart Foundation (FS/20/20/34626).

### Conflicts of interest

S Zanelli collaborates with Axelife, a company that designs and develops devices for assessing vascular ageing.

## Measurement technologies

## 2. Ultrasound (US) imaging

### Maria Raffaella Martina^1^ and Elisabetta Bianchini^1^

^1^ Institute of Clinical Physiology, Italian National Research Council (CNR), Pisa, Italy

### Status

Non-invasive US imaging is an affordable and safe technique, adopted since the mid-20th century in clinical and research settings, exploiting acoustic energy at not audible frequencies (higher than 20 kHz). This approach uses piezoelectric components to generate US waves, which hit biological structures, and then return where they are collected through the same transducer. Interaction with tissues induces reflection, refraction and scattering of the original signal that can be processed to obtain information (e.g., an image sequence) of a region of interest (ROI) in real-time.

Standard US equipment includes signal generation and processing units, a monitor, and a hand-held probe in contact with the subject’s body through a water-based gel which allows US waves’ passage. Transducers, which can differ in shape, number of elements, and central frequency, are adopted depending on application and site, considering that lower frequencies provide lower spatial resolution but have greater penetration depth. Non-invasive solutions suitable for imaging of various vascular sites are available, including for example curved array for abdominal applications or phased array for cardiac and cerebral analysis. In addition, linear arrays are commonly used for vascular imaging with a frequency range of around 7–18 MHz. Interestingly, contrast agents consisting of encapsulated microbubbles containing gases can be used for contrast enhancement e.g., in atherosclerotic plaque analysis. In addition, when exploiting a working principle called Doppler effect, that relies on the frequency content of US waves, velocities of vessels’ blood can be also quantified. Within this section we are focusing on non-invasive assessment, but is worth noting that, also intravascular US approaches are available, working with a frequency range of around 20–60 MHz, and based on a dedicated catheter producing cross-sectional images for the investigation of e.g., coronary and pulmonary arteries. For a detailed overview of US principles and vascular applications, we refer the reader to the following sources: (Hoskins *et al* 2019) and (Bianchini *et al* 2023a).

**Figure 2.**
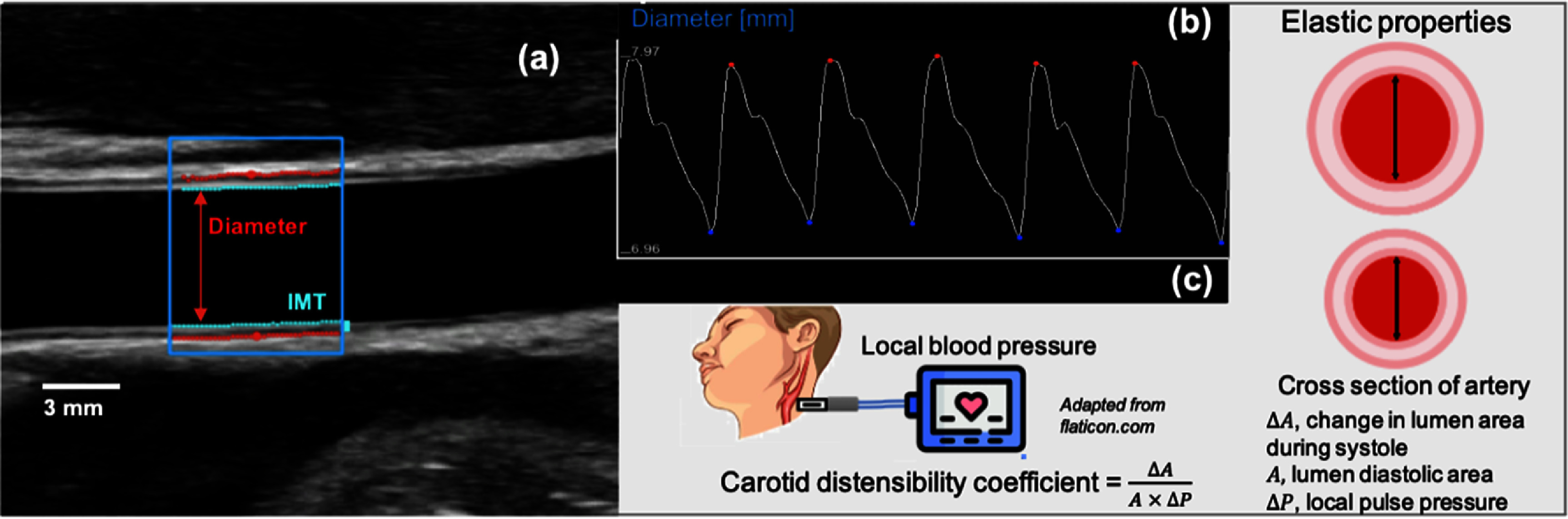
(a) Standard longitudinal B-mode US image of a common carotid artery processed by software based on a contour tracking algorithm measuring diameter and intima media thickness (IMT). (b) Measurements of instantaneous diameter. (c) Applanation tonometry for carotid blood pressure estimation, calibrated through brachial pressure assessment, that, combined with the US derived analysis, provides elastic parameters.

Non-invasive US can be used to obtain information in key superficial arteries associated with vascular disorders, such as arteriosclerosis related to the reduced arterial elasticity or the building of atherosclerotic plaque which modifies the morphology of a vessel. For example, the carotid artery is a large and central vessel, easily accessed by US, whose alterations can play a role in prevention, diagnosis and monitoring of relevant diseases (Engelen *et al* 2013). Femoral, brachial and some aortic segments can be imaged as well, providing vascular information at these sites (Bossuyt *et al* 2015). In fact, US techniques can provide a local structural and functional assessment: for example, diameter, intima-media thickness (IMT) and, assuming the cross-section of the artery to be circular, elastic parameters, such as the distensibility coefficient, (Laurent *et al* 2006) can be assessed simultaneously by processing longitudinal B-mode scans (see figure 2) (Bianchini *et al* 2023a). In addition, vascular deformation patterns can be obtained on short-axis imaging by two-dimensional speckle-tracking strain that quantifies wall circumferential deformation within the cardiac cycle.

Due to its intrinsic peculiarities, non-invasive US is able to provide a multi-site safe assessment along the arterial tree to obtain a heuristic evaluation of vascular ageing. However, this approach is currently underused, in part because of its usability and operator-dependency. Technological advances are increasing the utility of US in clinical and research settings, thanks for instance to portable probes (i.e. compact hand-held probes that can be directly connected with a computer or a mobile platform via USB or wireless) and reliable advanced processing algorithms whose combination can provide innovative architecture solutions.

### Current and future challenges

Challenges in the field are related to increasing the potential of non-invasive US in applications for vascular ageing assessment. In particular, key challenges are focused on the needs to decrease the inherent operator-dependency, spread the adoption of technological advances in practice, improve the image quality and resolution, and develop shared standardisation protocols.

Great advances have been achieved in the last years to address the above mentioned technological and operative challenges. In particular, semi-automatic image processing systems based on robust algorithms (Bianchini *et al* 2023a) were introduced to improve the reliability of the final measurement by reducing e.g. the impact of the operator-variability on the final results. Moreover, three-dimensional imaging has been introduced, a promising tool for vessel morphology assessment overcoming limitations of two-dimensional imaging related to the selection of a single section (Bredahl *et al* 2013, Ghulam *et al* 2020, Alzahrani *et al* 2023).

Other key aspects to take into consideration are the usability and accessibility of technology. Currently, hardware and software solutions are often fragmented, physically and commercially, particularly for more innovative applications. Conversely, integrated devices might provide the two-fold benefit of more easily available and usable approaches suiting healthcare systems’ needs e.g. for analysis related to screening campaigns for vascular assessment or even for wearable applications, based on pioneering US tools for continuous imaging of internal organs. Advances in this direction have been done by the new generation of low-cost portable US scanners available on the market, and the development of innovative US based patches (Wang *et al* 2022), but further technological steps are needed for the effective hardware-software integration required in dedicated CV applications.

Some specific further challenges are related to the development of advanced US based technologies able to provide functional and structural insights in smaller time and space scales. Indeed, increased temporal or spatial resolution might allow to detect and analyse structures not visible with standard architectures or to record events of short duration. This kind of approach introduces further requirements in terms of e.g. data acquisition, processing efficiency, equipment architecture.

Finally, standardised US acquisition protocols and guidelines by scientific expert committees are crucial requirements for any clinical application and, especially for the more innovative methods, a shared approach is lacking. The usefulness of standardisation should be taken into account starting from the validation process in order to provide clear and comparable criteria for performance assessment.

### Advances in science and technology to meet challenges

**Figure 3.**
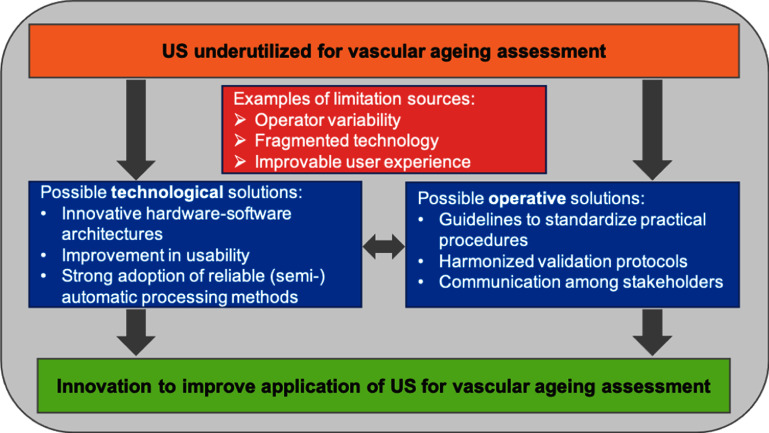
Main limitations and possible solutions to meet challenges related to the application of US techniques in clinical practice for vascular ageing assessment.

Innovation in terms of technological development in hardware and software solutions, as well in their integration, can address some of the above mentioned challenges, as schematized in figure 3.

Advanced US architectures able to provide higher spatial (ultra-high frequency, UHF) or temporal (Ultrafast) resolution compared to standard US equipments have been introduced.

Specifically, UHF US technique exploits transducer with operating frequencies up to 70 MHz providing non-invasive high image quality of superficial vascular sites (Izzetti *et al* 2021). This approach can open windows on structures and sites that have not been investigated so far, such as digital arteries, relevant in particular diseases and to exploring systemic-peripheral vascular behaviour interaction (Poli *et al* 2022).

On the other hand, ultrafast US is able to record data with high sampling frequency (frame-rates up to ten thousand frames per second), thus allowing the analysis of rapid changes in a vascular site, such as the study of shear wave propagation, an estimation of local arterial stiffness (Marais *et al* 2019) and PW imaging (Li *et al* 2019).

In order to be translated to the practice, advances in equipment architecture must be flanked by improvements in reliability and usability. For example, effective implementation of a mobile tool for vascular assessment requires the development of combined hardware-software solutions able to integrate portable commercial probes with precise US data processing algorithms (Francesconi *et al* 2021).

Robust algorithmic approaches in general might be able to provide solid data for determination of reference values where missing, a crucial step for translation from research to practice (Engelen *et al* 2013, Bossuyt *et al* 2015). Novel applications exploiting digital signal processing can also introduce advanced vascular analysis: for example, emerging method about peripheral arterial hemodynamic states can contribute to face challenges related to the need for better quantification of complex arterial hemodynamic patterns, such as US vector flow imaging (Baun 2021).

It is an imperative to mention also the potential impact of artificial intelligence (AI). On one hand, US features’ quantification and the adoption of machine learning (ML) classifiers might help the investigation of clinically relevant parameters (Avanzo *et al* 2020), a challenging task due to the need of standardised big data; and, the combination of AI based approaches with multi-parameter information obtainable by US may provide, in one single imaging modality, tools able to improve clinical diagnosis. On the other hand, AI-based approaches might improve usability, by augmenting repeatability and time efficiency and providing the possibility to automate such methods.

### Concluding remarks

Non-invasive US imaging is a safe and relatively low-cost approach capable of providing multi-parameter data for an integrated assessment of vascular structure and function. Reliable and usable technologies based on the integration of standard and innovative architectures with (semi-) automatic processing approaches can expand the potentiality of US vascular ageing assessment in practice.

In this context, dialogue and collaboration among clinicians, researchers and industry in the field is necessary in order to harmonize methods and procedures and thus lead to a personalized description of a subject’s vascular ageing.

### Acknowledgments

This paper is based upon work from the COST Action CA18216 ‘Network for Research in Vascular Ageing’ supported by COST (European Cooperation in Science and Technology, www.cost.eu).

### Conflict of interests

Elisabetta Bianchini is co-founder of QUIPU s.r.l., Pisa, Italy a spin-off company of the Italian National Research Council and the University of Pisa developing software medical devices.

## 3. Computed tomography (CT) and magnetic resonance imaging (MRI)

### Andrea Guala

Vall d’Hebron Institut de Recerca (VHIR), Barcelona, Spain

CIBER-CV, Instituto de Salud Carlos III, Madrid, Spain

### Status

CT and cardiovascular magnetic resonance (CMR) are advanced, relatively-recent medical imaging modalities offering a broad range of possibilities for research and clinical practice in vascular ageing. Indeed, they can provide high-resolution, time-resolved images of virtually all regions of the circulation, with excellent reproducibility (Bianchini *et al* 2023b). These modalities are thus considered standards for the 3D evaluation of vascular geometrical characteristics, such as diameter, volume, length, and tortuosity, among others, and are often key for the assessment of the impact of vascular characteristics on target organs, such as the heart, brain, and kidneys. CMR also offers high-quality assessment of blood velocity, while CT is the reference technique for the evaluation of calcium deposit (Arnett *et al* 2019).

CMR exploits the heterogeneity of the magnetic properties of tissues to form images. In particular, it creates images by applying (i) a strong, static magnetic field, which orients nuclei along a main axis, and (ii) lower-intensity, transversal magnetic field components, which dynamically perturb the static magnetic field. The temporal evolution of these perturbations is read by a receiving coil and used to form images and to assess blood velocity. Conversely, CT exploits the heterogeneity in the attenuation of x-rays in tissues. A CT scanner sends x-rays through the body and quantifies how much is retained by specific tissues.

Nonetheless, clinical guidelines as well as current clinical practice exploit only a limited part of what could be achieved by these imaging modalities, while an in-depth analysis of acquired images for opportunistic screening is rarely followed. In particular, regarding potential biomarkers for vascular ageing assessment by these modalities, only arterial diameter and coronary calcium score (CAC) are effectively included in clinical guidelines. There are several reasons explaining the relatively-limited clinical impact of these imaging techniques for vascular ageing. Firstly, they are substantially more expensive than US (i.e. echocardiography, the most used imaging modality), while image interpretation requires specific training and, often, considerable post-processing, which is frequently non-standardised. This results in substantial heterogeneity in their availability, with low and lower-middle income countries, but also remote areas of wealthy countries, having very limited access (Ogbole *et al* 2018). Differences in acquisition and post-processing often result in limited reproducibility across clinical centres. Moreover, CT exposes patients to ionizing radiations, while both CT and CMR often require the administration of external contrast agent, whose use has to be evaluated in light of the potential clinical benefit. Given these characteristics and problems, further work is needed to bring the possibilities offered by CT and CMR for the assessment of vascular ageing in clinical practice.

### Current and future challenges

Ongoing work can be separated in two main aims: from one side to provide stronger clinical evidence of the advantages of acquiring images with these imaging techniques, and on the other side, optimising their use for greater benefit to patients.

Regarding clinical evidence, substantial prognostic value has been reported for several descriptors of arterial ageing acquired by CMR and CT. These descriptors can be grouped into three classes: arterial size, arterial stiffness and calcium deposits. Regarding arterial size, the strongest prognostic value has been reported for aortic diameter, specifically for adverse CV events and all-cause mortality (Norman *et al* 2004, Duncan *et al* 2012, Qazi *et al* 2017). Notably, this prognostic value was demonstrated for a range of physiological diameters, likely showing the pre-clinical impact of ageing on this vessel. With respect to arterial stiffness, aortic PW velocity (PWV) and distensibility by CMR have been shown to be significant predictors of adverse CV (Redheuil *et al* 2014, Ohyama *et al* 2017), cardiac (Maroules *et al* 2014) and extra-cardiac (Maroules *et al* 2014) events and all-cause mortality (Redheuil *et al* 2014) in patients free from overt CV disease. Finally, CAC by CT has been shown to have a strong predictive value for atherosclerotic CVD and death in the general population, being thus suggested for risk-stratification in primary prevention (Arnett *et al* 2019).

Despite all these biomarkers having been shown to be useful for risk-stratification in large, prospective general-population studies, their use is hampered by technical, economic and practical challenges. Several advances are required to meet these challenges, as detailed below.

### Advances in science and technology to meet challenges

A number of advances are required to meet the technical, economical and practical challenges hampering the use of vascular ageing biomarkers obtained by CT and CMR in clinical practice.

Firstly, expanding the availability of these imaging modalities. Currently, the modalities are not available in many parts of the world. As introduced, a key issue is cost: according to Catalan public healthcare provider, the cost of CT and CMR studies is 3.5 and 5 times that of an US study, respectively. This is one of the key reasons limiting the use of these imaging modalities. To reduce cost, the research community is working on reducing acquisition time and reducing the need for the administration of contrast agents. Another key aspect related to the use of these imaging modalities is the optimization of their use, meaning improving the identification of patients who are likely to benefit most from these tests.

Another set of key challenges arise in image acquisition and analysis. Differences in scanner, sequence and image modality have been shown to impact the absolute value of certain characteristics, such as arterial diameter, while most of these biomarkers are extracted via substantial post-processing. For diameter post-processing means applying multi-planar reconstruction to 3D images to visualise a plane perpendicular to the vessel’s main axis. Despite being simple, this process requires time and impacts reproducibility (Dux-Santoy *et al* 2022). Fully-automatic segmentation of vessel boundary by machine learning algorithms is expected to provide a possible solution for inter-observer variability (Garrido-oliver *et al* 2022). For PWV and distensibility post-processing aims to track the propagation of velocity waves and arterial boundaries, respectively. Although several methods have been (and still are being) developed, none can be considered absolutely true, and there is no consensus for a method to be adopted by the whole community. Moreover, there is limited availability of commercial software to assess these biomarkers. Conversely, there is consensus on the parameter for the acquisition of images to compute CAC. Despite there being several possible ways to compute an overall CAC, the Agatston score is by far the most used, and considered a reference. In general, freely-available, open datasets may be helpful for benchmarking post-processing, and thus assessing the impact of these image analysis techniques on absolute values.

Finally, the community is moving fast in the proposition of alternatives to the administration of contrast agents as well as in the reduction of ionizing radiation exposure. The former is pursued by enhancing the regions of blood flow either by using velocity encoding or artificially via machine learning algorithms.

### Concluding remarks

CMR and CT offer great opportunities for research and clinical practice for the assessment of arterial ageing. Still, the relatively low availability, high cost and required expertise are key factors hampering its widespread use. The clinical and technical research community is working on reducing cost, radiation and contrast agent exposure, and time and variability in the analysis, likely solving many of the current problems.

### Acknowledgments

Guala A has received funding from ‘la Caixa’ Foundation (LCF/BQ/PR22/11920008).

## 4. Oscillometry techniques

### Dave Veerasingam^1^, Petras Navickas^2^ and Telmo Pereira^3^

^1^ University Hospital Galway, Cardiothoracic Surgery, Galway, Ireland

^2^ Clinic of Cardiac and Vascular Diseases, Faculty of Medicine, Vilnius University, Vilnius, Lithuania

^3^ Polytechnic University of Coimbra, Coimbra Health School, Rua 5 de Outubro—S. Martinho do Bispo, Apartado 7006, 3046–854 Coimbra, Portugal

### Status

**Figure 4.**
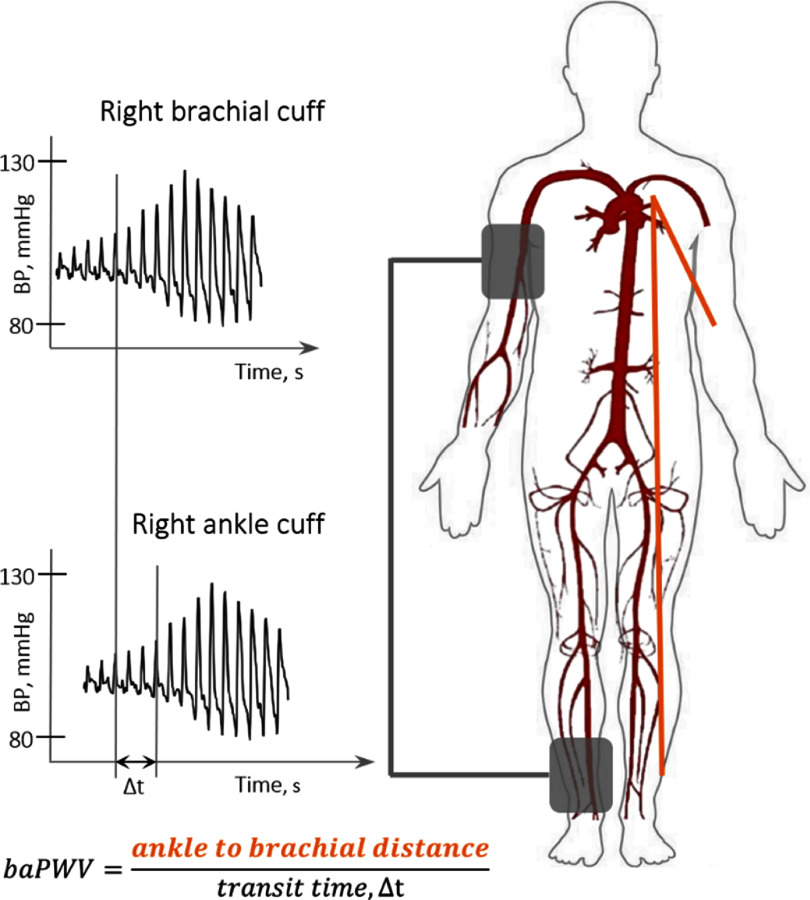
Two-site oscillometric arterial stiffness assessment by calculating the brachial–ankle pulse wave velocity (baPWV) through the measurement of the transit time between the brachial artery and tibial artery through the oscillometric amplitude. *Definitions:* BP—blood pressure; baPWV—brachial–ankle pulse wave velocity.

**Figure 5.**
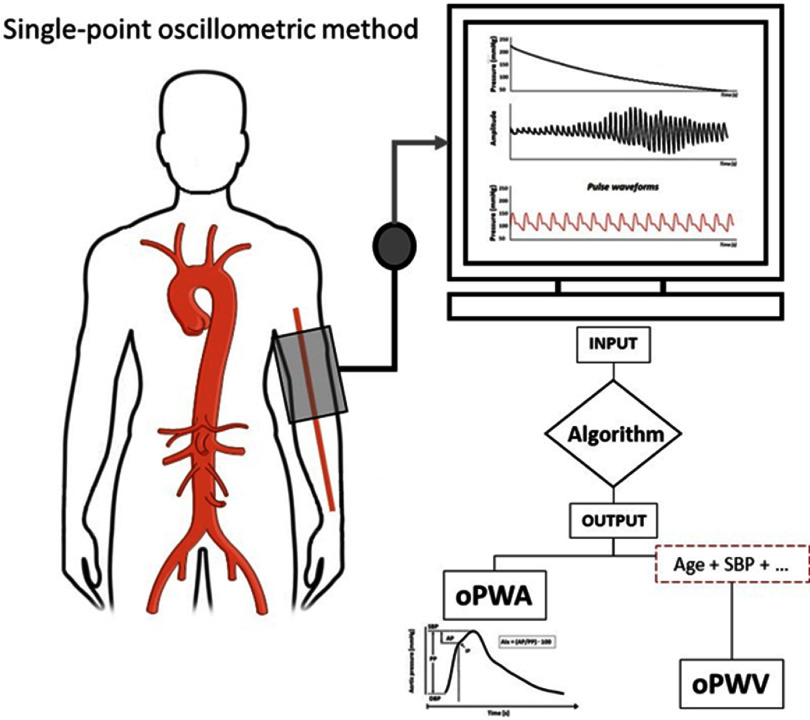
Single-site oscillometric arterial stiffness assessment method by the combination of cuff oscillometry, pulse wave analysis and validated algorithms including covariates such as age and systolic blood pressure. *Definitions:* oPWA—oscillometric pulse wave analysis; oPWV—oscillometric pulse wave velocity; SBP—brachial systolic blood pressure.

Vascular ageing due to structural changes in the arteries leads to reduced vascular compliance and increased arterial stiffness, which can be assessed using surrogate parameters of PWA by many non-invasive methods: tonometry, oscillometry (either single or two sites) (figures 4 and 5), imaging techniques (MRI, echocardiography), photoplethysmography (PPG) as well as the use of estimated values of PWV. Although tonometric carotid-femoral PWV (cfPWV) has generally been considered a reference standard of non-invasive vascular ageing assessment, validated devices, which combine cuff oscillometry and PWA to estimate PWV on a single oscillometric BP measurement, are lately gaining traction since they are non-invasive, easy-to-use, cost-effective, portable, operator-independent and, therefore, especially suitable for use in everyday clinical practice. Nonetheless, the epidemiologic data regarding the use of these methods remains significantly lower compared to tonometric methods.

Oscillometric PWV is a non-invasive cuff-based measurement of blood flow velocity in the arteries, based on the principle that changes in BP propagate through the arterial system as pressure waves, which are recorded as oscillations of the pressure in the cuff. There are two types of devices to estimate aortic PWV: two-point measurement devices that determine the difference in travel time of pulse wave between two different sensor positions (figure 4) and single-point measurement devices that determine the time difference between the forward and backward wave considering the wave reflection model on one site (figure 5), based on PWA and wave separation analysis algorithms (Laurent *et al* 2016). Single-site oscillometric arterial stiffness assessment methods have major advantages since brachial and central BP can be measured at the same time, in addition, estimation of arterial stiffness only requires modest increase in measurement time. Therefore, 24 h monitoring of central BP and arterial stiffness, which could provide additional information, is possible. Their limits have nevertheless to be taken into account since the theory behind estimation of arterial stiffness from single brachial measurement is based on a mathematical model combining several parameters from PWA and wave separation analysis together with age and BP.

Overall increased PWV has been shown to predict CV, and in some cases all-cause, mortality in individuals with hypertension, end-stage kidney disease, diabetes mellitus, and in the general population (Vlachopoulos *et al* 2010). What is more, it has been demonstrated that arterial stiffness, as a biomarker of CV risk prediction, is independently associated with outcomes, and improves discrimination, calibration as well as net reclassification.

### Current and future challenges

Oscillometric devices (ODs) are currently used to measure BP in routine clinical practice. ODs that can also measure parameters of vascular ageing would be of added benefit in cardiovascular (CV) risk detection and management. The routine clinical practice of CV risk prediction and events are based on risk scores pertaining to age and traditional risk factors. Vascular ageing assessment in terms of arterial stiffness, namely cfPWV parameter, has been shown to further improve on existing CV risk scores and is well documented and accepted (Boutouyrie and Bruno 2018). The Clinical practice guidelines of 2018 European Society of Hypertension/European Society of Cardiology guidelines allowed for PWV utility to detect hypertensive mediated organ damage (HMOD) (Williams *et al* 2018). CV risk increases with the presence of HMOD, some reversed by antihypertensive treatment, when used early, but may become irreversible despite improved BP control when hypertension is long standing with age (Kjeldsen *et al* 2018). There are scant published clinical outcome trials above existing studies associated with weight reduction and increased exercise. ODs can measure PWV with a combination of PWA and the oscillometric property of the artery studied at diastolic and suprasystolic pressures by using transducers in inflatable cuffs. Cuffs placed peripherally on the limbs where the artery is palpable over a bone makes it comfortable for the patient without the requirement of groin exposure. Moreover, detection of the femoral pulse is challenging in an obese body habitus. Easy use and reproducibility of ODs not requiring expert personnel makes it one of the most used devices to measure PWV currently. ODs simultaneously measure peripheral blood and pulse pressure; estimated cfPWV; estimated central pulse pressure and augmentation index making utility of these added parameters attractive for the use of ODs in outcome studies related to vascular ageing. ODs that have a cuff with separate PPG sensors in the upper and lower extremities can measure ankle–brachial index (ABI) and brachial–ankle PWV (baPWV) which is also being used in longitudinal studies (Boutouyrie and Bruno 2018). ODs utilise large artery regional PWV measurements and combined large and peripheral arteries (multi-regional PWV measurements) as well as static (single point) PW measurements making the harmonisation of the various PWV parameters challenging. The length and path of the arterial tree traversed is estimated by surface anatomy which furthers compounds acceptability. A recent study involving 1162 subjects to define the agreement between a particular OD measuring PWV and the reference standard tonometric cfPWV measurement in the general population concluded that both values of PWV closely correlated highlighting applicability in the general population (Del Giorno *et al* 2021). Vascular ageing outcome trials using ODs to detect, monitor and guide therapy related to HMOD as well as further evidence for the validation of commercial ODs according to Association for Research into Arterial Structure and Physiology (ARTERY) Society guidelines will help overcome the challenge of routine acceptability (Wilkinson *et al* 2010). The challenge in obtaining the new medical device regulations (MDR) as a Class III medical device (software as a medical device) for the indicated use will be a major step forward in the confident use of ODs in routine clinical practice for vascular ageing assessment.

### Advances in science and technology to meet challenges

The concept of vascular ageing is currently envisioned as a key programmatic axis in CV research, but albeit the cumbersome evidence yielding some of vascular ageing biomarkers as major predictive factors (Vlachopoulos *et al* 2010, Boutouyrie and Bruno 2018), their translation into clinical practice has been relatively disappointing. Several reasons could be pinpointed for this, including technical and time-consuming features of the gold-standard two-point measurement approach, absence of a single, simple and intuitive measure of vascular ageing, and lack of therapeutic approaches specifically aimed at restoring vascular ageing trajectories. Future efforts in science should therefore be directed to tackle these challenges, congenial with the consolidation of vascular ageing as a clinical cornerstone in CV medicine. With this in mind, measures of vascular ageing derived from ODs are a promising approach, mostly for its simplicity, readiness and speed of measure. Notwithstanding, some challenges are yet to be met in order to preserve the strength of more validated approaches in ODs. One major issue equates from the highly dependence of current single-point ODs on proprietary algorithms that are heavily determined by features such as age and BP, therefore making the extrapolated measures of arterial stiffness highly dependent on aspects rather than vascular function per se, invariably leading to a penalised prognostic information above the two conventional risk factors. On the other hand, the proprietary algorithm idiosyncrasies and their intertwining with BP measurement technology may introduce further variability in the estimations, also compromising the comparability amongst technologies. The emergence of algorithms that are able to extract vascular ageing parameters solely based on the waveform characteristics from oscillometric BP measurement in single-point systems (Baulmann *et al* 2022) is definitely a promising step forward to accurately assess these parameters, simultaneously meeting the requirements for everyday clinical practice. Achieving the widespread use of these technologies, and its incorporation in sensors/wearables interconnected with portable smart devices would provide added opportunities to explore the oscillometric arterial waveforms in a big data approach, incorporating machine and deep learning algorithms for feature extraction and classification, conveying further opportunities of sophisticated analysis of the waveform envelope towards the extraction of useful information that would not, otherwise, be accessible (for a thorough review, read (Bikia *et al* 2021a)). This sophisticated processing of the oscillometric arterial waveform in an integrative multimodal methodology including clinical information and multiple individual risk factors, could well pave the way towards the emergence of a single and robust estimate of vascular ageing, further contributing to a broader application of this concept in clinical practice.

### Concluding remarks

Vascular ageing in the form of arterial stiffness measures has the clinical utility of CV risk prediction and is independently associated with outcomes and improves discrimination, calibration as well as net reclassification. Oscillometric arterial stiffness estimates show a good and satisfactory agreement compared to the reference standard (tonometric) PWV assessment. Oscillometric arterial stiffness analysis is a non-invasive, easy-to-use, cost-effective, portable, operator-independent and suitable for use in everyday clinical practice. Notwithstanding the merits of ODs in the science of vascular ageing, further technological developments are needed for the ODs to fully match the accuracy and predictive ability of the gold-standard method, to be readily available for inclusion in daily-life portable smart devices and to benefit from AI capabilities in the form of a vascular ageing single classifier. Also, strict validation requirements for OD-based vascular ageing measurement must be set and compulsory. Therefore, using pooled studies and registries of large numbers of subjects from multidisciplinary research will further advance our understanding and use of vascular ageing assessment into daily clinical practice.

### Acknowledgments

This article is based upon work from the European COST ACTION CA18216 ‘Network for Research in Vascular Ageing’, supported by COST (European Cooperation in Science and Technology, www.cost.eu).

## 5. Applanation tonometry

### Andrea Grillo^1^, Paolo Salvi^2^ and Isabella Tan^3,4^

^1^ Department of Medical, Surgical and Health Sciences, University of Trieste, Trieste, Italy

^2^ Istituto Auxologico Italiano, IRCCS, Milan, Italy

^3^ Macquarie University, Sydney, Australia

^4^ The George Institute for Global Health, Sydney, Australia

### Status

Arterial tonometry has historically been one of the most widely used technologies for assessment of vascular ageing (VA). Deriving from experiments with sphygmography in the late nineteenth century for measuring BP, arterial tonometry received a new interest in the last decades of the twentieth century with the availability of transcutaneous high-fidelity tonometry sensors.

**Figure 6.**
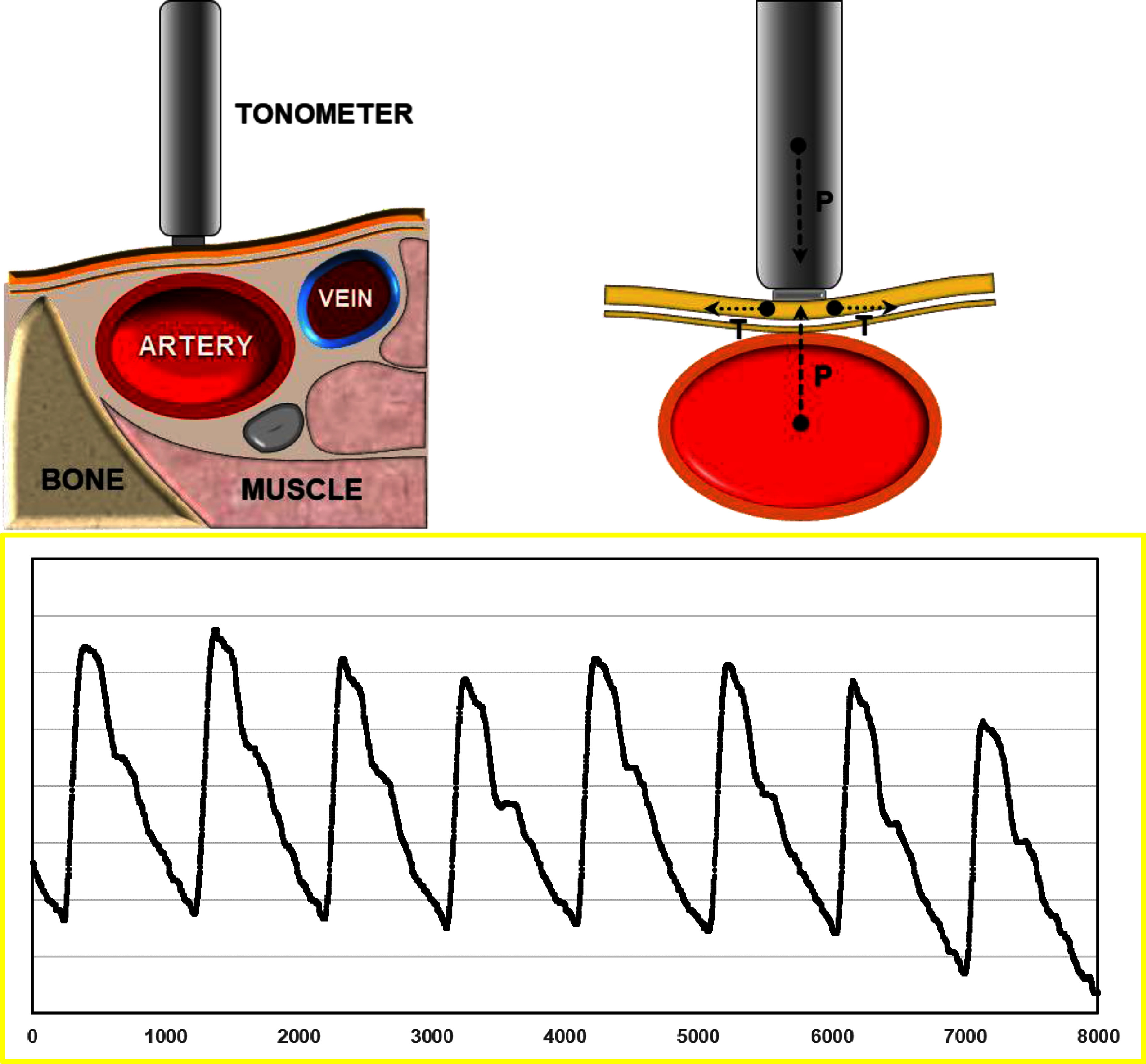
Applanation tonometry. Left panel: transcutaneous arterial tonometry in radial artery. Right panel: applanation tonometry principle: a circular structure with a given pressure inside (artery) is flattened; in this way circumferential pressures are equalized and the sensor records the intra-arterial pressure (*P*). Tensile forces (*T*) are perpendicular to pressure vectors. Lower panel: raw recording of a radial applanation tonometry sampled at 1 kHz.

Tonometry enables the assessment of vascular districts in which an artery runs superficially and may be compressed against underlying supporting structures, such as bones or fibrous fasciae. It is based on the principle of applanation (flattening) and derived from ocular tonometry (Imbert–Fick law), which states that the pressure within a thin-walled sphere is equal to the force required to flatten the sphere’s surface divided by the area of applanation. When an arterial segment is deformed along its vertical axis near the centre line, assuming there is a uniform deformation of the artery along its length, the tonometer is thus able to measure the internal arterial pressure. Figure 6 illustrates the system operation in which an arterial wall, represented by an ideal membrane, is flattened by a force that only depends on the BP and the applanation area, as tensile forces are perpendicular to the pressure vectors. Piezoresistive strain-gages determine the force exerted on the sensor element, which is directly proportional to intra-arterial pressure. This allows for the recording of the dynamic arterial pressure waveforms, which have been shown to be superimposable over those recorded invasively with an intra-arterial catheter when some conditions are satisfied (Eckerle 2006). Unlike ocular tonometry, which measures intraocular pressure directly, arterial tonometry cannot measure intra-arterial pressure but rather only relative changes in pressure. In order to obtain absolute blood pressure (BP) values, arterial tonometry requires calibration with auscultatory or oscillometric BP values.

Miniaturization and development of multiple-element sensors allowed the manufacture of very accurate and small-dimension tonometers that are currently marketed as manual pen probes. A proper operator technique for correct positioning of the probe and the avoidance of motion artefacts are required to obtain a good quality measurement. Averaging of multiple cardiac cycles (from 3 to 10) reduces effects of beat-to-beat variability and removal of small artefacts in the waveform. When optimal measurement conditions are present, arterial applanation tonometry is usually a well-tolerated, reproducible and non-invasive test that can be performed in several arterial segments (carotid, brachial, radial, femoral, posterior tibial and dorsalis pedis arteries) and allow for haemodynamic measurements through arterial pulse wave analysis (PWA).

One leading application of arterial tonometry is in the determination of large artery stiffness. Pioneering studies documented the increase of pulse wave velocity (PWV) with age as the hallmark of stiffening of the aorta (Kelly *et al* 1989). The measurement of carotid to femoral PWV (cfPWV) has early on become the reference method for measuring arterial stiffness, which can be easily obtained by tonometry of the carotid artery and the femoral artery. cfPWV can then be determined by dividing the arterial path length (external distance measurement) by transit time between the foot of the carotid and femoral pressure waveforms (Laurent *et al* 2006). In addition, the analysis of aortic waveform features, such as central BP and wave reflection-related parameters, derived from either carotid artery or radial artery tonometry, is also useful to obtain information about the process of ageing of the vascular tree.

### Current and future challenges

Despite advances in technology of commercially available tonometers and their clinical validation for cardiovascular prevention, the adoption of this method in a wide audience is still limited outside of medical research centres. Some reasons can be attributed to the need of dedicated time and of trained operators, along with the direct and indirect costs of the examination. At this point of its technical development, arterial tonometry has reached a consolidated amount of research that may justify its applicability, and a main challenge will be to promote its use and to include it in clinical guidelines. To achieve this goal, there is still the need to obtain evidence from clinical studies that treatment of patients based on information from tonometry is more effective than current standard care.

There are a large number of tools available to measure vascular ageing, based either on tonometry or on other methodologies, yet there is currently no consensus on how to apply vascular ageing measures. Current evidence based on comparative studies of different methodologies is oriented toward the support of arterial tonometry compared to other measures, in part due to its better replicability and reliability (Grillo *et al* 2018). A methodological consensus is urgently required to guide research in the field of vascular ageing.

A further methodological issue is the way the central aortic PW is defined from arterial tonometry (Salvi *et al* 2015). Two methods are currently available to record the central aortic pressure wave: (1) the ‘direct’ method, in which arterial tonometry from the common carotid artery with proper calibration is used as a surrogate for aortic pressure due to the close proximity of the vessels; (2) the ‘indirect’ method, where central waveform is synthesized from a peripheral arterial waveform (such as brachial or radial waveform obtained from tonometry) with a generalised transfer function. Calibration of the central or peripheral pressure waveforms with an external BP measurement (commonly with cuff-based oscillometric brachial BP) is required for both methods, and how this is done may affect the estimate of central aortic pressures (Sharman *et al* 2017). Again, a consensus is advisable to provide a clear message to a large audience, stating if both the direct or indirect methods are equally valid and interchangeable for clinical application or not, and which method of calibrating the arterial waveform, be it carotid or peripheral, is the most appropriate.

### Advances in science and technology to meet challenges

With the adoption in a clinical setting being the biggest challenge for arterial tonometry, several advances have been realised to make this technique more suitable for its everyday use. Although it is a relatively simple method to learn, obtaining a good quality pulse signal from tonometry requires operator training and dedicated time.

For the measurement of cfPWV, the need to perform applanation tonometry on both carotid and femoral arteries simultaneously, which is not easy to perform for a single operator, was overcome by the use of an ECG-gated technique, or, more recently, by using mechanical holders for positioning the tonometric probe, or by replacing femoral tonometry with a cuff-based acquisition (Butlin and Qasem 2016). This cuff-based evaluation of the peripheral femoral pulse seems to be more suitable for some cultures, where direct examination of the groin area may be considered inappropriate. The use of cuff-based or plethysmography-based methods to evaluate the pulse waveform from peripheral arteries, although more appealing for its ease of use, presents some limitations because of the dampening of measured pulse wave with loss of high-frequency information. Thus, tonometry remains the reference method for the applications where analysis of the components of the waveform is of interest.

Currently, the use of tonometry is advancing on parameters that may be clinically useful in special populations or settings. From the central aortic pressure wave, information can be extracted about the balance between the oxygen supply and demand of the myocardium, namely the subendocardial viability ratio. Originally determined by invasive heart catheterization, this index has recently been improved in its non-invasive formulation by arterial tonometry (Salvi *et al* 2021), and may be particularly relevant in the aged population, where a high risk of myocardial ischemia exist and relevant alterations in the central waveform, such as pressure amplification, have already demonstrated predictive power (Benetos *et al* 2012).

### Concluding remarks

Despite the constant increase in original research on applanation tonometry in the last 20 years, its use for the evaluation and treatment of vascular ageing will become more common if supported by strong clinical evidence. Further studies are necessary to evaluate the best use of arterial tonometry in the clinical setting for quantifying vascular age, with the aim to detect EVA and thus assist in early intervention.

### Acknowledgments

This review was partially supported by the Italian Ministry of Health.

## 6. Photoplethysmography techniques

### Peter H Charlton^1^, Djammaleddine Djeldjli^2^ and Serena Zanelli^3,4^

^1^ Department of Public Health and Primary Care, University of Cambridge, Cambridge CB1 8RN, United Kingdom

^2^ LCOMS, Université de Lorraine, F-57000 Metz, France

^3^ Laboratoire Analyse, Géométrie et Applications, Université Sorbonne Paris Nord, Paris, France

^4^ Axelife, Paris, France

### Status

**Figure 7.**
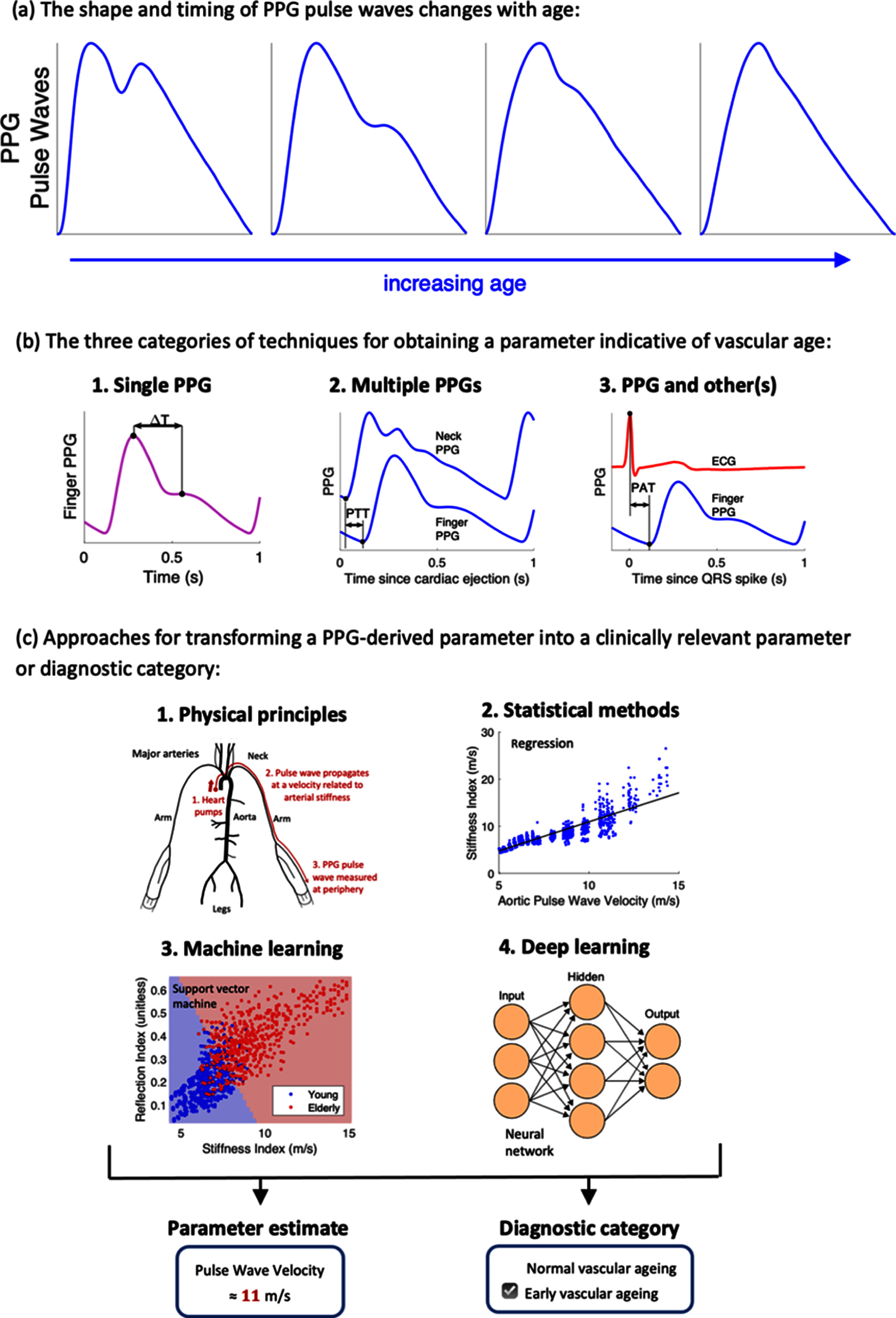
Assessing vascular ageing from contact photoplethysmography. *Definitions:* PPG—photoplethysmography; PTT—pulse transit time; PAT—pulse arrival time; ECG—electrocardiogram. Adapted from Charlton *et al* (2022b). CC BY 4.0. Adapted from Charlton *et al* (2018). © 2018 Institute of Physics and Engineering in Medicine. CC BY 3.0. Adapted from Charlton *et al* (2022a). CC BY 4.0.

Photoplethysmography is an optical measurement technique in which an arterial pulse wave signal known as the photoplethysmogram is acquired by shining light on the skin, and measuring the amount of light either transmitted through, or reflected from, the tissue. Photoplethysmograhy is widely used in clinical and consumer devices, such as fingertip pulse oximeters and smartwatches, for oxygen saturation and heart rate monitoring. The shape and timing of PPG pulse wave change with age (figure 7(a)), providing an opportunity to assess vascular age from the PPG (Charlton *et al* 2022b).

A range of techniques have been proposed to assess vascular age from the PPG, which broadly fall into three categories (figure 7(b)) (Charlton *et al* 2022b). Firstly, a single PPG signal can be used to obtain a parameter indicative of vascular age from the PW shape. The PPG signal can be measured by devices such as smartwatches, pulse oximeters, and non-contact cameras (using imaging PPG—iPPG). Secondly, two PPG signals can be used, such as finger and toe signals (Obeid *et al* 2017), to obtain pulse transit time (PTT) (the time taken for the PW to travel between two sites). Thirdly, pulse arrival time (the time delay between heart contraction and the arrival of a PW) can be obtained from a PPG signal and another signal which provides information on the time of heart contraction (such as the electrocardiogram, ECG). Once a parameter has been obtained, it can be transformed into either a clinically relevant parameter (such as PWV) or a diagnostic category (see figure 7(c)). Research to date provides evidence on (Charlton *et al* 2022b): (i) the level of agreement between PPG-derived parameters and reference indicators of vascular age; (ii) the repeatability and reproducibility of PPG parameters; and (iii) their potential clinical utility in peripheral arterial disease (PAD), diabetes, and cardiovascular risk assessment.

Future advances in PPG-based technologies could enable unobtrusive vascular ageing assessments across a wide section of society, utilising either contact PPG measurements from everyday devices such as smartwatches, or non-contact measurements from smartphones and tablets.

### Current and future challenges

There are several challenges to using PPG-based technologies for vascular ageing assessment, which are now described.

A key challenge is in obtaining accurate physiological information from PPG signals given that the PPG is influenced not only by vascular ageing but also a range of other factors. For instance, PPG PW shape is also influenced by heart rate, stroke volume, blood pressure (BP), and peripheral vascular compliance (Charlton *et al* 2019). Similarly, the timing of PPG pulse wave is influenced by BP. Therefore, it is a challenge to ensure PPG-based assessments of vascular ageing are not overly influenced by other CV properties. PW shape is also influenced by measurement factors such as (Charlton *et al* 2022a): anatomical site, contact vs. non-contact PPG, wavelength of light, transmission vs. reflectance PPG, contact pressure, temperature, and skin colour. The PPG signal also exhibits beat-to-beat variations, partly due to physiological variations (Elgendi 2012). All these influences should be accounted for when developing PPG-based techniques.

**Figure 8.**
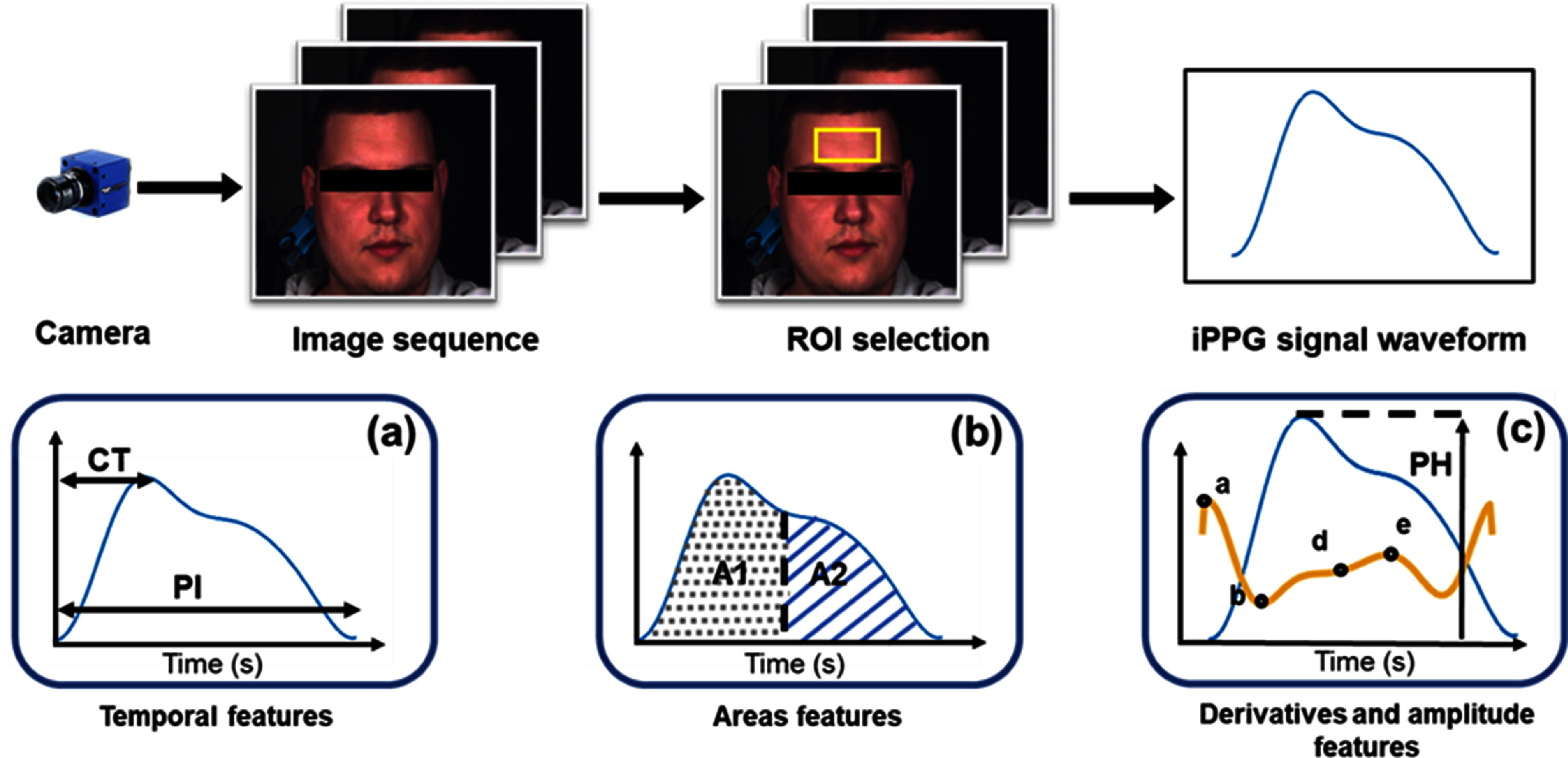
Imaging photoplethysmography (iPPG) waveform extraction and analysis: facial images are captured by a colour camera. Then the forehead is selected as region of interest (ROI). The raw iPPG signal is extracted from the ROI using spatial averaging. Before extracting the waveform, the signal is smoothed and filtered. Three waveform features can be computed: (a) temporal, (b) areas and (c) derivatives and amplitude features. These features are related to heart rate, blood pressure, vascular ageing and arterial stiffness. Adapted from Djeldjli *et al* (2021), Copyright (2021), with permission from Elsevier.

The challenge of obtaining accurate physiological information is all the greater for in the case of iPPG. iPPG consists of extracting a PPG signal from a video of the skin (usually the face) by analysing the pulsatile changes in light intensity (figure 8). Consequently, even involuntary movements such as the small movements associated with breathing and the heart beating can cause changes in facial position and orientation, causing changes in the ROI and its illumination (Djeldjli *et al* 2021). Once a stable signal is obtained, PW parameters can be derived (figure 8) and transformed into clinically relevant outputs using the same approach as for contact PPG signals (figure 7(c)). Similarly to contact PPG signals, features can be extracted from iPPG signals which are related to BP, arterial stiffness, and vascular ageing assessment (Djeldjli *et al* 2021). Consequently, there is the same challenge of separating information on vascular ageing from other factors influencing the iPPG signal. iPPG is an attractive alternative to contact PPG in cases of trauma, burns, and contagious diseases (Djeldjli *et al* 2021). In addition, it could be used with tablets and smartphones to conduct unobtrusive hemodynamic assessments. This provides great incentive for overcoming the additional challenges associated with iPPG.

A second challenge is presented by PPG signal quality (Zanelli *et al* 2021), as the PPG signal is highly susceptible to noise. Low quality signals can be caused by movement artefacts, poor sensor contact, low light intensity (which can be related to skin tone), reduced perfusion, and the use of iPPG as opposed to contact PPG. Potential options for handling low signal quality include: rejecting periods of low signal quality; using mathematical techniques to remove motion artefact from low-quality signals; and using simultaneous accelerometry signals for motion artefact removal.

A third challenge is in establishing reference values for PPG-derived parameters. Whilst some work has been conducted on establishing reference values (Choi and Park 2023), normal and pathological values of a wide range of parameters have not yet been comprehensively defined. Reference values could take into account physiological factors which are known to influence PPG pulse wave, such as BP, heart rate, age, and arterial stiffness (Elgendi 2012). It is also important to consider the impact of measurement factors, and to investigate whether different ranges are required for different measurement configurations (such as different anatomical sites).

A fourth challenge is in translating PPG-based techniques into clinical practice. As described in detail in section 11, techniques should be validated against reference measurements, the potential clinical utility of techniques should be investigated, and finally the cost-effectiveness of techniques should be assessed.

A fifth challenge is ensuring that PPG-based techniques perform well across subjects of all ethnicities. There are known differences between ethnicities in the patterns of vascular ageing (Schutte *et al* 2020) which should be taken into account in all vascular ageing assessments. In addition, since PPG involves interaction between light and the skin, PPG-based measurements can be affected by skin colour. This phenomenon is well-documented for pulse oximetry which uses PPG signals of two wavelengths (Al-Halawani *et al* 2023), and is now a key consideration for other PPG-based measurements such as wearable heart rate monitoring (Koerber *et al* 2023).

### Advances in science and technology to meet challenges

Novel publicly available datasets containing a range of PPG signals and reference physiological parameters would greatly aid work towards obtaining accurate physiological information from PPG signals. Currently, there are relatively few publicly available contact PPG datasets which are well-suited to vascular ageing research (Charlton *et al* 2022b), and similarly there are relatively few iPPG datasets (such as the BP4D+ dataset, a dataset designed for emotion recognition which contains movement (Zhang *et al* 2014)). Novel datasets could include PPG signals measured from subjects with different characteristics (spanning ranges of age, BP, and skin tone), using different measurement configurations (including anatomical site and contact vs non-contact measurements). Such datasets are of even greater value when they include reference vascular ageing parameters (such as PWV, BP, and age), and other physiological parameters (such as stroke volume). Advances in approaches for simulating PPG pulse wave recently led to the creation of a database of simulated PPG pulse wave containing reference measurements of a wide range of physiological parameters (Charlton *et al* 2019). Future work based on novel datasets could identify optimal measurement configurations and analysis techniques for obtaining parameters of vascular ageing which are not unduly influenced by other factors. The recent development of a technique to reconstruct a contact PPG signal from an iPPG signal (Bousefsaf *et al* 2021) provides opportunity to use the more plentiful contact PPG datasets when developing iPPG technologies (Djeldjli *et al* 2021).

A second research direction towards obtaining accurate physiological information is developing novel PPG analysis techniques, leveraging advances in AI techniques (Zanelli *et al* 2023). Deep learning models, such as convolutional neural networks, are widely employed in PPG analysis thanks to their capability of automatically extracting features from the signal avoiding the need for handcrafted features (Charlton *et al* 2022a). When working with deep learning techniques, particular attention must be paid to the amount of data required, the balance between classes inside the dataset (such as normal and pathological subjects), and the validation process that is used to correctly test the proposed model. An alternative approach is to combine our understanding of the physiology of the PPG PW with traditional signal processing techniques to obtain more refined measures of vascular ageing, which can be achieved by decomposing pulse wave into forward and backward waves (Lin *et al* 2022), or harnessing the additional information provided by multi-wavelength PPG which can be related to different depths of vasculature (Liu *et al* 2019).

The development of PPG sensor hardware may also help improve the accuracy of PPG-derived parameters. For instance, multi-wavelength PPG could provide insights into hemodynamics at different depths of the vasculature, and be used to assess arteriolar PTT (the time taken for the PW to travel through the arterioles) (Liu *et al* 2019). This may provide complementary assessments of vascular ageing to those typically conducted on large arteries (such as PWV), and may also help separate the influences of macro- and micro-vasculature on PPG signals.

The challenge presented by PPG signal quality is encountered in many other PPG applications (Charlton *et al* 2023), providing opportunity to translate work developed for other applications to vascular ageing assessment techniques, although methods to discriminate between high quality PPG—pathological or not, during physical activity or not—and low quality PPG, still need to be further explored. Further development of PPG sensor hardware may improve signal quality: combinations of different wavelengths could be more robust to movement and external noise; and potentially different wavelengths could be used based on the level of noise in the corresponding signals. Another approach is to develop strategies to obtain measurements during periods of high signal quality and steady CV state, such as while motionless or asleep.

The widespread and growing use of PPG sensors may help tackle the challenges of establishing normal ranges for PPG-derived parameters, and translating PPG-based vascular ageing assessments into clinical practice. Large studies are now being conducted using PPG measurements obtained from consumer devices, greatly aiding clinical translation (Perez *et al* 2019). Such studies typically use parameters derived from the PPG signal by the device. Therefore, to use this approach in the field of vascular ageing, consumer devices would either need to be equipped with an algorithm to assess vascular age, or to provide the PPG signal for retrospective analysis. This would then enable the development of reference ranges of parameters, and help assess clinical utility through linkage with electronic health record data.

The need to ensure equitable performance of PPG-based vascular ageing assessments between ethnicities means that studies of PPG-based devices techniques should include subjects with a sufficiently wide range of skin colours (potentially using objective skin pigmentation assessment (Al-Halawani *et al* 2023)). PPG device designers might consider the wavelength and intensity of light, and source-detector separation distance used to ensure equitable performance. Whilst photoplethysmograhy is widely used in wearable devices, it may not be the optimal sensing technology for wearable PW measurement, with several alternatives in development such as electrical bioimpedance (Kireev *et al* 2022). Alternatives to PPG are covered in another recent Roadmap (Charlton *et al* 2023), and those which do not use light sensing may help ensure performance is not affected by skin pigmentation.

### Concluding remarks

PPG is an attractive modality for vascular ageing assessment at scale, being non-invasive and widely incorporated into clinical and consumer devices. However, there are challenges in obtaining accurate physiological information from PPG signals, handling low-quality signals, establishing reference ranges of PPG-derived parameters, and translation into clinical practice. These challenges could be tackled through the development of publicly available PPG datasets, advanced PPG sensing hardware and analysis techniques, and harnessing the widespread use of PPG devices in daily life to conduct large-scale studies. The success of PPG-based techniques will be dependent on identifying clinical use cases which this technology is well suited to, as already demonstrated by the development of PPG-based technologies for detecting PAD.

### Acknowledgments

This work was supported by COST Action CA18216 VascAgeNet, supported by COST (European Cooperation in Science and Technology, www.cost.eu). P H C acknowledges funding from the British Heart Foundation (FS/20/20/34626).

### Conflicts of interest

S Zanelli collaborates with Axelife, a company that designs and develops devices for assessing vascular ageing. P Charlton has performed consultancy work for companies developing PPG sensors.

## 7. Circulating biomarkers

### Kristina Gopcevic^1^, Antonios Lazaridis^2^ and Eugenia Gkaliagkousi^2^

^1^ Faculty of Medicine, University of Belgrade, Belgrade, Serbia

^2^ Faculty of Medicine, Aristotle University of Thessaloniki, Thessaloniki, Greece

### Status

**Figure 9.**
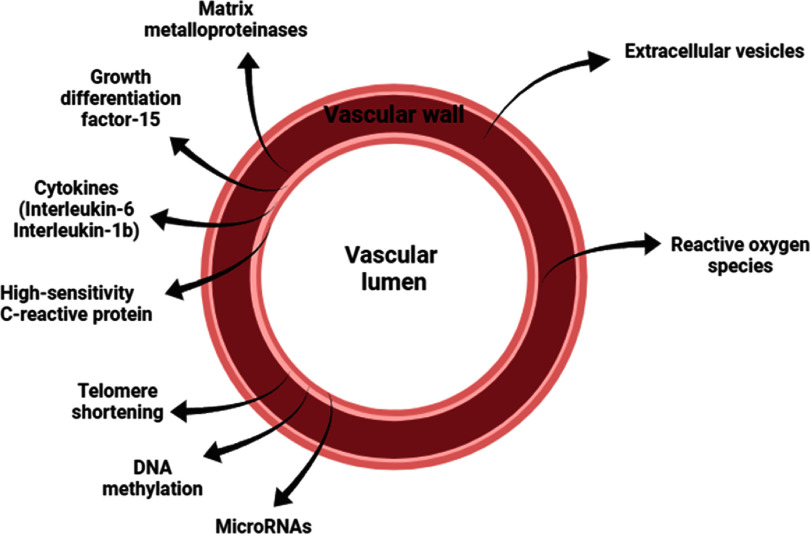
Some circulating biomarkers with the highest potential for early detection and diagnosis of cardiovascular disease and vascular ageing. Adapted from Gopcevic *et al* (2021). CC BY 4.0.

Over the past few decades, many circulating biomarker candidates identified in serum/plasma and tissue have shown potential in early detection and diagnosis of cardiovascular diseases (CVDs) and vascular aging (VA), as illustrated in figure 9. However, only a few have been carefully evaluated in a large cohort of samples for further validation of their utility (Gopcevic *et al* 2021). The lack of a rapid, convenient and reliable format of testing for a promising biomarker in an appropriate specimen type presents an obvious bottleneck for successful translation of biomarker candidates in the clinic. The most promising are: superoxide-dismutase (SOD), matrix metalloproteinases (MMPs), high sensitivity C reactive protein (hsCRP), interleukin-6 (IL-6) and interleukin-1b (IL-1b), growth differentiation factor-15 (GDF-15), micro ribonucleic acids (miRNAs), DNA methylation, extracellular vesicles (EVs) (Gkaliagkousi *et al* 2021, Lazaridis *et al* 2022, Femmino *et al* 2023) and telomere shortening. Current measurement techniques for MMPs activity include: zymography using different substrates and immunoassays. Reactive oxygen and nitrogen species can be measured directly—concentration of reactive species produced or indirectly—targeting the molecules of the antioxidant system changed in response to increased redox stress, such as activity of SOD. The hsCRP, IL-6, IL-1b, and GDF-15 markers can all be measured with readily available quantitative immunoassays. Concerning profiling of miRNAs, the reverse transcription quantitative real-time polymerase chain reaction (PCR) is the most widely sensitive method. EVs are potent mediators of inflammation, coagulation, oxidative stress and immune system modulation, therefore holding a significant mechanistic role in the pathophysiology of CVD. To determine the EVs origin, structure, and size, immunoblotting of specific proteins, flow cytometry, transmission electron microscopy, and nanoparticle tracking analysis are used. DNA methylation is predominantly measured in DNA extracted from various tissues, commonly whole blood. Bisulfite sequencing is considered to be the reference method for single-base resolution measurement of DNA methylation levels and PCR-based techniques are routinely used to study DNA methylation on a gene-specific basis, after bisulfite treatment. Currently also, genome-wide analyses such as massive parallel sequencing and microarray-based platforms have been developed for methylome profiling and identification of differentially methylated regions at a genome-wide scale. Finally, telomere shortening is measured in DNA extracted from peripheral blood leukocytes. There are three main methods for measuring telomere length: (a) southern blot, (b) quantitative PCR, and (c) fluorescent in situ hybridization-based methods. Overall, Southern blot remains the most accurate method for precise measurement of telomere length while Quantitative polymerase chain reaction is the most employed method for large population-based studies.

### Current and future challenges

The current knowledge of MMPs provides a perception of their multifunctional implication in, highlighting their relevance as potential therapeutic options. At least 27 MMPs can be determined by bioassays, zymography, western blotting, ELISA. All methods have many disadvantages: low specificity, limits of detection, broad spectrum of substrate specificity. It has been recently proposed use of different kind of biosensors (Kirchhain *et al* 2019) which allowed detection of MMPs using MALDI-TOF technique. Currently it is difficult to accurately measure and assess RONS levels, owing to their extremely short half-lives. The measurement of SOD is one of most accepted means of measuring RONS activity owing to their levels reflecting their activity catalysing the dismutation of the strong superoxide radical (O^2−^) (Griendling *et al* 2016). Among many other methods, SOD activity is measured indirectly as a concentration of water-soluble formazan dye which can be detected by the increase in absorbance at 450 nm. IL-6 is considered one of the most representative biomarkers of vascular ageing, closely associated with its chronic inflammatory microenvironment. Understanding the biology of IL-6 receptor and its signalling axis remains a major challenge due to the significant anti-inflammatory potential observed with targeted anti-IL6 monoclonal antibodies. However, the existing therapeutic antibodies available are few due to various biological and economic limitations (Gopcevic *et al* 2021). At the same time, the role of IL-1b as a cytokine-based therapy for the secondary prevention of atherosclerotic events and the specific mechanisms driving its beneficial effect on CV events reduction, remain to be elucidated (Ridker *et al* 2017). hsCRP is an inflammatory biomarker strongly linked to vascular wall biology and the prediction of future CV events (Mozos *et al* 2019). However, being an acute phase reactant, elevated hsCRP levels currently lack specificity for CV disease. Therefore, it is still an unresolved issue whether a causal relationship between hsCRP and CV disease exists and whether hsCRP measurement could provide clinically meaningful incremental predictive value in risk prediction and reclassification (2). Highly elevated GDF-15 levels have been mostly linked to pathological conditions including inflammation, myocardial ischemia, and notably cancer. With age, only a moderate increase in GDF-15 blood levels is observed. Hence, there is a clear need to better understand several GDF-15 mediated effects (including its physiological role in ageing). Particular emphasis should be placed on its immunomodulatory potential (Brábek *et al* 2020). At present, the biggest challenge concerning all miRNA profiling approaches is the lack of a consensus normalization method. This is due to the individual nature and composition of miRNAs and the resulting data distribution of profiling experiments. In addition, data derived by assays of circulating miRNAs suffer from a lack of validated, universal, reference miRNAs which can firmly detected in the blood (Vlachopoulos *et al* 2015). Current methods for isolation, identification and quantification of EVs need further standardisation. Their application in (bio)medical sciences requires an accurate assessment of their biochemical and physical properties. Several aspects should be considered when translating DNA methylation markers into clinical practice. These include (i) identification of the best genomic regions since methylation takes place over large and poorly defined regions, (ii) address of the low-level methylation phenomenon in order to set a cut-off point between pathologically significant and insignificant methylation, (iii) pre-analytical processing techniques, and finally (iv) standardisation of data analysis to define which method has the highest diagnostic potential (Marabita *et al* 2016, Wischhusen *et al* 2020). Finally, telomere length (TL) measurement suffers from a substantial inter-individual variability of leukocyte TL across multiple populations and various tissue types, and it is questionable whether findings in circulating leukocytes can be generalized to other tissues. Importantly, most TL measurement techniques produce a measure of the average TL, which is not representative of the mechanisms linking TL to ageing (Wagner 2022).

### Advances in science and technology to meet challenges

A better understanding of which MMP is involved (and when) in a specific pathological process could lead to the potential application of MMPs as biomarkers for early diagnostic approaches and for estimating the prognosis of the disease. Over the last decade, the growing interest for MMPs has pushed the development of a variety of devices based on different recognition methods and signal transductions aimed at measuring MMP enzymatic activity. Modification of amino acid residues allowed detection of some MMPs using biosensors and proteomic based techniques (Kirchhain *et al* 2019). Many methods have been devised to measure RONS with various levels of sensitivity and accuracy. These include chemical assays for, H_2_O_2_, or •OH generation, direct chemiluminescent assays, fluorescence detection, and either direct or spin trapping-based electron paramagnetic resonance (EPR) spectroscopy. Only EPR enables the direct detection of free radicals, but other assays can be informative if used with proper controls. Apart from the established role of hsCRP as a biomarker surrogate for predicting the risk of vascular events in primary CV prevention, further advances have provided direct evidence that reducing hsCRP can reduce rates of recurrent CV events, therefore paving the way for the effective identification and management of residual inflammatory risk with targeted anti-inflammatory drugs (Jain *et al* 2013). Substantial advances have been made in translating the biology of IL-6 into the development of several effective monoclonal agents that target the IL-6 signalling pathway, thus showing promising results (Vaiserman and Krasnienkov 2021). Similarly, several effective inhibitors of IL-1b signalling have been clinically approved, therefore expanding the IL-1 signal-targets for the treatment of a broad spectrum of diseases beyond those with inflammatory substrate (Kaneko *et al* 2019). GDF-15 has been widely explored as a biomarker for disease prognosis. More importantly, the prognostic impact of GDF-15 in healthy individuals has been recently clarified by providing continuous reference values for GDF-15 in apparently healthy older adults and showing a significant association between reference values and risk of all-cause mortality (Ridker *et al* 2017). Considering the special features of miRNAs such as small size, low content, and high sequence similarity, a great effort has been put into the improvement and development of many sensitive nucleic acids’ amplification techniques along with the conventional miRNA detection methods, allowing for an improved detection accuracy of miRNAs (Ouyang *et al* 2019, Choy *et al* 2020). Several studies trying to unravel the biological function of EVs or focusing on biomarker discovery also used high-resolution molecular profiling of EV content (protein, RNA and lipids) using proteomics, genomics and lipidomics approaches (Hartjes *et al* 2019). Future progress in this field will certainly lead to technology for rapid and reliable quantification and characterization of EVs, which will provide reliable and reproducible data (Hartjes *et al* 2019). Over the past decade, there has been a revolution in DNA methylation profiling technologies moving from analyses restricted to specific loci towards microarrays and next generation sequencing platforms that allow the analysis on a genome-scale and the characterization of entire methylomes of the cells at single-base-pair resolution. With these advances, the numbers of candidate DNA methylation markers have significantly increased (Ridker *et al* 2017). Many innovative technological approaches have been developed to measure TL including FISH-based methods and the telomere shortest length assay. These methods provide highly accurate and reliable results and allow detection of even subtle changes in TLs. More importantly, they can identify the shortest telomeres (<3 kbp) which are critical for cell viability, thus providing new opportunities in assessing biological age (Lai *et al* 2018).

### Concluding remarks

Many of assay developed to measure circulating biomarkers are used inappropriately or without due experimental diligence, resulting in potential inaccuracies and artefacts. Development of new analytical methodologies should focus on direct measurement of circulating biomarkers, using assays that are user-friendly, practical, and of clinical utility. Because the ideal biomarker has not yet been identified, it is prudent to measure multiple independent biomarkers.

### Acknowledgments

This manuscript is based upon work from the European COST ACTION CA18216 ‘Network for Research in Vascular Aging’ supported by COST (European Cooperation in Science and Technology, www.cost.eu). The authors thank Ioana Mozos for feedback on this section.

## 8. Risk scores

### János Nemcsik

Department of Family Medicine, Semmelweis University, Budapest, Hungary

### Status

The evaluation of the exact vascular age expressed in years or the identification of subjects with EVA based on already developed or newly constructed risk scores can help in the recognition of high CV risk patients. Additionally, it also can improve CV risk communication. For example, using the Systematic COronary Risk Evaluation (SCORE)-based vascular age calculation method (Cuende *et al* 2010) the arteries of a 50 year old female smoker with a systolic BP of 180 mmHg and total cholesterol level of 8 mmol l^−1^ are 70 years old. The fact that her arteries are 20 years older than her chronological age could be more convincing to follow the instructions of her physician compared with the communication of her SCORE-based 4% risk for CV mortality within the next 10 years.

The first vascular age risk score-based method was published in 2008 by D’Agostino *et al*, based on the Framingham risk score (FRS), which calculates the 10 year risk of CV events and CV mortality (2008). The FRS-based vascular age is the hypothetical age of a subject with the same CV risk (as assessed by FRS points) but with the remaining risk factors within normal ranges. FRS calculation is different for men and women and it considers age, total and high-density lipoprotein cholesterol, systolic BP, treatment for hypertension, smoking and diabetes status (D’Agostino *et al* 2008).

In 2010 Cuende *et al* published the SCORE-based vascular age calculation method (2010). As the FRS-based vascular age, it also calculates the hypothetical age of a subject with the same CV risk but with the remaining risk factors within normal ranges, meaning that CV risk is only based on age and sex. This method considers fewer variables compared with the FRS-based method, namely age, sex, total cholesterol, systolic BP and smoking status (Cuende *et al* 2010). Although in 2021 the new SCORE2 was introduced (Score2 Working Group and ESC Cardiovascular Risk Collaboration 2021), adjusted vascular age calculation method is not published yet.

There are also newly constructed risk scores, which do not provide an exact number of the age of the vasculature, but only aim to identify patients with EVA. These calculators are developed based on the comparison with the reference values of cfPWV. The EVA ambulatory score requires ambulatory BP monitoring and considers 24 h systolic and diastolic BP and heart rate, age, sex, body mass index, diabetes mellitus (yes/no) and estimated glomerular filtration rate (Antza *et al* 2018). Recently, in patients with metabolic syndrome a score was also developed considering age, sex, weight, uric acid levels, history of type 2 diabetes mellitus and clinical markers of insulin resistance (IR) such as homeostatic model assessment of IR and clinical IR (Nedogoda *et al* 2021).

### Current and future challenges

Although some methods are available to calculate vascular age and to screen patients with EVA, it is not obvious that these different methodologies identify the same subjects at risk. On the contrary, recent studies found marked differences.

**Figure 10.**
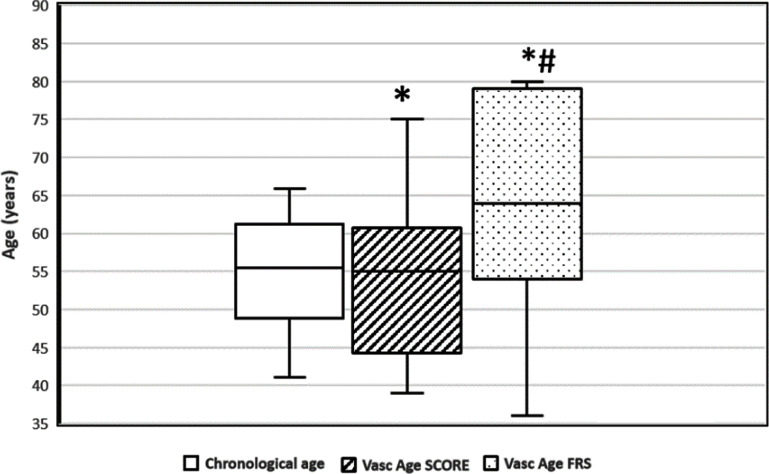
The chronological age, the vascular age calculated based on the Systematic COronary Risk Evaluation (Vasc Age SCORE) and the vascular age calculated based on Framingham risk score (Vasc Age FRS) of the study population. Data are presented as median (minimal and maximal values in error bars). *p < 0.05 compared with Chronological Age; #*p* < 0.05 compared with Vasc Age SCORE. Adapted from Gyöngyösi *et al* (2021). CC BY 4.0.

Gyöngyösi *et al* demonstrated on 172 subjects who participated in a CV screening program, that vascular age based on FRS and SCORE can provide markedly different results (figure 10) (2021).

The observed difference was a consequence of the differences of the two methods. Since the FRS considers also hypertension and diabetes, the estimated FRS vascular age appears to be higher when compared to the SCORE vascular age for the same subject. In the absence of pathologies the two methods gave similar results (Gyöngyösi *et al* 2021). In the same paper it was also demonstrated that the comparison of the proportion of subjects with elevated vascular age identified with FRS, SCORE or cfPWV (FRS+, SCORE+, PWV+, respectively) differed from each other. The 84% of PWV+ and 85.4% of SCORE+ patients were found to be FRS+ as well. However, less acceptable overlap was found between PWV+ and SCORE+ subjects, as only 30.9% of SCORE+ patients was PWV+ as well, and only 24.6% of the PWV+ subjects had elevated vascular age based on SCORE (Gyöngyösi *et al* 2021).

These results were confirmed by the study of Vecsey-Nagy *et al* as well, as among 241 patients with stable angina referred to coronary CT angiography (CTA) in subjects with treated hypertension and diabetes, FRS-based method resulted higher vascular age values compared with the SCORE-based method (2022). In the same study, coronary artery calcium score (CACS)-based vascular age calculation method identified a lower proportion of patients with elevated vascular age compared with the FRS and SCORE-methods (Vecsey-Nagy *et al* 2022).

Finally, the difference between FRS and SCORE-based vascular age calculation methods was confirmed by Kozakova *et al* as well, in 528 middle-aged individuals (2022) and recently in a population-based cohort including 99 231 subjects (Gyöngyösi *et al* 2023).

Based on these studies it can be concluded that SCORE-based vascular age calculation methods should be kept for apparently healthy subjects. Subjects who already have hypertension or diabetes would probably benefit more from FRS-based vascular age-guided preventive strategies. However, prospective studies are required to clarify the success of different risk score-based vascular age-derived CV preventional strategies.

### Advances in science and technology to meet challenges

There are still some open questions in this field which need to be clarified in the future. Results of the above mentioned comparative studies (Gyöngyösi *et al* 2021, Vecsey-Nagy *et al* 2022) suggest, that risk score-based vascular age calculation methods identify a higher proportion of patients with EVA compared with measurement-based methods using e.g. cfPWV or CACS. The future approach might be a composite vascular age calculating method based on both a measurement and components of risk scores. However, prospective studies are required to clarify this question as well. Optimally these studies should be planned for patient randomization of the application of preventive strategies based on the detection of EVA by different methods. As such studies are costly and time-consuming, alternatively retrospective cohort studies could also provide useful data. Those cohorts should be analysed retrospectively in which both measurements of vascular ageing biomarkers were performed and the variables of the risk score-based vascular ageing calculation methods are also available.

Another important task which requires consensus is the definition of EVA based on the calculations e.g. 1, 2 or 5 etc years above chronological age? In the study of Antza *et al* the constructed score for the identification of patients with EVA was compared with cfPWV above the age-adjusted value (2018). In contrast, Nedogoda *et al* defined EVA with values of cfPWV exceeding expected for chronological age values by at least 2 standard deviations (2021), while Nilsson *et al* defined EVA subjects in the highest 10% of the standardised cfPWV distribution, adjusted for age intervals (2018).

Finally, the risk score-based vascular age calculation techniques should be validated in different geographical regions and also besides different comorbidities which accelerate vascular ageing e.g. chronic kidney disease or diabetes.

### Concluding remarks

Risk score-based vascular age calculation provides a simple possibility to identify patients with EVA and to improve CV risk communication. However, there are differences between the methods, e.g. they provide different values of vascular age and they sometimes identify different patients with EVA, which are needed to be resolved. Application of the different methods on proper patient populations or their combination with measurement techniques can provide solution in the future. Additionally, prospective studies are required to clarify the strength of preventive strategies based on different vascular age calculation methods.

## Development pipeline

## 9. Development using synthetic Pulse Wave data

### Jordi Alastruey

Department of Biomedical Engineering, School of Biomedical Engineering and Imaging Sciences, King’s College London, London SE1 7EU, United Kingdom

### Status

**Figure 11.**
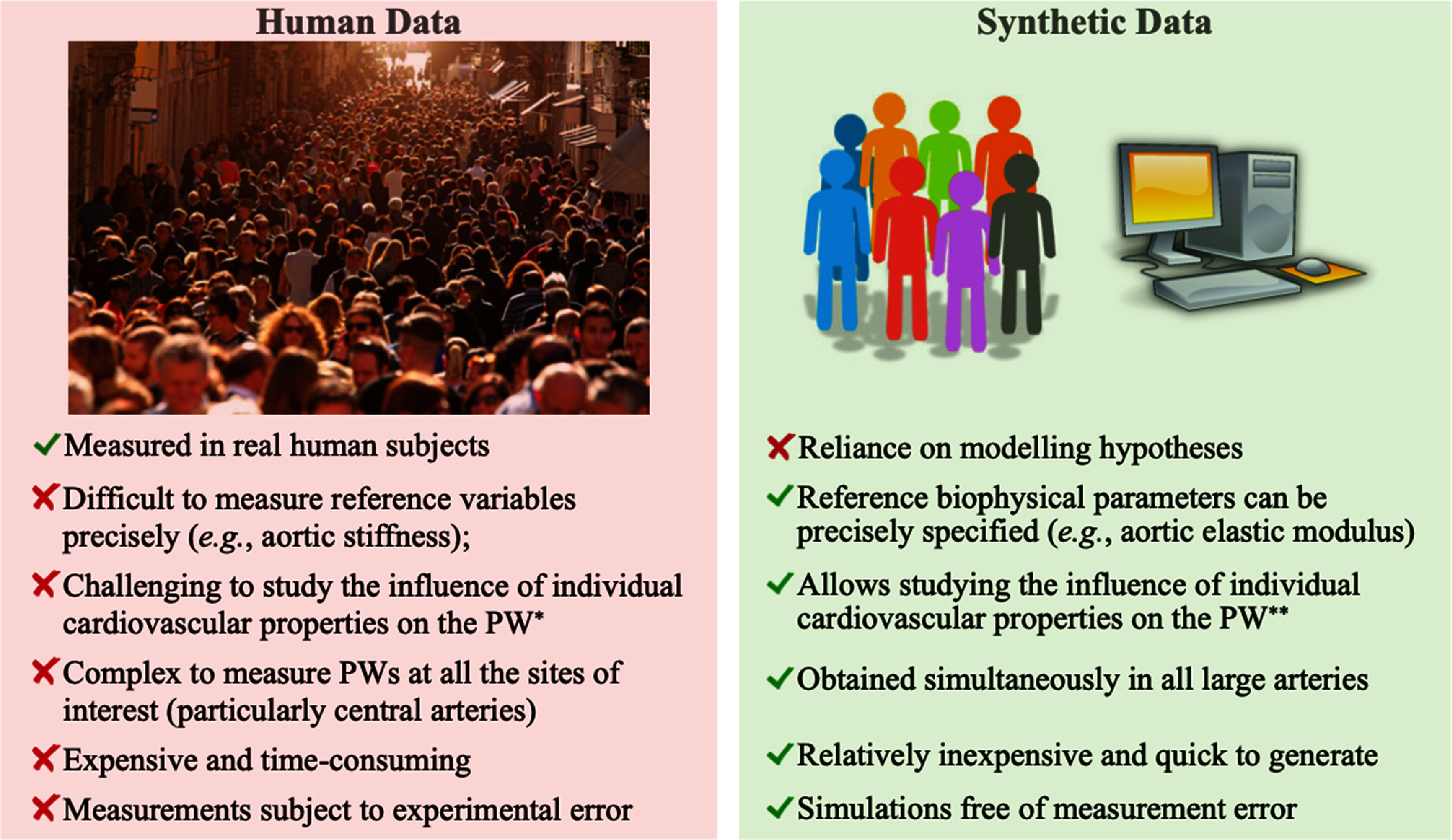
(Left) Current challenges using *in vivo* human data to develop technologies to assess vascular ageing from pulse wave (PW) signals. (Right) Advantages offered by synthetic data to overcome these challenges. * Other properties may change concurrently. ** Individual properties can be altered independently of each other.

Pulse wave (PW) signals, such as arterial blood pressure (BP) and photoplethysmographic (PPG) waves, are produced by the beating heart interacting with flexible arteries. vascular ageing changes the mechanical and structural properties of the vascular wall that determine the morphology of PW signals (Climie *et al* 2023), offering the opportunity to assess vascular aging (VA) from PW signals. The development and testing of suitable PW analysis techniques require large PW datasets, but acquiring them is often a complex task subject to several problems (figure 11, left). To facilitate this process, datasets of synthetic (simulated) pulse wave representative of data samples of real subjects have been created under a wide range of CV conditions. They complement human data and have many advantages (figure 11, right), despite the obvious limitation of relying on modelling hypotheses.

**Figure 12.**
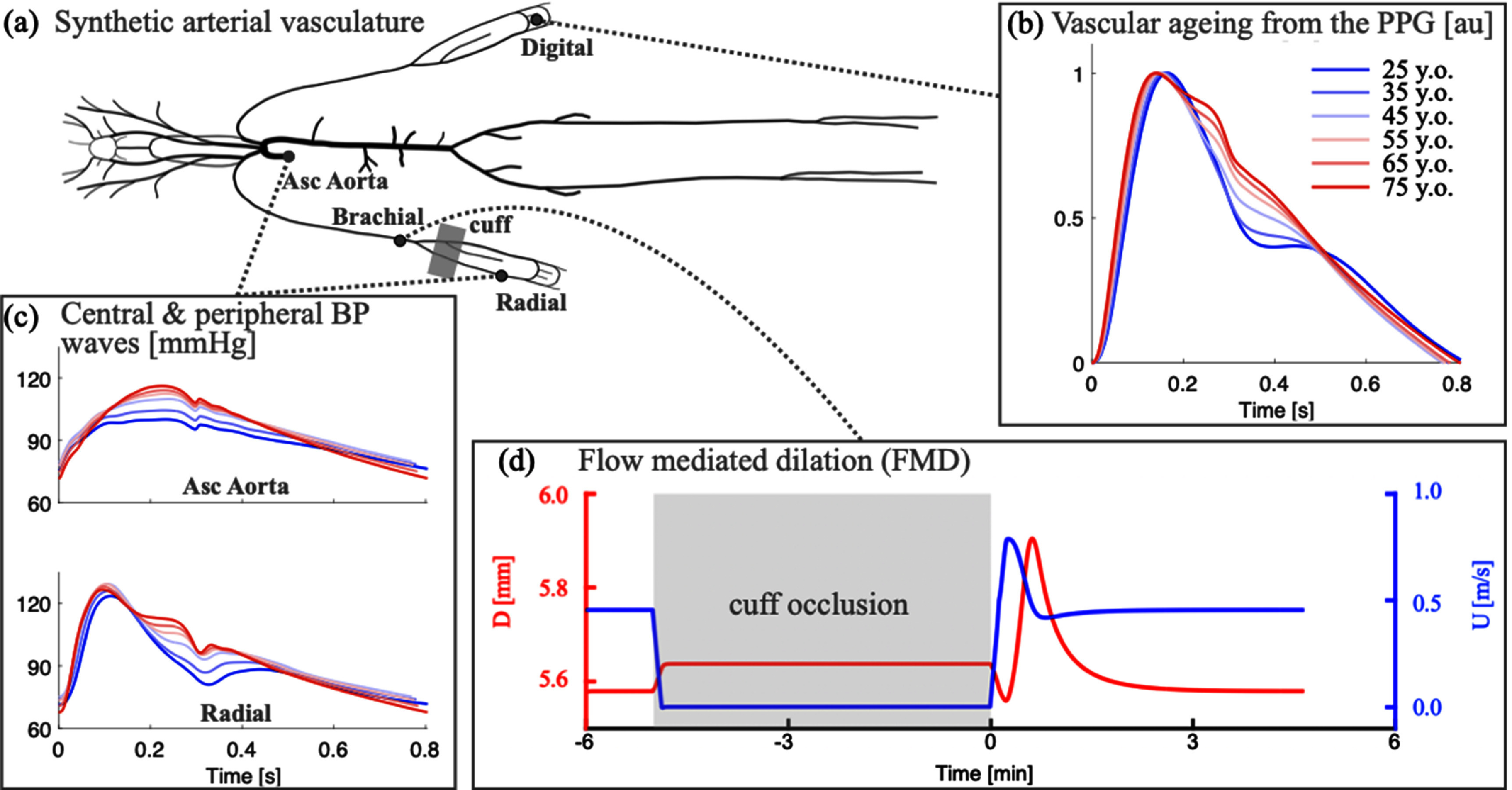
Examples of synthetic PW signals. (a) Arterial network used to simulate the signals. (b) The PPG at the finger changes with ageing, allowing the study of PPG-derived vascular ageing indices. (c) Central and peripheral blood pressure (BP) waves for evaluating the estimation of the former from the latter, across different age groups. Adapted from Charlton *et al* (2019). CC BY 4.0. (d) Cardiac cycle-averaged diameter (*D*) and flow velocity (*U*) during flow-mediated dilation (FMD). Adapted from Jin *et al* (2020). CC BY 4.0.

Datasets comprising thousands of virtual subjects have been generated using blood flow modelling with a ‘population-specific’ approach. In this modelling strategy, data from various real subject populations are amalgamated to calibrate model input parameters and validate simulated pulse wave (Willemet *et al* 2015, Charlton *et al* 2019, Bikia *et al* 2020, Jones *et al* 2021). For each virtual subject, different types of PW signals (pressure, flow, luminal area/diameter, PPG) are available simultaneously at common measurement sites in large arteries (figure 12), together with precise values of all cardiac and vascular biophysical properties that produced these signals; e.g. geometrical and material properties of the arterial network. Consequently, these datasets provide an opportunity to benchmark PW analysis techniques for vascular ageing assessment; e.g. the estimation of arterial stiffness (Willemet *et al* 2015, Charlton *et al* 2019, Jin *et al* 2021), central BP (Vennin *et al* 2017, Bikia *et al* 2020), and identification of stenoses and aneurysms (Jones *et al* 2021, Wang *et al* 2021) from non-invasive pulse wave. Various techniques can be compared; e.g. different foot-to-foot methods (Willemet *et al* 2015) or ML models (Jin *et al* 2021) for estimating PWV (an index of arterial stiffness). Synthetic pulse wave also make it possible to identify potential shortcomings of existing technologies; e.g. assessing endothelial function using flow-mediated dilation (Jin *et al* 2020). Furthermore, the effects of sampling rate and uncertainty in the PW data can be investigated (Jin *et al* 2021, Wang *et al* 2021, Hong *et al* 2023). Since synthetic pulse wave are generated using physics-based models, they facilitate the evaluation of haemodynamics; e.g. the identification of cardiac/vascular determinants of PPG-derived indices of arterial stiffness (Charlton *et al* 2019, Hong *et al* 2023). As synthetic PW modelling advances, combined with ML and the proliferation of PW wearable sensors, new opportunities for vascular ageing assessment in the clinic and daily life will emerge.

### Current and future challenges

*In silico* studies, involving synthetic PW datasets, have been used to confirm findings from *in vivo* studies (Willemet *et al* 2015, Vennin *et al* 2017, Jin *et al* 2021, Wehenkel *et al* 2023), yet they lag behind other areas of biomedical engineering where simulated data have successfully replicated clinical trials (Sarrami-Foroushani *et al* 2021). In the future, synthetic PW datasets could potentially reduce, refine, and even replace human and animal trials in developing reliable techniques for vascular ageing assessment in the clinic and daily life. This will require addressing the following challenges.

Synthetic PW datasets have been used to train and test ML models under a wide range of CV conditions and assist with their interpretability (Bikia *et al* 2020, Jin *et al* 2021, Jones *et al* 2021, Wang *et al* 2021, Wehenkel *et al* 2023). For instance, synthetic data has demonstrated the feasibility of assessing arterial stiffening from a single PW (e.g. measured on the arm) using PWA (Charlton *et al* 2019, Hong *et al* 2023) or machine-learning-based PW models (Jin *et al* 2021, Wehenkel *et al* 2023). Nevertheless, it remains unproven whether models trained with synthetic pulse wave will provide accurate results when tested *in vivo*. Achieving this is likely to require improving current computational blood flow models for generating synthetic PW datasets. Existing models typically provide pulse wave in supine conditions over seconds, and lack important physiological feedback and control mechanisms to simulate beat-to-beat variations under a wide range of dynamic CV conditions over minutes/hours (e.g. exercise and postural changes) and over years (e.g. sex-specific growth patterns and disease progression). Furthermore, current datasets lack representation of virtual subjects of both sexes, various ethnicities, and all age ranges from infants to adults, under physiological and pathological conditions affecting vascular ageing. Consequently, a technique that works on synthetic PW datasets is not guaranteed to be applicable in an *in vivo* setting. Synthetic data should also play a key role in developing algorithms based on AI for the early prediction of an individual’s vascular ageing trajectory, from birth to old age, through automated interpretation of PW signals acquired by non-invasive wearable devices in daily life. These algorithms could improve the prevention of CVDs associated with vascular ageing.

### Advances in science and technology to meet challenges

The creation of synthetic PW datasets addressing the above-mentioned challenges could still be approached using a ‘population-specific’ modelling strategy. However, this will necessitate computational blood flow models where state-of-the-art governing equations are coupled with additional equations describing short- and long- term dynamic aspects affecting PW signals. These equations should consider physiological feedback and control mechanisms, as well as the evolution of the arterial wall components (e.g. elastin and collagen) throughout life and account for the broad spectrum of alterations produced by vascular ageing that affect functional and structural components of the arterial wall (Climie *et al* 2023). Furthermore, models should quantify how uncertainty in input parameters and artefacts (such as motion) translate into variability in synthetic pulse wave.

The development of AI-based algorithms for early prediction of an individual’s vascular ageing trajectory should benefit from having digital twins that improve at forecasting vascular ageing as they are fed, during life, with the individual’s *in vivo* PW signals and other basic clinical data such as age, sex, ethnicity, and body dimensions. Morphing/scaling methods will be needed to automatically generate the arterial network of an individual’s digital twin to match their changing body dimensions throughout life. This may be achieved using large datasets (e.g. UK Biobank) containing medical images of the arterial network of thousands of individuals together with basic clinical data and body dimensions. The development and training of these new technologies will likely require the combination of *in vivo* and synthetic PW data to overcome the need to acquire large sets of *in vivo* data measurements over a lifetime. Final testing will have to involve longitudinal studies in large populations of real subjects.

As new synthetic PW datasets are being created, they should be made freely available to the scientific, clinical, medical device, and digital health communities to support further research, and promote and facilitate their use in the development of technologies to assess vascular ageing. This can be achieved by creating open access online repositories (e.g. PhysioNet) containing the datasets and providing guidelines for their use, accompanied by peer-reviewed articles describing their capabilities and limitations. Engagement with all the above stakeholders will also be beneficial to receive valuable feedback on how to improve datasets and ensure the adoption of techniques for vascular ageing assessment developed with synthetic data, which will eventually require regulatory approval.

### Concluding remarks

Synthetic PW data are created through ‘population-specific’ modelling and offer a readily available and cost-effective approach for the development and pre-clinical testing of technologies that assess vascular ageing under a wide range of CV conditions. Successfully addressing the challenges discussed in this report could provide a robust *in silico* framework for the development of trustworthy techniques (with high accuracy and low uncertainty) for vascular ageing assessment in the clinic and daily life that are ready for implementation in real subjects. These techniques may encompass AI-based algorithms and digital twins capable of forecasting an individual’s vascular ageing trajectory throughout life through the automated interpretation of PW signals acquired noninvasively by wearable devices.

### Acknowledgments

This work was supported in part by the British Heart Foundation under Grant (PG/15/104/31913), in part by the Wellcome EPSRC Centre for Medical Engineering at King’s College London under Grant (WT 203148/Z/16/Z), and in part by the Department of Health and Social Care (DHSC) through the National Institute for Health and Care Research (NIHR) MedTech Co-operative award for Cardiovascular Diseases to Guy’s & St Thomas’ NHS Foundation Trust in partnership with King’s College London (MIC-2016-019).

## 10. *In-vitro* and *ex-vivo* models: importance and challenges

### Ana Belen Amado Rey^1^, Ana Carolina Gonçalves Seabra^1^ and Thomas Stieglitz^1,2^

^1^ Laboratory for Biomedical Microtechnology, Department of Microsystems Engineering—IMTEK, IMBIT—NeuroProbes, BrainLinks-BrainTools Center, University of Freiburg, Freiburg, Germany

^2^ Bernstein Center Freiburg, University of Freiburg, Freiburg, Germany

### Status

The CV system (CVS) is intricately interdependent on complex biomechanics variables such as geometry and stiffness of the vessels, as well as blood viscosity. These variables are often difficult to study and measure directly within the body, which would need an invasive ***in-vivo* study**. Furthermore, *in-vivo* experiments need thorough preparation and approval from ethical committees, which is time and cost-consuming. The **need to model and study a living organism through a non-invasive approach**, where conditions can be designed for specific case studies, from low to high model specificity, is made possible through *in-vitro* and *ex-vivo* methods and models. ***In-vitro* studies** refer to experiments either with components of an organism that have been isolated, e.g. cells, or with technical models as benchtop experiments that aim to recreate *in-vivo* conditions through non-biological materials. In contrast, ***ex-vivo* studies** are performed with tissue or organs, e.g. arteries, that have been extracted from an organism post mortem. The *ex-vivo* studies take place in an artificial environment with minimum alteration of natural conditions. A key example is given for each model:

• ***In-vitro***: Wisotzki *et al* (2022) developed an *in-vitro* hardware simulator of the CVS to generate physiologic flow conditions across a wide range of values to create a benchmark dataset of healthy and pathological conditions. The system consists of silicone tubes as mimicked arteries and a water-glycerine mixture as blood, with a ventricular-assisted device generating parametric flow.
• ***Ex-vivo***: Cooper *et al* (2021) developed an *ex-vivo* vascular bioreactor system to assess acute arterial drug retention based on a porcine carotid artery under physiological flow.

**Figure 13.**
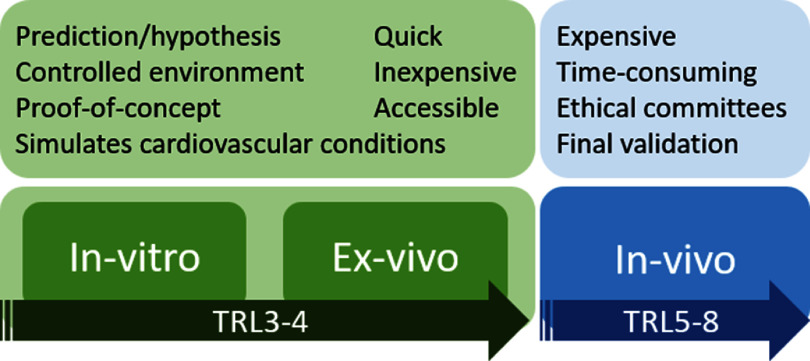
Novel technology evaluation methods’ characteristics with associated TRL.

In addition, these methods **allow the validation of vascular health device prototypes at different stages of product development** and under different CV conditions, ensuring strong reliability for *in-vivo* applications. Validation of novel technologies is done through the comparison of hemodynamic parameters extracted with the novel method and a reference standard (e.g. values from a commercially validated device) at the CVS model. Namely, Gonçalves Seabra *et al* (2022) evaluated *ex-vivo* a novel US sensor for BP measurement (comparison to a commercial pressure sensor). The technology readiness level (TRL) scale is used to access the maturity level of a technology (EURAXESS 2020). *In-vitro* and *ex-vivo* studies are essential for a novel technology to move up the TRL scale (see figure 13), from initial (TRL3) to demonstrated proof-of-concept (TRL4) and towards clinical trials (TRL5-8).

Ideally, *in-vitro* and *ex-vivo* models emulate healthy/pathological parameters, such as PWV, the reference standard for arterial stiffness assessment and indicator of vascular age. Thus, **patient-specific, reliable and accurate *in-vitro* and *ex-vivo* CVS models offer enormous potential in the simulation of various (patho-) physiological conditions in the arteries, blood and tissue**. Furthermore, these models **aid the parameter verification and validation of medical devices prototypes** (e.g. ultrasonic, tonometric, oscillometric, optical devices) before performing *in-vivo* measurements. However, the CVS is a sophisticated structure that is indeed challenging to emulate.

### Current and future challenges

An accurate determination of local PWV and stiffness indexes, concretely the acquisition of Peterson’s elastic modulus in patients, requires high levels of expertise (Ruesink *et al* 2018). Simplified models based on *in-vitro* and *ex-vivo* CVS, as well as long-lasting and reusable phantoms, improve the user experience and help to develop, validate, and optimize emerging devices to monitor vascular ageing. Thus, the costs and time required for the *in-vivo* validation in humans may be reduced when the user experience and the performance of the PWV device are optimized *a priori. In-vitro* and *ex-vivo* models can represent hemodynamics with high accuracy. As an example, Joseph *et al* (2018) developed a carotid flow phantom, in which acquisitions of the differential pressure (Δ*P*) with a new cuffless multimodal probe were compared to the reference Δ*P* values from a catheter. The reference differential pressure from the catheter showed a strong agreement with the estimated differential pressure from the cuffless multimodal probe (Pearson correlation of 0.93). However, several challenges need to be overcome to ensure *in-vitro* and *ex-vivo* models are accurately realistic and produce results which are replicated in *in-vivo* cases. The most important challenges are as follows:

• **Fabrication of realistic models that also emulate pathophysiological conditions.** For healthy subjects, the local PWV and cfPWV are strongly correlated (Simova *et al* 2016). Thus, even simple *ex-vivo* models to assess local PWV can be applied. Yet, for patients with EVA or other CVDs, no strong correlation exists. CVS models with changes in arterial stiffness, resistance, and compliance need to be developed and validated.
• **Accurate simulation of the heart and arteries.** *In-vitro* models emulate the heart using peristaltic pumps and mechanical valves to simulate the opening and closing of the aortic valve. Low blood flows (i.e. less than 15 ml min^−1^) are challenging to simulate with these pumps and increasing blood flow introduces distortion, noise and bubbles in the flow that can produce false measurements. The development of artificial arteries with several stiffnesses, thicknesses and patient-specific geometries is also a complex challenge.
• **Modulation of correlated biomarkers.** BP, as well as blood flow and arterial stiffness, are biomarkers that strongly correlate to PWV values. Non-healthy patients with hypertension or hypotension as well as those with high arterial stiffness, or with elevated BP values while doing sports, are critical cases that need to be modelled in the *in-vitro* and *ex-vivo* systems. The fabrication of elastic arterial phantoms with a broad range of distensibility properties, together with the inclusion of compliance chambers, resistance valves and pressure bulbs in the CVS setup may help to simulate the physiological characteristics of the arteries.
• **Implementation of bi-modal tissue.** Ideally, *in-vitro* and *ex-vivo* models should be able to validate not only one but multiple medical devices. Thus, the modelled tissue (via phantoms) has to represent all physical properties for which the devices should be validated. Bi-modal tissue implementations, i.e. phantoms with optical and acoustical properties or phantoms with electrical and acoustical properties (Cannata *et al* 2022) are being recently researched. Multilayer phantoms including various skin layers on top of the muscles and arteries increase the complexity of the *in-vitro* model but show more realistic approaches (Chen *et al* 2016). For tonometry applications, not only the tissue but also the bone structure should be included in the phantom.

### Advances in science and technology to meet challenges

Due to the individual variation of the characteristic values of PWV, BP, arterial thickness and compliance, there is a high need for fundamental and clinical research for patient-specific *in-vitro* and *ex-vivo* models (Kalaskar 2017). High-resolution scans (i.e. MRI) from the patient can be manually or automatically computerized (with intensity threshold for boundary identification and segmentation of certain parts of the body). After meshing the organ or body structure and adding the 2D slices into a single 3D structure, the model can be printed via a high-resolution 3D printer. Soluble-resin materials help with the fabrication of patient-specific moulds that will be used for the construction of patient-specific phantoms for *in-vitro* validations.

The possibility of creating patient-specific models allows the emulation of heterogeneous arteries with multiple bifurcations, several thicknesses and also stiffness conditions. Furthermore, variations in the thickness of the tissue can be also accurately modelled by using biocompatible hydrogel phantoms, where polyvinyl-alcohol flexible cryogenic materials can accurately assess arterial stiffness. In fact, by implementing realistic 3D heart models with 3D bioprinting (Mao and Wang 2022) the errors produced by the noise of peristaltic pumps can be suppressed.

*Ex-vivo* pre-clinical systems that include native blood vessels require bioreactor systems that simulate the physiological vessel flow conditions (Cooper *et al* 2021). The housing of the vessel in a cell culture medium (i.e. Dulbecco’s Modified Eagle’s Medium) at a constant temperature (37 °C) and under sterile conditions allows simultaneous and multimodal measurements to be performed under diverse hemodynamic conditions.

Multimodal and newly developed devices require highly robust and reliable *ex-vivo* models. Validation of these prototypes is done by the acquisition of CVS signals, and comparing them point-to-point to a reference measurement (e.g. catheter for BP measurements). Simultaneously, measurements using ECGs as synchronization signals will give insights into the accuracy, variance and precision of diverse technologies. The extracted pulse waveforms from the *in-vitro* and *ex-vivo* CVS can be better evaluated through probability analysis of Gaussian distribution and Bland–Altman plots. The Gaussian distribution provides the information if the pulse waveforms measured in the model are normally distributed. Furthermore, the mean and standard deviation of the measurements can be determined. Thus, the accuracy, validity, and reproducibility of the model can be predicted via the Gaussian probabilistic analysis (for example, when performing continuous measurements during a day in the same *in-vitro* model). Thanks to the Bland–Altman plots the agreement or disagreement between measurements performed by the newly developed device and the reference device can be statistically quantified.

### Concluding remarks

*In-vitro* and *ex-vivo* models provide a controlled environment to study CVDs and are essential to the preliminary validation of technologies, moving them forward on the TRL scale. New research is developing 3D models and more complex systems that would be able to emulate patient-specific stiffness and give a better prediction of vascular age. Sophisticated full-body 3D models can overcome some of the challenges of parameter evaluation and quantification in the lab.

### Acknowledgments

A B Amado Rey was financially supported by the Margarethe von Wrangell-Program (LaKoG BW, Germany).

## 11. Technical validation

### Alessandro Giudici^1,2^ and Bart Spronck^1,3^

^1^ Department of Biomedical Engineering, Cardiovascular Research Institute Maastricht (CARIM), Maastricht University, Maastricht, Netherlands

^2^ GROW Research Institute for Oncology and Reproduction, Maastricht University, Maastricht, Netherlands

^3^ Macquarie Medical School, Faculty of Medicine, Health and Human Sciences, Macquarie University, Sydney, Australia

### Status

Vascular ageing can be assessed via many different metrics, each measuring a different aspect of vascular function and structure. A growing volume of scientific evidence supports the potential clinical utility of assessing these metrics to improve CV risk prediction. However, the publication of validation guidelines/consensus documents that promote standardisation and ensure reliability of novel measuring devices constitutes a crucial step to transform this potential into an actual benefit for healthcare. These documents should detail both practical (e.g. environmental conditions, the reference standard: i.e. the measuring technique to be used as reference, the data acquisition protocol) and statistical aspects (e.g. minimum requirements of the study cohort and the statistical methods for the data analysis) to follow for a trusted validation of new non-invasive devices. In the past 15 years, groups of experts have made some first efforts to promote standardisation of the assessment of vascular ageing metrics. It is, therefore, pivotal that validation studies are performed in compliance with these directives. In this section, we focus mainly on haemodynamics measuring devices such as those measuring arterial PWV and BP.

ARTERY has had a leading role in writing guidelines/consensus documents for the validation of non-invasive haemodynamic measuring devices. In 2010, ARTERY published guidelines for the validation of devices which non-invasively measure arterial PWV (Wilkinson *et al* 2010), which were then updated in 2024 (Spronck *et al* 2024). These guidelines focus on two cardinal metrics: aortic PWV, for which an invasive catheter-based reference is recommended, and cfPWV, which may be validated against a non-invasive tonometry-derived reference. The initial (2010) validation guidelines were followed in 2017 by a consensus statement on the validation of non-invasive devices for estimating central BP (i.e. aortic pressure) (Sharman *et al* 2017). As for aortic PWV, the reference standard for central BP is obtained invasively: aortic BP measured with intra-arterial catheters. Furthermore, given the widespread use of BP measurements in clinical practice, the validation of any cuff-based or non-cuff-based BP measuring device should also comply with relevant standards by international organisations (e.g. ISO 81060–2:^2019^, ISO 81060–3:^2022^, respectively). Compared to the ARTERY consensus statement, these documents more broadly address the validation of any non-invasive BP measuring device.

Other vascular ageing metrics, such as flow mediated dilation, currently lack dedicated validation guidelines. In these cases, validation studies should, nonetheless, follow relevant measurement guidelines or consensus documents (e.g. Thijssen *et al* 2019).

Overall, although the short-term objective of validation guidelines is regulating and standardising new measuring devices, in the long term, these documents will indirectly promote the measurement of vascular ageing in clinical practice. As such, a concerted work towards standardisation will benefit all stakeholders involved in developing, manufacturing and using vascular ageing measuring devices.

### Current and future challenges

Despite previous efforts to standardise the validation of vascular age measuring devices, important challenges deserve further consideration. The first important issue is the beat-to-beat variability of human haemodynamics and, consequently, vascular ageing metrics (Xhyheri *et al* 2012, Parati *et al* 2018). This issue is practically relevant whenever readings of the reference standard and of the device-under-test are not performed simultaneously or when their measuring principle/technique differs considerably. In the context of BP measurements with sphygmomanometers, the 2019 ISO standard (ISO 81 060–2:^2019^) took significant steps to address this issue by providing separate directives when using sphygmomanometers or a continuous invasive BP measurement as reference standard. Because sphygmomanometers provide readings of systolic and diastolic BP over single heartbeats, their use as a reference requires adopting an R-T-R-T-R-T-R validation design (with R and T indicating measurements with reference sphygmomanometer and sphygmomanometer-under-test, respectively). Each T measurement is then compared with the mean of the preceding and following R readings. Conversely, when using continuous invasive BP as reference, the measurement with sphygmomanometer-under-test must be performed within the acquisition period of the invasive pressure. Furthermore, the measured beat-to-beat variability of the latter ($ \pm $1 standard deviation) is used to define a range of acceptance for the measurement obtained with the sphygmomanometer-under-test.

Another relevant issue is the repeatability and reproducibility of the measurements of vascular ageing metrics. The potential clinical usefulness of assessing vascular ageing is gauging a patient’s current level of vascular health and, perhaps more importantly, monitoring its evolution over time. Hence, high repeatability and reproducibility are paramount to ensuring that measured longitudinal changes of vascular ageing metrics reflect actual changes in vascular health. By requiring repeated measurements and assessing the bias and precision errors on both an individual patient and an individual measurement level, the ISO standard for the validation of cuff-based BP measuring devices (ISO 81 060–2:^2019^) introduced significant steps to evaluate repeatability and reproducibility. However, other guidelines (e.g. PWV) recommend performing repeated measurements solely to cope with haemodynamic variability but maintain a cross-sectional design. Furthermore, to further improve the reliability of vascular ageing devices, future guidelines should also recommend the direct assessment of the ability of a device to detect longitudinal changes in response to induced alterations in haemodynamics, which is currently not implemented in any guideline.

A third important issue is the rapidly expanding range of devices and techniques for measuring vascular ageing and the ongoing shift of CV research and the healthcare market towards automated and remote patient monitoring. While this approach has the potential to reduce operator dependence and increase the frequency of assessment of vascular health, specific guidelines for the validation of automated devices are currently lacking (Sharman *et al* 2022, Mukkamala *et al* 2023). This fact is assuming growing relevance as new devices gradually shift from *measuring* to *estimating* vascular age metrics (e.g. cuff-based central BP and PWV devices, wearables) (Mukkamala *et al* 2021). While a *measuring* device directly and physically measures a vascular parameter of interest, this new generation of devices relies on the combination of demographics, physical measurements of accessible haemodynamic waveforms and mathematical transformations to yield an *estimate* of a vascular ageing metric of interest (Mukkamala *et al* 2021, 2023). Because of this important difference in their working principle, there is an urgent need to define adequate protocols for validating this new class of devices, including the definition of appropriate reference standards and study designs.

### Advances in science and technology to meet challenges

The growing attention towards non-invasive, automated, remote, and continuous monitoring of vascular health is steering the focus of CV research and technology towards the *estimation* rather than the physical *measurement* of vascular ageing metrics. Because of their solid basis on physical principles, the validation of new *measuring* devices or techniques is relatively straightforward and reliable, provided that the observed range of the measured quantity is representative of the general population (ISO 81 060–2:^2019^). As such, a cross-sectional study design is sufficient to prove that the new device is not only able to assess cross-sectional differences in the measured metric but also to track its longitudinal changes (both acute and chronic) in one individual. Conversely, the same conclusions cannot be as directly drawn for devices that *estimate* vascular ageing metrics. Firstly, this class of devices may require periodic calibration against a validated *measuring* device, with the estimation accuracy expected to drop with intercurrent time from the last calibration. In the context of cuffless continuous BP monitoring, the Microsoft Research Aurora project analysed the ability of current wearable technology (e.g. PPG, tonometry, accelerometry, and ECG) to estimate BP one day from calibration (Mukkamala *et al* 2021, 2023). They found that estimation errors were already above the current guidelines’ acceptable threshold (Sharman *et al* 2017, ISO 81 060–2:^2019^). Therefore, future guidelines must involve validation study designs that allow for verification of the tested device’s longitudinal accuracy and set minimum requirements for their approval.

The Aurora project illustrated a second significant challenge for the development and validation of new wearable *estimating* devices. Ideally, such devices should exploit the information in the haemodynamic waveforms they *measure* to produce more accurate *estimates* than could be obtained from calibration and contextual variables alone. The Aurora project showed that measured data from current wearable technology did not improve the accuracy of the BP estimation beyond baseline regression models (i.e. models discarding any measurement from wearables and considering only the calibration BP and the time of the day) (Mukkamala *et al* 2021, 2023). These findings highlight the need for future guidelines to require transparency on the estimation accuracy of a ‘device’ with and without wearable-derived data and to set thresholds for improved accuracy to justify the use of wearable technology (Mukkamala *et al* 2021, 2023).

### Concluding remarks

Fast advances in technology are rapidly revolutionising our idea of medicine and health care, allowing for a remote, automated, personalised and potentially continuous assessment of clinical parameters. This paradigm shift has the undoubted potential to improve patient monitoring, favour early diagnosis of diseases, and reduce the economic burden of healthcare. However, a matching evolution of validation guidelines is mandatory to ensure that this revolution achieves the desired objectives. The principal aspects that need to be addressed are:

(1) Defining clear rules on how to deal with beat-to-beat variability which match the working principle of the different devices under study;
(2) Incorporating the assessment of repeatability and reproducibility;
(3) Incorporating the evaluation of the longitudinal performance in response to haemodynamic changes; and
(4) Requiring transparency on the estimation accuracy of wearables with and without wearable-derived data.

## 12. Assessing clinical utility

### Rosa Maria Bruno^1,2^, Pierre Boutouyrie^1,2^ and Thomas Weber^3^

^1^ INSERM U970 Team 7, Paris Cardiovascular Research Centre

^2^ PARCC, University Paris Descartes, AP-HP, Pharmacology Unit, Hôpital Européen Georges Pompidou, 56 Rue Leblanc, Paris 75015, France

^3^ Cardiology Department, Klinikum Wels-Grieskirchen, Wels, Austria

### Status

Any new technology assessing vascular aging (VA) must demonstrate to be physiologically sound and accurate for what it claims to measure; furthermore its intended use, which represents its clinical utility, should be defined, in line to what requested by the current Medical Device Regulation (MDR). According to MDR classifications, vascular ageing measuring devices reasonably belong to the category diagnosis/monitoring of a disease. More specifically, an accurate assessment of vascular age, opposed to chronological age, is meant to identify those individuals with EVA, who have a risk of cardiovascular (CV) events greater than expected (based on their risk profile from traditional risk factors such as sex, lipids, blood pressure (BP), smoking status and diabetes), and those with supernormal vascular ageing (SUPERNOVA), whose vascular age is lower than expected based on their risk profile (Bruno *et al* 2020). In other words, the new technology is meant to be used to improve risk stratification, which means to achieve a more accurate prediction of CV events than commonly used risk calculators (in the case of Europe, SCORE2 and SCORE-OP). Indeed, risk calculation is mainly driven by non-modifiable factors such as age and sex (Score2 Working Group and ESC Cardiovascular Risk Collaboration 2021). For instance, being a man over 70 with one risk factor leads to very high risk, whereas being a woman aged 50 with 2 risk factors leads to low risk. This leads to imbalance in care resources. This is where biomarkers (Wang 2011), also called risk modifiers (Visseren *et al* 2021) can be a game changer. Conceptually, individuals with EVA are either more susceptible than others to traditional CV risk factors, or exposed to unidentified (e.g. genetic) detrimental risk factors, whereas individuals with SUPERNOVA are either less susceptible than others to traditional CV risk factors, or protected by unidentified (e.g. genetic) factors. Given the proportions of the problem (CV disease being the first cause of morbidity and mortality worldwide), and the limitations of the current approach (a majority of CV events occur in people classified at low risk (Cooney *et al* 2009)), the clinical utility of such a technology is undeniable. The above-mentioned scenario of clinical utility implies thus a broad utilisation of the technology, especially in low-risk populations. A number of tests assessing vascular ageing are indeed currently prescribed (and reimbursed by national healthcare systems and health insurances) under this application worldwide: this is the case for CT-based Coronary Artery Calcium (CAC) score and carotid US in Europe, and of many other tests (including flow-mediated dilation and baPWV) in far-east countries such as Japan. An alternative scenario considers that the new technology will be used in a subset or disease for which a specific unmet need has been identified, to guide clinical decisions (i.e. to prescribe a confirmatory test, to treat/not to treat, indication to a specific drug…). The new technology will be thus used in a defined population in whom the actual gain in using it is maximal.

### Current and future challenges

Demonstrating the clinical utility of vascular ageing biomarkers raises several challenges:

(1) finding the best way to assess vascular ageing. This is not obvious, and includes finding the ‘best’ single arterial measurement, or a combination of arterial measurements, reflecting both atherosclerosis (coronary artery calcium, carotid artery intima media thickness, etc) and arteriosclerosis (one of several PWVs, central hemodynamics such as central pulse pressure, amplitudes of forward or backward waves, etc);
(2) finding the best metric to define vascular ageing, for instance age- and sex-adjusted cutoff-levels of completely healthy or population-derived samples, or single cutoff-values (as we use for BP and lipids), or cut-off values which vary according to patient’s risk;
(3) identifying the ideal target population, as previously discussed;
(4) to develop a clinical management strategy based on vascular ageing, basically non-pharmacological and pharmacological approaches to slow or even reverse vascular ageing;
(5) demonstrating that this strategy improves clinical outcomes: in other words, a treatment strategy based on the level of the studied biomarker is superior to usual care for prevention of CV events (Wang 2011).

This implies the design of clinical trials in which individuals are randomised to a biomarker-based treatment strategy or usual care; in the active arm, those presenting an altered biomarker of vascular ageing will be treated accordingly. Recently, some trials testing CAC as vascular ageing biomarkers provided inconclusive results (Greenland and Polonsky 2022); however, increased calcium score may capture individuals with advanced vascular disease, in which preventive strategies are less effective to slow or reverse vascular ageing.

### Advances in science and technology to meet challenges

Many promising technologies assessing vascular ageing could be used in such trial designs because they demonstrated incremental predictive value over and above standard risk assessment, such as carotid-femoral and baPWV (Ben-Shlomo *et al* 2014, Ohkuma *et al* 2017). Governmental funding agencies, manufacturers, clinicians and researchers need to join forces to promote this kind of studies, run cost-effectiveness analyses, and promote effective targeted preventive strategies for a broad population. Novel trial designs, such as decentralised, pragmatic and registry-based clinical trials should be used to increase feasibility of such studies, as recently recommended by many scientific societies in CV medicine (Bowman *et al* 2023).

Another approach could be to perform smaller studies in subsets of the population in whom the measurement of vascular ageing conveys the maximal gain over usual care, or for which surrogate endpoints are accepted. For example: it has been repeatedly shown that development of arterial stiffness precedes the development of hypertension (Saz-Lara *et al* 2022). This may be used to screen for increased arterial stiffness in a defined subsample of the population at risk of developing hypertension, which then will require comprehensive non-pharmacological interventions, with development of hypertension as main outcome. The vascular ageing-technology would then be incorporated more quickly in clinical algorithms for specific conditions, and their use can be further extended as a subsequent step.

Finally, a third approach would be longitudinal monitoring, which implies the assessment of the vascular ageing biomarker several times throughout the life span to guide therapy. Promising data about this approach have been obtained for cfPWV (Laurent *et al* 2021).

### Concluding remarks

In conclusion, assessing the clinical utility of any biomarker/risk modifier is a fundamental step of the development of vascular ageing technologies and determines the application of the technology in routine clinical practice. Although observational studies are the key to identify promising biomarkers, the clinical utility can only be accurately determined by randomised clinical trials. Innovative study designs are mandatory to do that cost-effectively in a timely manner. In summary, it is clear that proper prospective trials and cost-effectiveness analyses are required for implementation of specific measures/techniques/algorithms into clinical practice.

### Acknowledgments

This article is based upon work from European COST Action CA18216 ‘Network for Research in Vascular Aging’, supported by COST (European Cooperation in Science and Technology, www.cost.eu).

## 13. From research to market: medical device regulation

### Elisabetta Bianchini^1^ and Christopher Clemens Mayer^2^

^1^ Institute of Clinical Physiology, Italian National Research Council (CNR), Pisa, Italy

^2^ Center for Health & Bioresources, Medical Signal Analysis, AIT Austrian Institute of Technology GmbH, Vienna, Austria

### Status

Vascular aging (VA) results in functional and structural alterations of the arteries (e.g. pulse wave characteristics, geometrical and elastic parameters, reactivity, pulse pressure, and vessel structural composition). These alterations can be assessed by analysing images or signals acquired through different technologies (Climie *et al* 2023a). Devices on the market include medical imaging equipment (such as US, MRI, and CT/positron emission tomography (PET) machines), non-invasive sensor-based methods like tonometry, oscillometry, PPG, and sphygmomanometry, intravascular systems (like catheters for plaque or hemodynamics analysis), and software for data processing, which may be standalone or integrated into other systems. Measures of vascular ageing can provide added value for diagnosis, prevention, monitoring, treatment, or alleviation of the related diseases; the adopted systems are generally qualified as *medical devices* and, hence, need to be developed and validated according to specific rules and to be approved by authorized entities for commercialization (Mayer *et al* 2021). Regulation of medical devices was introduced in the 20th century to ensure safety for users and public health. More specifically, in 1976, with the Medical Device Amendments to the FD&C Act (Darrow *et al* 2021), the USA introduced a new legislation specifically focused on devices. Nowadays, a regulatory pathway is a mandatory step to enter the world’s main markets and, thus, allowing the transition of technologies from research to practice.

Generally, a risk-based approach is adopted within the whole lifecycle of a system focusing on users’ safety. When undergoing regulatory risk-based classification, most devices assessing vascular ageing fall into a medium-to-high risk class in many regulatory frameworks (e.g. MDR 2017/745 for the EU, 21 CFR Parts 800–1299 by FDA for the USA, TGA for Australia) (Mayer *et al* 2021). These require dedicated procedures and competences by the manufacturer for the implementation of a successful strategy leading to the need for additional resources and structures, which might not be immediately available e.g. in small enterprises, in academia or for single inventors. Consequently, innovative systems might lack approval as medical device and start-ups might be forced to translate into other markets.

It is crucial to consider regulatory requirements starting from the design phase for the acceleration of innovation. These requirements can support effective processes for the developers aimed to guarantee users’ safety and allowing the introduction of a new medical device on the market serving the society (Bianchini and Mayer 2022).

Dissemination of a regulatory culture in different fields, interaction between stakeholders, and subsequent updates to legislation, such as the recent European Medical Device Regulation (MDR) (EU 2017), are very important for further advances to ensure a smooth transition from research to market. A robust transversal pipeline, based on the integration of different perspectives, leads to more effective processes. The increase in effectiveness follows the introduction of more specific requirements and the implementation of references and tools to provide better transparency and traceability. A harmonized balance between regulation and innovation remains a crucial point to guarantee the best medical solution for the users.

### Current and future challenges

**Figure 14.**
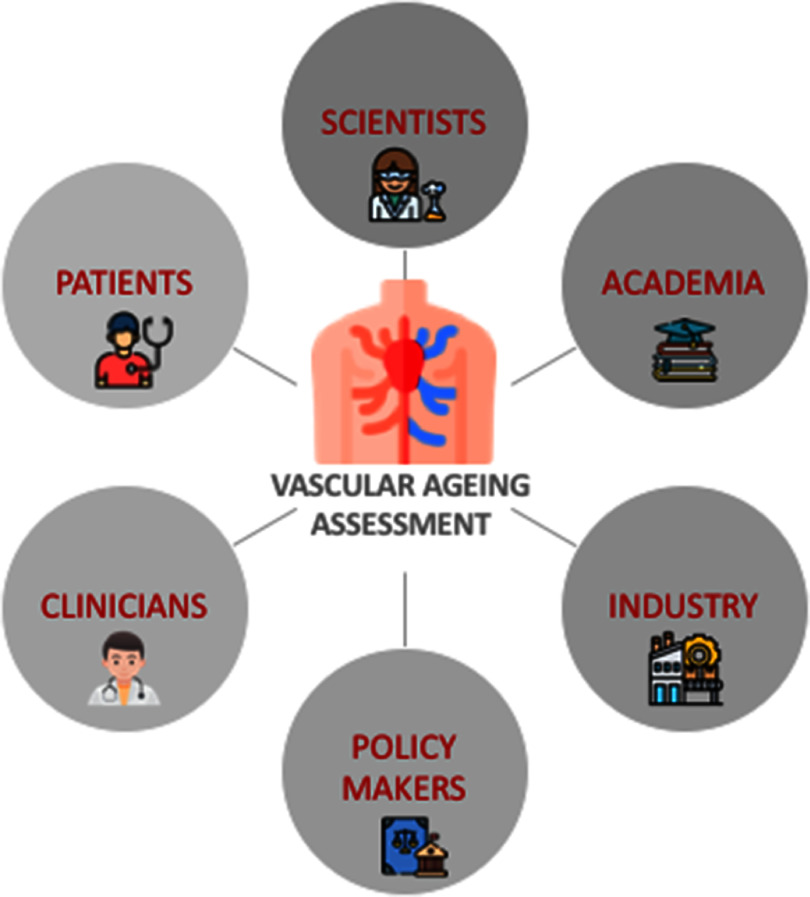
Key actors involved in the development, maintenance and use process of a vascular ageing medical device. Icons’ source: www.flaticon.com. Reproduced with permission from Flaticon.

Challenges are mainly related to cultural and operative aspects. An effective regulatory framework implies active involvement of the main actors (see figure 14) related to the development, maintenance, and use of medical devices in general and especially for assessing vascular ageing. A key point for a successful and safe adoption of medical technologies is awareness of the regulatory approach by all involved stakeholders. Without this awareness pitfalls emerge in different phases of a system’s lifecycle to the disadvantage of patients and operators. A lack of regulatory culture can lead to the absence of mandatory requirements for the product slowing the transition to clinical practice, and even to deficiencies in aspects related to the users’ safety, e.g. the post-market and surveillance activities.

Besides the cultural issue, also operative difficulties must be considered. Worldwide, ever-evolving and demanding regulation frameworks require additional resources challenging especially small companies developing innovative solutions; the scenario is fragmented, and, even if key concepts are in common for different parts of the world, requirements are not totally harmonized, and specific application processes are related to the geographical area. Moreover, recent legislations, such as the EU MDR, have increased requirements related to technical and clinical evidence, but guidelines for the validation of, e.g. vascular ageing biomarkers might not yet be fully harmonized or are not available at all. It is worth noting that, besides safety and technical performance, solid and harmonized scientific data are needed to show clinical benefit. This evidence cannot be delegated to manufacturers and can only be obtained by a common effort with a central role of science (Fraser *et al* 2018, Bianchini and Mayer 2022).

Finally, complexity and updates of regulatory frameworks can lead to further concrete external barriers for manufacturers of devices assessing vascular ageing. For example, the recently introduced European legislation, MDR (EU 2017) has increased the time constraints due to the need of renewed approval for all medical devices on the market and subsequent overload of notified bodies. For this reason the transition period has been recently further extended.

Related to current/future challenges, specific attention should be placed on medical device software (MDSW). MDSW, being mainly based on processing of biomedical data, is central for personalized medicine and for vascular ageing assessment. A more demanding approach for development and maintenance of MDSW is now required and it can be satisfied only by a multidisciplinary approach including a regulatory lifecycle mindset. The challenge is even more complex for the emerging AI technologies (Bikia *et al* 2021a) since the consideration of further characteristics is needed to guarantee safety and benefit for users. Attention around this topic is huge and international initiatives are ongoing (Giansanti 2022). Harmonization and collaboration among different actors are needed (Pesapane *et al* 2018) to face with a key challenge: finding the best trade-off to not kill innovation and guarantee safety.

### Advances in science and technology to meet challenges

The challenges mentioned above can be primarily addressed by increasing interaction and mediation between key actors involved in the development/maintenance process of vascular ageing medical devices. Initiatives creating opportunities for exchange among scientists, clinicians, academia, industry, and policy makers, like the COST Action vascular ageing network (VascAgeNet) (Climie *et al* 2020), are desirable since they contribute to building a collective intelligence and a shared language able to increase not only the communication among different roles, but also its quality and effectiveness. From an entrepreneurial point of view, this could also increase the possibility for small start-up companies to train internal resources in order to provide faster reaction to the ever-evolving challenges in the field. In addition, education about MDR for scientists, clinicians, and innovators can help to improve their perception of their potential impact. An active and conscious attitude is essential to provide/receive inputs to/from the policy makers and manufacturers and to underline the crucial role of science in supporting the adoption of rigorous methodological approaches within different phases of the innovation pipeline.

The introduction of a regulatory culture within the vascular ageing community is a fundamental step that, by creating awareness of its importance, can also help to solve operative issues. Firstly, existing research generally means innovation; thus, legislation should not be seen as a barrier to progress but as a stimulus to consider safety and performance requirements since the design phase. Secondly, new studies and the aggregation of clinical data from already existing registries could provide further evidence regarding the added value of vascular ageing assessment in clinical practice supporting and facilitating the development and approval of new solutions able to improve patient management. Moreover, the availability of big data sources could ensure an unbiased validation process for innovative medical devices for assessing vascular ageing, like those based on AI technologies. It is worth noting that guidelines and standards by scientific experts are required and new projects contributing to their development should be designed, since they can support concretely the paradigm shift towards evidence-based modern regulation (Fraser *et al* 2022). Moreover, ideas from an aware community could lead to the invention and development of tools able to facilitate steps of the lifecycle of a medical device, such as traceability and documentation control.

### Concluding remarks

**Figure 15.**
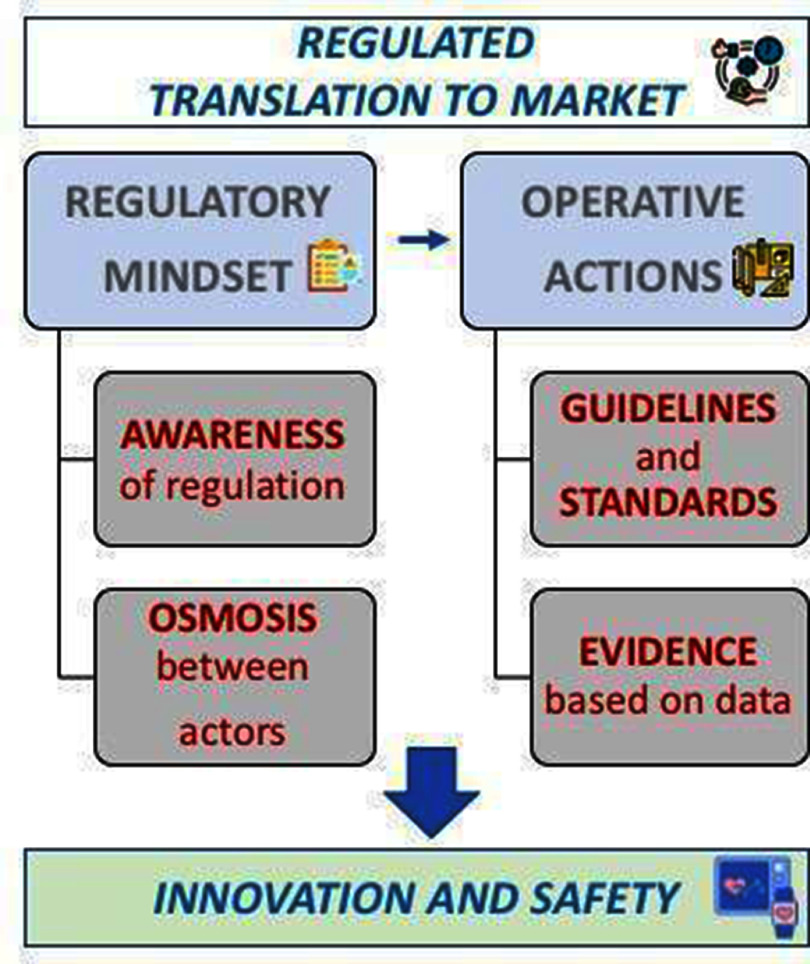
Main regulatory challenges and potential steps that can be supported by the scientific community for improving the process of translation from research to practice in the vascular ageing field. Icons’ source: www.flaticon.com. Reproduced with permission from Flaticon.

The vascular ageing community can play a crucial role in the future of medical devices in the field. Addressing the mentioned challenges can concretely speed-up the introduction of new preventive and clinical paradigms into practice leading to innovation, safety, and a smooth transition from research to clinical practice. Summarized (see figure 15), the regulated translation to market needs a regulatory mindset, i.e. awareness and an osmosis between different actors, and operative actions related to guidelines and standards for the evidence-based technical and clinical validation.

### Acknowledgments

This paper is based upon work from the European COST Action CA18216 ‘Network for Research in Vascular Ageing’, supported by COST (European Cooperation in Science and Technology, www.cost.eu).

### Conflict of interests

Elisabetta Bianchini is co-founder of QUIPU s.r.l., Pisa, Italy a spin-off company of the Italian National Research Council and the University of Pisa developing software medical devices.

## 14. Commercial translation of academic innovations

### P M Nabeel^1^ and Jayaraj Joseph^2^

^1^ Healthcare Technology Innovation Centre, IIT Madras, Chennai 600 113, India

^2^ Department of Electrical Engineering, Indian Institute of Technology Madras, Chennai 600 036, India

### Status

Translational research is a critical pathway in bridging the gap between laboratory discoveries and their practical application in improving patient outcomes, particularly in the realm of vascular ageing. The commercialization of new medical devices emerging from academic research plays a pivotal role in addressing the challenges associated with vascular ageing, such as the prevention and treatment of age-related vascular diseases. This process involves translating complex research findings, including those related to the mechanisms of vascular ageing and innovative interventions, into practical solutions that healthcare practitioners and industry stakeholders can effectively implement. As (Mayer *et al* 2020) highlight, the journey from idea inception to market introduction is fraught with challenges, requiring not only significant investment and time but also a multidisciplinary approach that includes researchers, healthcare professionals, and industry participants to ensure successful commercialization. Despite these hurdles, the effective translation of research on medical devices targeting vascular ageing can lead to breakthroughs that significantly enhance clinical practices and patient care, underscoring the importance of rapid and efficient implementation of technological innovations in combating the effects of vascular ageing.

**Figure 16.**
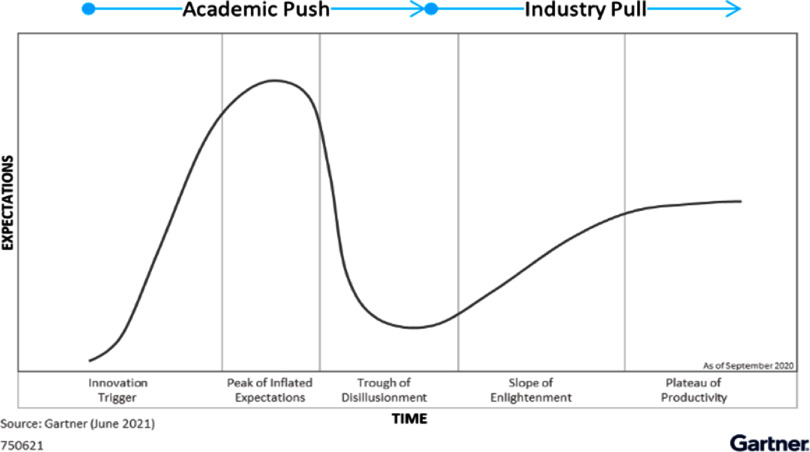
Gartner Hype Cycle (Gartner 2023). GARTNER is a registered trademark of Gartner, Inc. and/or its affiliates and is used herein with permission. All rights reserved. Reproduced with permission from Gartner (2023).

Universities increasingly focus on generating intellectual property (IP) and commercialising their patents at larger scales (Marr and Phan 2020). A common way of approaching this is to establish centres with a focus on academia-industry collaboration, which engages with leading industry players to develop commercialisation strategies for academic innovations. This often leads to technology licensing agreements, or spinoff companies focusing on commercial adaptations. The *open innovation* approach (Bigliardi *et al* 2020), understood as balancing the needs of collaborative idea-sharing with commercial interests and market adaptation, is generally accepted as producing greater innovation and technology transfer. However, innovations go through different stages of market expectations, as described by the Gartner Hype Cycle (figure 16) (Gartner 2023). Innovations from academia, especially from the healthcare domain, have their own challenges and opportunities during the commercialisation process. Industry partnership is vital to enable the invention to pass from the university-push phase to the industry-pull phase (figure 16), through the *Trough of Disillusionment*, and into an established presence in the market. In the following, a successful example is presented.

**Figure 17.**
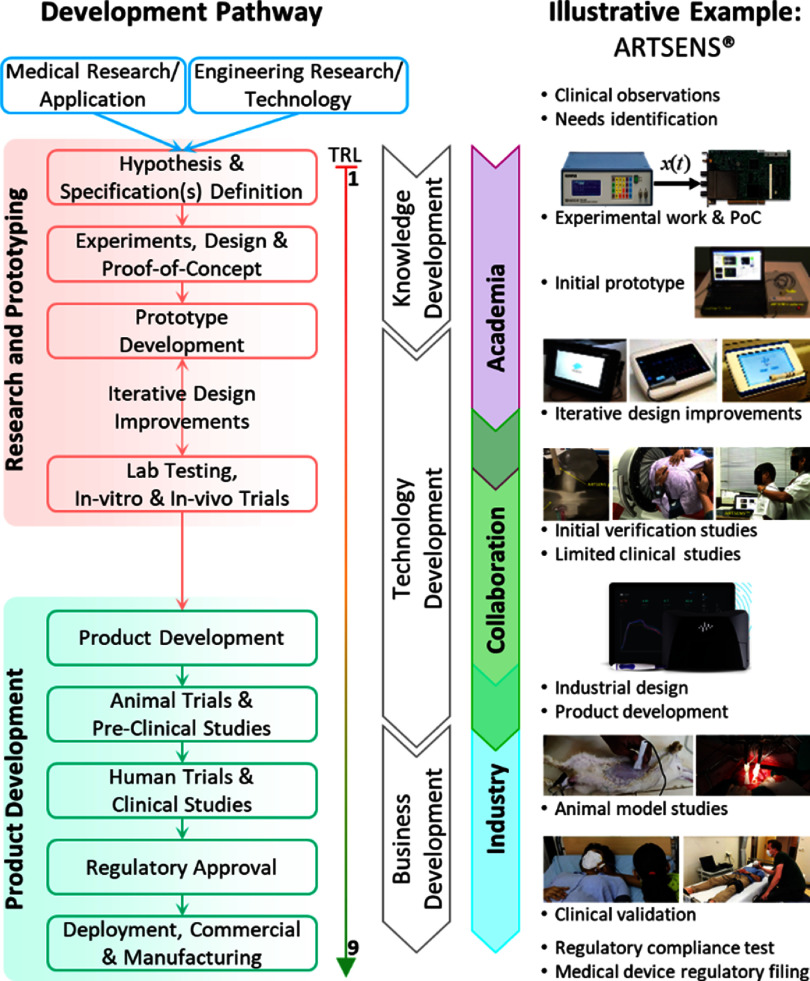
Medical technology development pathway with an illustrative example of ARTSENS® device—an academic-driven vascular ageing and fitness assessment technology. Reproduced with permission from Artsens.

The ARTSENS®, developed by the Indian Institute of Technology (IIT) Madras’s Healthcare Technology Innovation Centre, is a successful academic-driven translational research in the EVA space. It is an image-free US device for automated measurement of clinically relevant vascular health markers, facilitating a quantifiable screening process for vascular ageing and enabling early intervention and preventive measures (Nabeel *et al* 2022). ARTSENS® is the result of a PhD research begun in 2008 with a commitment to producing an industry-ready device. It was achieved through a series of evolutionary developments, multidisciplinary collaborations, iterative verifications, and clinical validations. The result is an ergonomic product with a technology readiness level (TRL) of ∼8, ensuring a reliable and usable workflow that functions effectively not solely under laboratory conditions, but in clinical settings as well. The device was developed in consultation with physiologists, clinical and industry partners, and regulatory experts, who were involved in various parts of the developmental stage (figure 17). IIT Madras’s adoption of the open innovation approach, which strategically involves the right stakeholders and peer-to-peer collaboration (both among universities and university-industry), has enabled an academic institute to produce a high-TRL product. However, the percentage of such technology being commercially translated to the market is abysmally low compared to global academic publications (Brightman *et al* 2021).

### Current and future challenges

The innovation pipeline in healthcare technology research differs substantially from the overall R&D culture in other academic fields, whose research outcomes have themselves been traditionally slow in being commercially implemented (Nabeel 2023). In spite of the high student and researcher turnover rates and the nature of research grants, medical device innovations and evidence-based research outcomes take more than a decade, on average, to be translated into standard practice (Morris *et al* 2011). The medical-innovation-to-healthcare-industry pipeline is clogged by interconnected barriers, such as: insufficient market research and relevant ideation; lack of effective design controls; poor risk management; inadequate documentation; and lack of quality management. Often, university faculty or academic research leads have an incomplete understanding of the market and the potential impact of their innovation, augmented by poor communication with industry stakeholders. The lack of business development and marketing skills in academia widens the communication gap between research departments and the fragmented healthcare device market. There are also academia-specific challenges in medical device development and translation, such as: proofs of concept failing to attract adequate funding; development slowed by ineffective project management; and inefficient, collaboration-hindering IP policies within or among universities (Nabeel 2023).

There is also the regulatory aspect to consider (see section 13). Devices developed for screening of vascular ageing use are subject to regulatory approval before they can be commercially used (Mayer *et al* 2021). Different regulatory bodies worldwide have their own regulatory platforms and rigorous safety requirements. Universities tend to have limited knowledge of the regulatory framework and requirements that greatly influence the duration of market translation, such as classifications of medical devices, quality management, risk assessment, clinical and technical evaluation standards, regulatory submission, and marketing approval entities. However, from an academic standpoint, it is impractical to implement and document the entire regulatory process at the R&D stage (Letourneur *et al* 2021). Further, the regulatory requirements and complexity of the approvals process have been increasing in recent years to ensure consistent health and safety protections in adapting scientific and technological breakthroughs. Europe’s new stringent regulatory environment, with full enforcement of the Medical Device Regulation 2017/745 (Mayer *et al* 2021), changes the academia-to-industry translation landscape, and contributes to an increase in product development and validation costs. This is bound to reflect in higher investments, borne by industry and state enterprises.

### Advances in science and technology to meet challenges

The market adaptation process for vascular ageing medical devices is different from the translational pipelines for biologics and pharmaceuticals. This highlights the need for academic institutions to be familiar with domain-specific knowledge, mechanisms, and best practices. A crucial first step is improving academic research culture by including new, relevant educational and research approaches among students, research scholars, and faculty (Brightman *et al* 2021). A focused, systematic approach to overcome the structural barriers impeding academic research can significantly increase the rate at which innovations are translated into the medical device market. For instance, many universities have set up centres of excellence which focus on developing proof-of-concept prototypes (Manyazewal *et al* 2022), facilitated by Technology Transfer Officers, who help academics hone their entrepreneurial, market knowledge, and product development skills. Certain aspects, such as market search, economics thinking, patent assignment, and obtaining clinical trial resources, would benefit from the active involvement of industry-based advisors.

Alongside effective IP management (Marr and Phan 2020), it is also important for universities to adopt the open innovation model, which enables timely knowledge-sharing, efficient publication rates, and joint research among universities and peer industries (Bigliardi *et al* 2020). This approach, analogous to a project with team members from multiple disciplines, helps to cover more bases and to make the translational pipeline more efficient. Efforts should be made to strengthen and facilitate collaboration among the global network of academicians, scientists, engineers, clinicians, and medical companies. Researchers working on a medical technology should begin looking at its commercialisation potential once it reaches a TRL of four or five. This could include industry collaboration, starting up, studying the market landscape and identifying product placement strategies. These steps also enable the technology to adhere to accepted design practice, clinical validation, and compliance with regulatory processes (Lottes *et al* 2022)—which are often challenging to incorporate in an academic environment. Support from societies such as VascAgeNet will help to develop streamlined multidisciplinary teams in vascular ageing translational research. An R&D ecosystem (Lottes *et al* 2022), which emphasises translational research, is critical to ensuring the sustained conversion of research innovations from publications into products. An effort is also required to envision and create international resources, core infrastructure facilities, and funding to support early-stage evaluations of medical technologies, regulatory requirements, and efficiently tackle any other challenges unique to medical device commercialisation.

### Concluding remarks

The commercialisation of breakthroughs in vascular ageing research holds the potential to profoundly extend lifespan and improve the quality of life by moving beyond traditional disease-centred treatments to address the root causes of vascular deterioration. Academic institutions, as leading contributors to this field, are at the forefront of developing innovative, patient-focused medical devices that target the mechanisms of vascular ageing. Despite this, the inherent research culture within universities has often hindered the seamless transition of their groundbreaking findings into viable commercial products. This gap underscores the urgent need for a more robust, multidisciplinary approach that bridges academia and industry. By fostering closer collaboration between clinical researchers and universities, encouraging entrepreneurial initiatives, and enhancing regulation-supportive ecosystems, we can significantly improve the translation of vascular ageing research into practice. Such a strategic shift is crucial for ensuring that the next generation of medical technologies—not only identifies but also effectively combats the complex processes of vascular ageing—can be rapidly and efficiently delivered from the laboratory to the market. This approach promises to revolutionize our ability to mitigate the effects of ageing on the vascular system, offering hope for longer, healthier lives through the innovative application of research breakthroughs.

## 15. Influencing policy makers

### Chloe Park

MRC Unit for Lifelong Health and Ageing at UCL, 1–19 Torrington Place, London WC1E 7HB, UK

### Status

To have a lasting impact on society, scientific findings need to inform practice and policy. *The* science-for-policy communication *process can occur at all levels of governance* (*local, national, and international*) *and* can be instigated by both scientists and policy officials in a bidirectional manor.

Evidence-based science should be at the centre of policy making, however, becoming a successful policy influencer is not a priority when training to become a researcher, clinician or engineer in the vascular ageing field. Help is required to navigate the policy landscape as it requires public affairs expertise, knowledge of the current landscape and strategic planning for successful dissemination of well-timed, impactful outputs.

A 2020 systematic review (Cairney 2020), targeted at academics, provides key tips to increase the success of influencing policy. The following section addresses these recommendations in the context of the vascular ageing field, including current strengths and future considerations.

**1. Produce high quality research**

Policy evidence should be legitimate and unbiased, which requires an interdisciplinary approach. Each discipline provides a different perspective to performing critical analysis and suitable interpretation of results to ensure high quality outputs. Research should always be peer-reviewed and clearly communicate both the limitations and the strengths and weaknesses of the research.

An example of this interdisciplinary approach in the vascular ageing field is the COST Association European VascAgeNet. VascAgeNet is made up of over 350 experts from leading institutions across 40 different countries. The interdisciplinary and intersectoral nature of the network combines medical research and clinical aspects from various disciplines (hypertension, cardiology, nephrology, physiology, etc), engineering (electrical engineering, mathematics, physics), hospitals, universities, research organisations and industry.

**2. Make the research relevant and readable**

Policy makers are aware that CVD is a substantial public health challenge, as shown by the inclusion of CVD in the European Commission’s Healthier together—EU non-communicable diseases initiative. European_Commission (2019). If individuals at increased CV risk can be identified at an asymptomatic, subclinical stage this will lead to a substantial decrease in both health and economic burden. Strategies for achieving healthy vascular ageing and preventing premature vascular ageing are both attractive and relevant to policy makers. To successfully engage with stakeholders the research needs to be tailored to its audience. Evidence must be framed appropriately using relevant language in brief, concise, open access reports.

Researchers in the vascular ageing field should be mindful of how their work fits into the bigger landscape. Although vascular ageing on it is own may not be a current public health priority, many risk factors associated with EVA are priorities and keeping up to date with current policies will help to increase future policy engagement success. These areas include:

**Prevention strategies:** Many global CVD policies prioritise prevention efforts aimed at reducing risk factors associated with vascular ageing, such as hypertension, atherosclerosis, and arterial stiffness. These strategies often include promoting healthy lifestyles, reducing tobacco use, improving diet quality, and encouraging physical activity—all of which have direct implications for the vascular ageing field.

**Public health campaigns and awareness-raising:** Policymakers also invest in public health campaigns and initiatives to raise awareness about the importance of CV health and encourage healthy behaviours. These campaigns often target specific population groups or risk factors, such as smoking cessation programs, healthy eating campaigns, and initiatives to promote physical activity. Researchers should consider becoming a part of these initiatives.

**Early detection and diagnosis:** Screening programs and diagnostic guidelines can be designed to identify individuals at risk of developing vascular complications, allowing for timely interventions to slow down the ageing processes in blood vessels. Researchers should create policy documents to explain how the recent advances in the field (new evidence, technologies, early interventions and treatments) mentioned throughout this document can help with future efforts. Including how the work addresses cross-cutting factors like health inequalities, upscaling innovations and how these contribute to economic growth by ensuring that the workforce are healthier.

**Research funding and collaboration:** Policymakers support research initiatives and collaborations aimed at advancing the understanding of CVD and developing innovative approaches for its prevention and treatment. Funding programs such as Horizon Europe allocate resources to support CV research projects and promote collaboration among researchers and institutions across Europe. Researchers should regularly scan calls to see if their vascular ageing research fits the remit. They should also highlight gaps that require funding by being involved in policy consultations and workshops.

**Implementation science:** Policymakers are also interested to know what actually works in the real world. How can we successfully detect people with EVA, how do we best explain risk to individuals, how do we ensure that people stick to health modifications/interventions when EVA is detected and how do we evaluate all of the above.

**3. Understand policy processes**

Researchers in the vascular ageing field are required to understand that policy change does not only imply a change in legislation, but the research can also influence non-legislative decisions such as regulations and resource allocation. Regularly check recent political developments in the field via the website of any relevant departments. Investigate which policymakers work in this area, build a relationship with their special advisors, and disseminate research summaries to all relevant committees. Submit oral and written evidence whenever possible. Approaching officers of backbench committees for assistance in promoting an issue is a more informal way to put pressure on policy makers (UKRI).

**4. Be accessible to policy makers**

Communication is essential to ensure that policy makers are made aware of the findings. How to best influence policy makers depend on their roles, therefore researchers need to strategically time and tailor communications with politicians and officials including central and local government, non-departmental public bodies, regulatory bodies, non-ministerial departments, political parties and activists in think tanks pressure groups. Remember that science advice can be provided at different stages of the policy-making process. Build relationships with science-advisory bodies/structures that act as intermediaries between scientific communities and policy makers (UKRI).

**5. Build relationships with policy makers**

Building bidirectional relationships with policy makers is the key to success. Now is the time to seek out which policy makers are interested in the vascular ageing vision and talk with them about the research goals. An important consideration is that movement within political departments is common. Therefore, forming relationships with those who remain in more stable roles (such as civil servants who specialise in the technical aspects of policy) is advised.

### Current and future challenges

The good news is that vascular ageing measures already feature in some policy documents. Recent examples from 2022 include the effect of air pollution (SLATER 2022), vaping (WHO 2022), smoking and diabetes (CADTH 2022) on arterial stiffness (see table 1 for more details and examples). The bad news is that there is no policy document dedicated to vascular ageing. The biggest current challenge is that the vascular ageing field is fragmented, and the technology is not ready to take to policy makers. However, this should not deter the team from setting the groundwork required to build relationships with key policy makers now to ensure that the channels of communication are open when impactful outputs need to be shared in the future. This includes the preparation of influential materials and recommendations, including interim ones.

**Table 1.** A list of recent policy documents from around the globe that use vascular ageing research as evidence to inform policy.

Policy document	Date	Policy maker	Link	Vascular ageing evidence used in document
Air pollution and the world of work: policies, initiatives and the current situation a scoping and evidence review for Southeast and East Asia	October 2022	Stockholm Environmental Institute	Slater *et al* (2022)	Wu *et al* (2010)
Nicotine vaping in England: 2022 evidence update	September 2022	The UK Government	Office for Health Improvement and Disparities (2022)	Office (2022)
Sodium-Glucose Cotransporter-2 Inhibitors for Type 2 Diabetes Mellitus	September 2022	Canadian Agency for Drugs and Technologies in Health	Cadth.ca. (2022a)	Wei *et al* (2022)
Maladie de Kawasaki	September 2022	Haute Autorite de Sante	Haute Autorité de Santé (2022)	Cheung *et al* (2004)
Opinion on electronic cigarettes.	August 2022	Publications Office of the European Union	European Commission (2022)	Vlachopoulos *et al* (2016)
Impact of the 2017 American College of Cardiology/American Heart Association Hypertension Guideline on the prevalence of hypertension in young Saudi women	August 2022	World Health Organisation	Al-Mohaissen *et al* (2020)	Nicoll and Henein (2018)
Smoking and cardiovascular disease in the Eastern Mediterranean Region	August 2022	World Health Organisation	World Health Organisation (2018)	Salahuddin *et al* (2012)
Tobacco: preventing uptake, promoting quitting and treating dependence	August 2022	NICE	NICE (2021)	Chaumont *et al* (2018)
Melatonin for the Treatment of Insomnia: A 2022 Update	May 2022	Canadian Agency for Drugs and Technologies in Health	Cadth.ca. (2022b)	Kim *et al* (2021)
Mat vid diabetes Food in diabetes	May 2022	Swedish Agency for Health Technology and Assessment of Social Services	Swedish Agency for Health Technology and Assessment of Social Services (2022)	Ahola *et al* (2018)
A matter of life and death	March 2022	Health Foundation	The Health Foundation (2022)	Ahola *et al* (2018)

A second challenge is that there is no simple way to know when the research will be in demand in a policymaking system. While a senior politician may currently have CVD prevention and vascular ageing as a priority their priorities may shift, or they could move on and their successor may not consider vascular ageing as a priority. The political process is complex and multi-channelled, navigating the system successfully requires members of the action to be skilled in public affairs and the policy landscape.

A third challenge is that influencing policy takes time especially when it is difficult to access some networks or there is competition for the policy maker’s attention. A strength to large multi-disciplinary, multi-country networks, like VascAgeNet, is that policy makers can be approached from various angles.

A fourth challenge is securing funding to support multidisciplinary collaborations that can advance the vascular ageing field, in times of crises and an increasingly difficult funding environment.

### Advances in science and technology to meet challenges

To meet the challenges, those in the vascular ageing field need funding and resources (both time and people) dedicated to policy engagement. While the development pipeline steps are being refined, clear and feasible objectives can be formulated to influence policy makers. **Pathways to building relationships with policy makers include** signing up to an expert register or third science-policy party organisations such as The European Parliamentary Research Service—EPRS and joining international organisations focused on promoting CV health as a policy priority such as the European Alliance for Cardiovascular Health.

Once the science behind the policy impact is ready high-level policy events can be organised such as seminars, workshops, policy briefings and conferences on a European, national, local scale. The power of the media and social media can also be harnessed to reach target stakeholders.

### Concluding remarks

Vascular ageing research outputs have the potential to have a lasting impact on society. Strategies for achieving healthy vascular ageing and preventing premature vascular ageing are both appealing and relevant to policy makers. Despite the challenges in this area, careful navigation of the political landscape and timely strategic communication will ensure that the high-quality science generated in the field will engage policy makers and inform practice and policy.

## Clinical applications

## 16. Cognitive decline and dementia

### Indra Steens^1^ and Thomas van Sloten^2^

^1^ Department of Internal Medicine, Maastricht University, Maastricht, The Netherlands

^2^ Department of Vascular Medicine, University Medical Center Utrecht, Utrecht, The Netherlands

### Status

Dementia is a highly common and devastating disease, often caused by a combination of different underlying pathologies. Efforts to treat dementia have not been very successful. Early prevention of cognitive decline is challenging when the risk factors and causes are not fully understood. Hypertension is associated with an increased risk of cognitive decline and dementia. EVA is increasingly recognized as an important contributor to the development of dementia and, thus, as a potential target for early preventive therapies. Observational studies have shown that a wide range of indicators of vascular ageing are associated with a higher risk of both vascular and other subtypes of dementia, including Alzheimer’s disease (Bos *et al* 2018, Deal *et al* 2019, Vasan *et al* 2022). These indicators include higher carotid artery and aortic stiffness and various markers of microvascular dysfunction and cerebral small vessel disease such as retinal microvascular parameters, white matter hyperintensities, cerebral microbleeds and lacunes. A better understanding of how vascular factors can contribute to the development of dementia is critical for identifying potential targets for preventive therapies.

A leading hypothesis is that arterial stiffness contributes to dementia via functional disorders or damage of the microcirculation. Arterial stiffness leads to an increased pulsatile pressure and flow load forwarded to the microcirculation of specific organs such as retina, kidney and brain. The microcirculation in the brain is particularly vulnerable for this increased load, as it is characterized by low impedance, allowing the pulsatile load to penetrate deeply into its microvascular bed without dampening the pulsatility by the windkessel function of the large and middle size arteries. Microvascular damage in the brain presents as white matter hyperintensities, cerebral microbleeds and lacunes. These remodelling processes can subsequently lead to cognitive impairment and dementia (Ainsworth and Rhodes 1986). However, epidemiological data suggest that the potential impact of arterial stiffness on dementia may not be entirely through measurable microvascular dysfunction. For example, a recent study showed that microvascular dysfunction accounted for only 16% of the association between aortic stiffness and worse cognitive functioning. The researchers used a composite score based on various measures, including plasma biomarkers, retinal measures and markers of cerebral small vessel disease (Rensma *et al* 2020).

**Figure 18.**
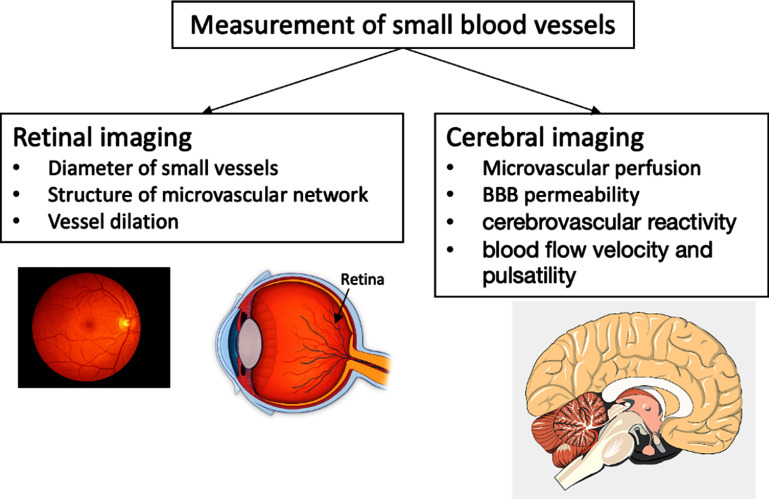
Methods to phenotype the smallest blood vessels in the retina and brain. Retinal microvasculature may be a good proxy for cerebral microvasculature.

Further studies focusing on independent risk factors and interaction between arterial stiffness, microvascular dysfunction and other pathologies such as inflammation and processes leading to neurodegeneration, are needed to understand the process leading to dementia. Recent advancements in imaging science now enable better phenotyping of the brain microvasculature and associated pathologies (see figure 18).

### Current and future challenges

Dementia is a heterogeneous disease that is often multifactorial. Currently, we do not know how EVA interacts with various other mechanisms, such as neurodegeneration and inflammation, that are thought to play a role in the development of cognitive impairment and dementia. Arterial stiffness may not only lead to increased pulsatile flow in the brain, but also contributes to inflammation, oxidative stress, and blood–brain barrier permeability, followed by neurodegeneration in regions needed for cognitive processing, thus contributing to cognitive decline and subsequently dementia (Iulita *et al* 2018).

Further research is needed to investigate the potential interaction between these factors in their contribution to the development of cognitive impairment and dementia. Additional factors that may interact with EVA in the association with dementia are IR, high levels of advanced glycation end-products, and glucose toxicity. In addition to investigating the downstream factors of vascular ageing, research should also focus on the role of the drivers of EVA (i.e. diabetes, hypertension, ageing, obesity) and their interaction with the above-mentioned processes, and whether currently available therapies are able to prevent these processes. Research is also needed to evaluate whether any of these processes are different according to sex. Due to the long preclinical phase of dementia, it is important to study the effect of these processes during midlife (or earlier in life) and evaluate the effect on cognitive performance in late-life (i.e. a life course approach).

### Advances in science and technology to meet challenges

Developments have been made in the field of MRI imaging with techniques that are able to assess the cerebral vasculature with high resolution. Examples are 7 T MRI that enables visualisation of the smallest cerebral vessels themselves and allows quantification of cerebrovascular reactivity, blood flow velocity and pulsatility at the level of the microvasculature (Park *et al* 2018). In addition, techniques are now available to quantify blood–brain barrier permeability (e.g. dynamic contrast-enhanced MR perfusion) (Canjels *et al* 2021) and microvascular perfusion (e.g. intravoxel incoherent motion). Intravoxel incoherent motion imaging may be able to distinguish between microvascular perfusion and parenchymal diffusivity, thus providing information on tissue microcirculation and blood flow as well as tissue microstructure simultaneously (Wong *et al* 2017). Another example is the combination of ultrafast, high-resolution US and contrast agents. This technique may visualize the cerebral microvasculature at high resolution in humans, providing both morphological and hemodynamic information (Demene *et al* 2021).

However, most of these MRI imaging techniques are not suitable for large studies, because the techniques are expensive and time-consuming. Alternatively, the retina provides a unique window on the cerebral microvasculature. There are many similarities between the retina and brain vasculature, including embryological development, physiology and anatomy (Kashani *et al* 2021). Retinal imaging is non-invasive, less expensive and less time-consuming than MRI imaging.

Retinal imaging techniques that can be used to assess the microvasculature include optical coherence tomography angiography which can visualize the microvasculature in the retina; fundus photography and adaptive optics which can assess static retinal parameters (including arterial and venular diameters, tortuosity, and fractals); and the dynamic vessel analyser which can assess dynamic retinal parameters (the vasodilation response, i.e. the increase in arterial and venular diameter upon flicker-light stimulation) (Li *et al* 2020). Additionally, retinal imaging techniques can be used to assess neurodegeneration and study the early pathobiology of dementia. Optical coherence tomography can be used to assess the thickness of the retina and its individual layers, which can be used as a proxy for cerebral neurodegeneration as thinning of the retina is thought to correlate with degeneration in the brain. This also allows for investigation of the interaction between early neurodegeneration and microvasculature damage as underlying mechanisms of dementia.

### Concluding remarks

Vascular ageing is thought to contribute to the pathology of different types of dementia. A better understanding of how vascular factors that contribute to the development of dementia is critical for the prevention of this disease. Future research in the field should include sex disaggregated data analysis and include methods to measure gender aspects in future studies if appropriate. Moreover, focus on the interaction of arterial stiffness and microvascular dysfunction with other mechanisms underlying dementia, including neurodegeneration and inflammation, and the drivers of vascular ageing, such as diabetes, hypertension, ageing, obesity, using a lifetime approach. Novel techniques are now available that can measure microvascular function. Recent MRI techniques allow direct imaging of the smallest vessels in the brain. However, most of these techniques are not easily applied in large cohort studies. Retinal imaging may provide a non-invasive, less expensive, and less time-consuming technique for imaging of the microvasculature that can be used in large clinical trials.

## 17. Heart failure

### Thomas Weber^1^, Siegfried Wassertheurer^2^ and Stefan Orter^2^

^1^ Cardiology Department, Klinikum Wels-Grieskirchen, Austria

^2^ Center for Health and Bioresources, Medical Signal Analysis, AIT Austrian Institute of Technology, Vienna, Austria

### Status

Heart failure is a growing problem, due to the ageing population worldwide. The condition is associated with impaired quality of life, repeated hospitalizations (challenging already highly stressed healthcare systems) and premature death, despite impressive improvements in treatment during the last decades (Mcdonagh *et al* 2021). Current guidelines recommend a distinction between heart failure with preserved (EF ⩾ 50%; heart failure with preserved ejection fraction (HFpEF)), mildly reduced (EF 41%–49%; HFmrEF) and reduced (EF ⩽ 40%; HFrEF) ejection fraction (EF) (Mcdonagh *et al* 2021). Although recently new treatments (e.g. SGLT2 antagonists) show benefits across the entire EF spectrum, leading some experts to a proposal to skip the EF-based classification, the bulk of medical and device-based treatments have proven benefits only in the HFrEF category. In Western-type and developed countries, arterial hypertension and coronary artery disease are the predominant causes of HF. vascular ageing has been recently identified not only as a consequence, but also as a precursor of arterial hypertension. Moreover, disturbed pulsatile hemodynamics (arterial stiffness, central hemodynamics, wave reflections, which can be expressed as premature or EVA (Climie *et al* 2023) have been shown to be closely related to every single step in the development and progression of CAD (Kim and Weber 2021). Therefore, vascular ageing is closely related to the two most important causes of HF, and measures of vascular ageing might be helpful for different aspects of HF management.

### Current and future challenges

It is obvious, that HF prevention programs are urgently needed to reduce the burden of the disease. Given the evidence outlined above, programs aiming for detection of vascular ageing, followed by adequate treatment, are ideally suited for this purpose and might ultimately reduce the burden of HF.

Major gaps in evidence in HF, outlined in the latest HF Guidelines of the European Society of Cardiology (Mcdonagh *et al* 2021), include, among others: (a) better phenotyping and understanding of the pathophysiology of HFpEF, (b) studies on the role of biomarkers in the diagnosis of HF and in screening of asymptomatic patients with HF, (c) validated diagnostic protocols for the diagnosis of HFpEF and HFmrEF, and (d) identification of novel treatments particularly for HFpEF. With respect to (a) and (c), in a recent scientific statement in the Journal of the American College of Cardiology (Borborlaug *et al* 2023), it was emphasized that the incidence and prevalence of HFpEF continue to rise in tandem with the increasing age and burdens of obesity, sedentariness, and cardiometabolic disorders. HFpEF remains under-recognized in everyday practice, and careful, pathophysiological-based phenotyping to improve patient characterization and to better individualize treatment plays a major role (Borborlaug *et al* 2023). Regarding (b), although the role of laboratory testing for natriuretic peptides has simplified the diagnosis of HF in general medicine (Mcdonagh *et al* 2021), this test is not easily available in all countries, and cannot be used for screening of asymptomatic patients.

### Advances in science and technology to meet challenges

**1. Screening for vascular ageing:** Similar to screening projects for hypertension, low-threshold services for screening for vascular ageing have been performed successfully (Danninger *et al* 2019), for instance on public spaces or in pharmacies. For screening, simplified measurement techniques or even estimations of vascular ageing, embedded in brachial BP cuffs, may be sufficient, given that further detailed examinations will be performed in case of abnormal results. The use of ML and AI, however, may allow better vascular ageing estimations from single-site waveform recordings (Jin *et al* 2021).

**2. Pathophysiology and phenotyping of HFpEF:** Arterial hypertension is present in the vast majority (in many studies > 90%) of HFpEF patients, accompanied by increased vascular ageing (increased arterial stiffness, premature/enhanced wave reflections). Due to ventriculo-arterial coupling, increased vascular ageing is closely related to left ventricular late systolic afterload, ventricular remodelling, diastolic dysfunction, exercise capacity, and, in the long-term, the risk of AF and new-onset HF (Weber and Chirinos 2018). In the next step, increased wave reflections may also represent a suitable therapeutic target in HFpEF (Weber and Chirinos 2018, Weber 2020).

**3. Diagnosis of HFpEF:** Reflecting the complexity of the pathophysiology of HFpEF, making the correct diagnosis/exclusion is not easy, and with the use of diagnostic scores, many patients with unexplained exertional dyspnea may end up in a diagnostic grey zone. At least for the HFpEF subtype with increased afterload and hypertension, markers of vascular ageing and pulsatile hemodynamics (including arterial stiffness, wave reflections and central pressures) may simplify the diagnostic process (Weber *et al* 2013). In fact, it has been shown that, based on ROC analysis, the diagnostic ability to separate HFpEF patients from non-HFpEF individuals among those with unexplained exertional dyspnea, is the same for the most commonly used echocardiographic parameter *E*/*E*′ and for most measures of vascular ageing/pulsatile hemodynamics (Weber *et al* 2013). However, the optimal way to add vascular ageing information to existing diagnosis scores of HFpEF is not yet known.

**Figure 19.**
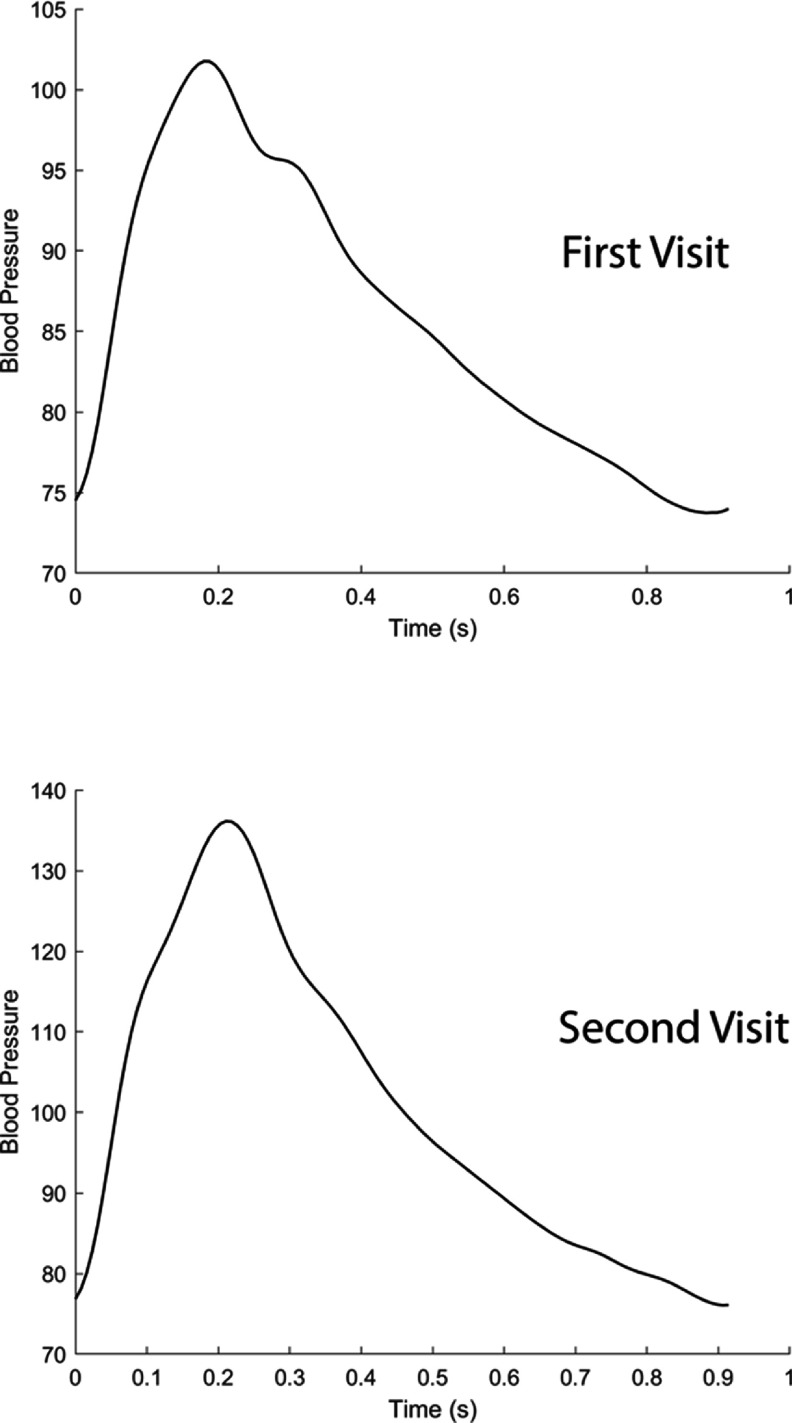
Changes in aortic waveforms in a patient with heart failure with reduced ejection fraction, from treatment initiation to the 6 month visit. Within this time period, ejection duration increased from 0.252 to 0.288 s, Augmentation Index increased from 26.1 to 35.1, and S to D ratio increased from 1.457 to 1.761.

**4. Early diagnosis of HFrEF:** In patients with HFrEF, the weak left ventricle cannot overcome the additional afterload from reflected waves, and ejection is terminated prematurely. The corresponding changes in pressure waveforms can be detected with the use of non-invasive tonometry, and can discriminate severely reduced from normal EF (Parragh *et al* 2015)—figure 19. For quantification of the changes, several indices can be used, including ejection duration (ED), augmentation index (AIx; derived from PWA), and S to D ratio (SDR; derived from wave intensity analysis). In a study in 50 children and adolescents, using a ML method based on intrinsic frequencies from carotid pressure waveforms (Cheng *et al* 2023), abnormal LV EF (<50%) could be detected with a accuracy of 92%. In a pilot study, using a slightly different approach, dedicated waveform features, assessed with PPG and accelerometry, were used to differentiate patients with heart failure from controls successfully with an area under the curve of 0.80 (Shah *et al* 2020). We envision a broad use, for instance by implementing discriminating algorithms into regular BP cuffs or wearables, which would allow wide-spread screening for HFrEF.

### Concluding remarks

We envision that assessment of vascular ageing can have an important role in the prevention and the diagnostic workup of HF. In addition, pathophysiologically-oriented treatments may play a role, given the close relationships between vascular ageing and HF.

## 18. Peripheral arterial disease (PAD)

### John Allen^1,2^ and Gerard Stansby^2,3^

^1^ Research Centre for Intelligent Healthcare, Coventry University, Coventry CV1 5RW, United Kingdom

^2^ Faculty of Medical Sciences, Newcastle University, Newcastle upon Tyne NE2 4HH, United Kingdom

^3^ Northern Vascular Centre, Freeman Hospital, Newcastle upon Tyne, NE7 7DN, United Kingdom

### Status

PAD affecting the legs is common and a marker of widespread atherosclerosis. PAD of increasing severity progressively leads to exercise-induced pain (intermittent claudication), and if more severe potentially to rest pain, gangrene and amputation. PAD is also relevant to the field of vascular ageing since disease prevalence increases with age, with symptomatic PAD affecting about 5% of people over 55 years old. PAD is also associated with an increased risk (i.e. x6) of heart disease or stroke and is also an increasing problem in diabetics, with an increasing number of leg amputations (Fowkes *et al* 2017). It is, therefore, important to establish the exact diagnosis for leg pain in middle aged and older subjects, since other conditions, such as musculoskeletal or venous disease, can mimic the symptoms of PAD. Most PAD patients are initially asymptomatic and so there is also a need not just for diagnosis but also for screening. Early disease detection can allow atherosclerotic risk factors to be controlled before potentially fatal CV complications occur. Additionally, PAD assessment technologies could be also used for monitoring patients who have undergone interventions, such as bypass grafting or stenting. Patients with abdominal aortic aneurysm (AAA) also share vascular risk factors with PAD patients, the early detection and treatment of these factors and premature vascular ageing would likely reduce incidence of/increase age at development of an AAA.

Standard objective PAD testing methods include BP measurements (e.g. ankle brachial pressure index [ABPI], toe brachial pressure index) and/or duplex vascular US (DVU) or imaging with contrast computerised tomography (CT) or magnetic resonance angiography (MRA). There are also pulse techniques under development such as PPG and impedance plethysmography (IPG). In the UK the ABPI has been recommended for primary care but is time-consuming and not all healthcare professionals have been trained in the technique. CT and MRA imaging are usually only available in hospitals and specialist clinical centres. DVU could be in principle be carried out in primary care but needs a trained operator. Recent NICE guidance recommends treatment for early-stage PAD which can be delivered by general practitioners (GPs), but despite the move to get care closer to home and for patients to be assessed outside of specialist centres, screening assessments for PAD are seldom carried out except in the private healthcare sector. New technologies are needed to overcome the barriers of high cost, test accessibility and risks from ionising radiation as well as enhanced care pathways to be introduced for the benefit of patients and for the healthy subjects where PAD can be quickly ruled out, ideally in primary care. With recent technological advances including wearable sensors and AI analytics there is great scope to develop existing PAD assessment techniques to make them more accessible, to deskill the processes involved in measurement and diagnosis, and to reduce cost burden to healthcare systems globally.

### Current and future challenges

**(A)Medical technology:**

**Figure 20.**
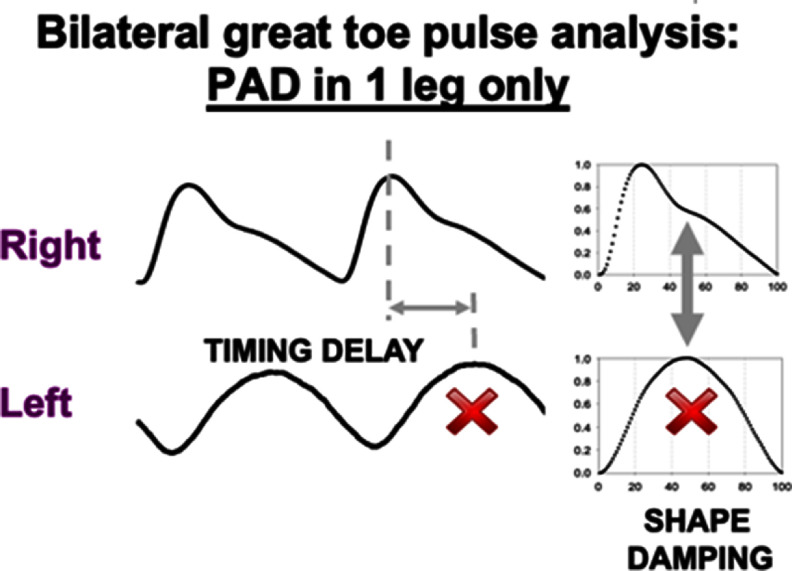
PPG has potential as an accessible, low-cost and fast method of PAD detection. The toe PPG trace for the leg with PAD is clearly damped and delayed compared to the leg that has normal arteries.

• BP-based measurements, e.g. ABPI, give quantitative measures for PAD diagnosis as well as gauging disease severity. Conventional manual ABPI measurement requires skill although the equipment required is relatively cheap and widely available (hand-held Doppler probe and manual sphygmomanometer). Automatic ‘oscillometric’ pressure measurement methods are being introduced but the literature on efficacy and optimal thresholds needs establishing. There is also reduced ABPI reliability with calcified leg* (e.g. in diabetes/renal disease) which can mask the presence of PAD. Instead, toe systolic BPs can be used (giving toe systolic pressure index) although methods/diagnostic thresholds lack standardisation.
• DVU imaging can give excellent visualisation of major peripheral arteries along with identifying raised blood velocities across a stenosis. Imaging in areas of blood vessel calcification can be suboptimal but usually a series of images and measurements are taken from which disease significance can be assessed, including degree of stenosis. Various PAD grading/reporting schemes are available, including the recent holistic system by Huthart *et al* (2022). A challenge would be semi-automating the image interpretation. Detection of AAA requires Imaging, most commonly with US scanning but improved resolution and greater portability of devices are needed for screening.
• MRA can be used to visualise the peripheral arteries, for example to identify stenosis, occlusion or aneurysm (Leiner and Carr 2019). Approaches tend to be classified as flow-dependent (e.g. ‘phase contrast’) and flow-independent. The most frequently applied MRA methods have involved the use of intravenous contrast enhancing agents, particularly those containing gadolinium. MRA requires significant skill to interpret/report but semi-automation of the process could support a practitioner.
• CTA is an x-ray tomography technique to image the blood vessels (Leiner and Carr 2019). It was initially limited by the use of single slice scanners that image a specific section of the arterial tree at a time but with the advent of multidetector row (e.g. 64-detectors) there is wider scope with contrast material injection to obtain the information in a single acquisition. The smaller distal arteries can be visualized. There are challenges in imaging blood vessels with dense calcification as this can have high attenuation of the x-rays leading to over-estimation of stenosis. Amongst potential side effects including radiation exposure, CT contrast is potentially nephrotoxic and is relatively contraindicated in those with poor renal function and additionally image quality can be poor in the presence of vessel calcification/metallic implant.
• Plethysmography techniques have shown promise for PAD detection. PPG is an apparently simple optical technique which shines light onto tissue and measures the pulsatile reflected light with each heartbeat. Research has shown that the toe PPG pulse usually becomes damped and delayed with the development of progressive PAD (Allen 2007, Kyriacou and Allen 2021a) (figure 20). There is also IPG which uses a high frequency alternating current stimulus applied to electrodes on the study limb alongside a set of receiving electrodes to measure impedance change with each heart beat (Haapala *et al* 2021). There are opportunities for PPG (and IPG) but currently there is little standardisation in methods and technology applied. PPG (and IPG) have considerable advantages, i.e. low-cost, ease-of-use needing only minimal training, portability (wearables capability) and the signals offer composite macro- and micro-vascular information. The PPG waveform, however, is not fully understood and there are challenges with its interpretation and classification. PPG measurements can be prone to high levels of artefact, e.g. from sensor—tissue movement artefact or in patients with tremor.
• Techniques to assess tissue perfusion and its impairment with PAD (Ma *et al* 2022), include transcutaneous partial pressure of oxygen (tcPO2) using an electro-chemical sensor (‘Clark-electrode’) method or optically using a fluorescence sensing method; spectrophotometry-based methods that can assess the tissue oxygen saturation to very low levels for example by measuring the shape of the visible light absorption spectra in the green region of the spectrum. There are protocol issues for these techniques, for example the optical techniques can be impacted by the degree of melanin in tissue, and diagnostic thresholds can be device (manufacturer) specific.
• As well as the above medical device technologies there are also blood biomarker testing and genetic screening opportunities for identifying those likely to have PAD and/or CV risk (Klarin *et al* 2021). Giving great scope for personalised medicine although with implementation challenges, e.g. ethical considerations and the taking of blood samples and their storage.

**(B) How best to identify undiagnosed PAD:**

PAD is a marker for more generalised atherosclerosis. PAD prevalence generally increases with age (e.g. in UK estimated at 20% at age 65 years, with about a third of these being symptomatic) (Criqui and Aboyans 2015). In PAD it is important to note that a 50% or greater stenosis in the peripheral arteries would be regarded as diagnostically important, i.e. as a marker of vascular risk, but probably a stenosis has to reach 70%–80% to become haemodynamically significant and cause significant symptoms (Udoff *et al* 1979). Also, that many older patients do not walk sufficiently to provoke claudication. As such the ‘pressure’ based techniques such as ABPI may be less ‘accurate’ in diagnosing the asymptomatic cohort.

Routine PAD screening is not usually performed in the NHS or most developed countries and people do not tend to go to their doctors unless they are significantly symptomatic, leaving many older people undiagnosed. There are many challenges, however, to offering routine PAD screening, including an appropriate assessment technology and the resources including suitably skilled staff to deliver this. Diagnostic thresholds would need to be established and careful targeting would also be undertaken to reduce the risk and health care costs and unnecessary patient anxiety of an excessive false positive rate and follow-on in the wider care pathway. There are approaches to identifying undiagnosed PAD that could be considered such as opportunistic screening and the use of new technologies such as Wearable Sensing and AI analytics.

**(C) Translation of new PAD technologies and techniques into clinical practice:**

With clinical validation of the device/technique sufficient studies, including prospective assessments, are needed through the formal pathway and ultimately undertaking a Health Technology and Innovation assessment that covers test efficacy, safety and health economics. The costs and cost effectiveness of PAD diagnostics will have to be demonstrated to justify use of and investment in new diagnostic technologies. There is, however, no perfect reference test for determining haemodynamically significant PAD, and the ones established in the literature do not always agree as they measure different aspects of the peripheral blood flow, e.g. ABPI pressures vs DVU imaging. Furthermore, there is no standard reference test stipulated for formal diagnostic test accuracy studies and usually there is no reference standard for the reporting of test results.

Medical device development also now includes proving safety testing and designing with usability in mind. Incorporation of co-creation techniques is essential for these aspects, including all relevant stakeholders in the design process. Knowing what the clinical practitioner requires for a PAD test is very important. Devices should ideally be developed for an appropriate level of automation and minimal training requirements and to be suitable for use by a wide range of clinical staff in both primary and secondary care. Furthermore, denoising of physiological data, i.e. signals and/or images and minimising false positives and negatives is important for real-world device solutions.

### Advances in science and technology to meet challenges

**(A)Medical technology:**

**Figure 21.**
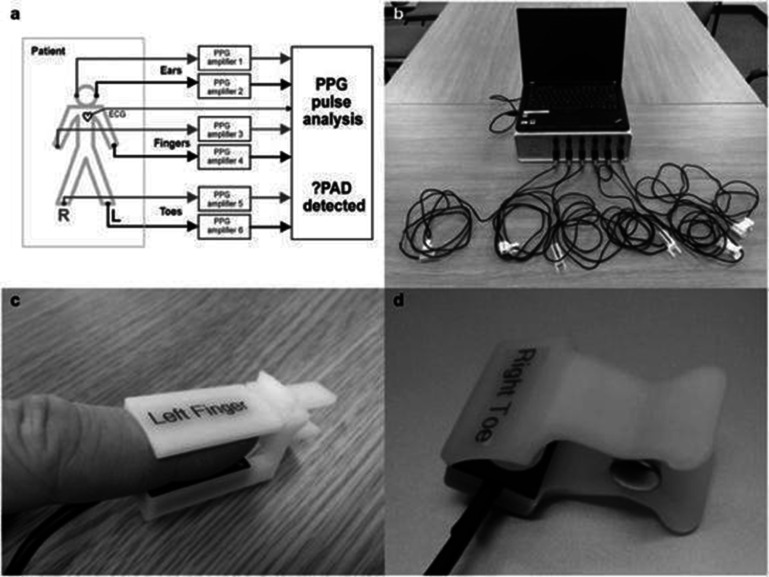
Example of a prototype PAD assessment device development offering a low-cost and easy-to-do PAD test for primary care and which is based on the optical pulse technique of photoplethysmography (PPG). The multi-site PPG system concept, PPG probes and main processing unit are shown (Stansby *et al* 2022). There is great scope for the miniaturisation of such technology. Reproduced with permission from Stansby *et al* (2022). Copyright © 2022 by SAGE Publications.

• BP-based measurements, e.g. the ABPI. Automated ‘oscillometric’ methods are being introduced and using parallel inflated limb cuffs which can make a rapid ABPI assessment. An automated approach should also help reduce subjectivity (‘operator independent’) in measurements. The system would apply a threshold test which indicates PAD based on the evidence base generated from earlier clinical trials. The problem of rigid leg arteries will likely remain a challenge with pressure-based measurement systems although there is potentially scope for an AI-analytics approach to consider the morphology of the oscillometric pulse waveforms to detect and compensate for calcified blood vessels. ABPI type assessment utilising toe BPs is also now possible.
• Imaging. DVU: Scope to semi-automate the image interpretation, including AI analytics to support the practitioner for PAD as well as AAA assessments. MRA: There have been many advances in acquisition speed, processing techniques and high-field MR systems such as 7 T field strength. Images, however, require significant skill to interpret and report, but as with DVU there are technological opportunities with AI for supporting this role.
• CTA: There have been advances in post-processing techniques including 3D reconstruction. Dual-energy CTA post-processing to subtract voxels containing calcium in the vessel wall is a promising technique for evaluation of calcified vessels. Images require significant skill to interpret and report, there are technological opportunities with AI for this role.
• PPG continues to show promise for PAD assessment. There are still unknowns about what the technique actually measures but the pattern of pulse damping and delay in the majority of cases of significant disease has been shown in multi-site PPG studies for hospital and primary care settings. Its ease of use, low cost and portability are enablers for the technology to assess vascular disease (e.g. www.isrctn.com/ISRCTN13301188). PPG has the advantage of miniaturisation using advanced microelectronics and sensors, with new multi-site systems being developed for CV assessments, including for PAD (Stansby *et al* 2022) (figure 21) and vascular ageing (Charlton *et al* 2022b). Advances continue in analytics include signal denoising, CV modelling and data synthesis, arrhythmia detection, AI-classification of diseases including PAD with the potential power of explainable AI, new insights into vascular ageing, and the novel communication of disease (Kyriacou and Allen 2021a). There is scope for further innovation and for bespoke devices for use in primary care to bring care closer to the patient (Allen and Hedley 2019). Bespoke wearable PPG sensing offers accessible testing and with advanced analytics in the cloud that are possible. With IPG there is also great scope here for PAD testing and incorporating analytic approaches similar to PPG, IPG however has the potential advantage compared to optical techniques of not being influenced by the amount of melanin in skin (‘skin tone’). PPG and IPG could be combined.

**(B) How best to identify undiagnosed PAD:**

There are new digital health technologies for opportunistic screening e.g. accelerometer and walking patterns revealing abnormal gait linked to intermittent claudication. These could also be tailored to better inform the public in vascular diseases including PAD, and to improve lifestyles including diet, smoking and exercise. Diabetic patients, who are at growing risk for lower limb amputation, need accessible PAD screening in the care pathway. There is also self-testing (e.g. using a mobile phone with PPG sensor to take a pulse recording for analysis). Improved analytics with de-noising will be needed for a host of ambulatory assessments. There can be the secondary analysis of MRI/CT scans which were obtained for other purposes. Genetics/blood marker testing has a future role as one of a number of novel approaches to help ensure that those at greatest PAD risk can receive appropriate health checks. A further important consideration is for the screening for PAD in countries having strongly resource-constrained settings, noting that a single type of PAD assessment technology may not suit all settings worldwide. It is also vital that the signal/image algorithms are developed and tested for a range of clinical settings (e.g. hospital vascular setting vs. primary care). It is possible that different PPG features best suit specific settings/disease prevalence.

**(C) Translation of new technologies and techniques into clinical practice:**

There are many challenges along the innovation pathway from an initial idea for a PAD assessment technology to clinical validation and introduction into a care pathway. Many facets of research should be undertaken including device design through co-creation with all key stakeholders, full compliance with medical device regulations (area covered elsewhere in Roadmap), clinical validation (qualitative and quantitative research), safety, robustness to artefact, device usability, health economics, and commissioning research to align although potentially reconfigure a care pathway. As well as efficacy the costs and cost effectiveness need to be demonstrated to justify the use of and investment in new diagnostic technologies. An international perspective should also be considered, for example applicability to lower-income countries and the requirements for technologies to be used in resource-constrained settings, including a consideration of the resources needed to treat such patients.

### Concluding remarks

The clinical need for the timely and accessible detection of PAD has been highlighted. There are some clear limitations with the current technologies for initial patient assessments, as many are hospital based and require highly skilled staff to assess and report the test. There are though newer technologies coming through plethysmography techniques, particularly PPG and IPG, showing promise for accessible and low-cost first stage PAD assessments—these can be implemented in digital healthcare devices and utilise miniaturised sensors including wearables and advanced analytics including AI and Cloud-based approaches. PPG has already been explored for the assessment of general vascular ageing. There is work across the board to do though and there are many challenges with the introduction of new technologies into existing/new care pathways.

## 19. Challenges in vascular aging and atrial fibrillation

### Giacomo Pucci^1,2^, Andrea Grillo^3^ and Davide Agnoletti^4,5,6^

^1^ Department of Medicine and Surgery, University of Perugia, Perugia, Italy

^2^ Unit of Internal Medicine, ‘Santa Maria’ Terni Hospital, Terni, Italy

^3^ Department of Medical, Surgical and Health Sciences, University of Trieste, Italy

^4^ Department of Medical and Surgical Sciences, University of Bologna, Bologna, Italy

^5^ IRCCS Azienda Ospedaliero-Universitaria di Bologna Policlinico Sant’Orsola, Bologna, Italy

^6^ Cardiovascular Medicine Unit, IRCCS Azienda Ospedaliero-Universitaria di Bologna, 40138 Bologna, Italy

### Status

The worldwide incidence and prevalence of atrial fibrillation (AF) are increasing, and AF is the most common cardiac arrhythmia. The prevalence of AF increased three-fold over the last 50 years, and its burden on global cumulative mortality showed an 81% relative increase during the past 20 years (Lippi *et al* 2021). The exacerbation of AF prevalence is associated with an increasing prevalence of AF risk factors, such as advancing age, hypertension, obesity, diabetes, heart failure, ischemic heart disease and kidney disease.

From a pathophysiological point of view, AF could be conceived as the result of an accelerated vascular ageing. When a subject is exposed to progressive stiffening of the arterial tree, for any reason, the central pulse pressure rises together with the left ventricular afterload. This eventually leads to atrial remodelling and exposes the subject to an increased risk of AF. It follows that worsening of arterial stiffness may precede AF development (Cremer *et al* 2015). Therefore, it seems worth noting that identifying subjects with accelerated vascular aging (VA) could enable a stricter follow up strategy, in order to detect AF early. Besides, increased arterial stiffness, as a marker of vascular ageing, and AF show similar risk factors such as hypertension, diabetes, coronary heart disease, or kidney disease. This is also witnessed by the evidence that increased arterial stiffness is more frequently observed in patients with than without AF (Pauklin *et al* 2021).

The development of new algorithms for rhythm monitoring contributed to the implementation of AF detection by the new smart portable devices (e.g. smartphones and wearables), increasing the chance of opportunistic AF detection (Perez *et al* 2019). Prompt diagnosis of AF is pivotal to initiate the therapeutic management and reduce the global AF burden. Besides, the availability of tools for early identification of individuals at high risk of developing AF will lead to more appropriate screening programs in the general population and to preventive campaigns targeted to selected individuals.

Ideally, the simultaneous assessment of BP, arterial stiffness and AF by a single device would be of extreme usefulness in clinical practice, and at the same time is a challenge from a theoretical and practical point of view.

### Current and future challenges

Given the close relationship between AF and vascular ageing, the implementation of algorithms for AF detection into devices that measure vascular ageing is a desirable goal. Increasing the screening rate of the arrhythmia may potentially reduce the disease burden and be cost-effective, especially in elderly individuals with hypertension and other risk factors. Nevertheless, the large number of devices purporting to measure vascular ageing does not permit a single strategy to diagnose AF.

**Figure 22.**
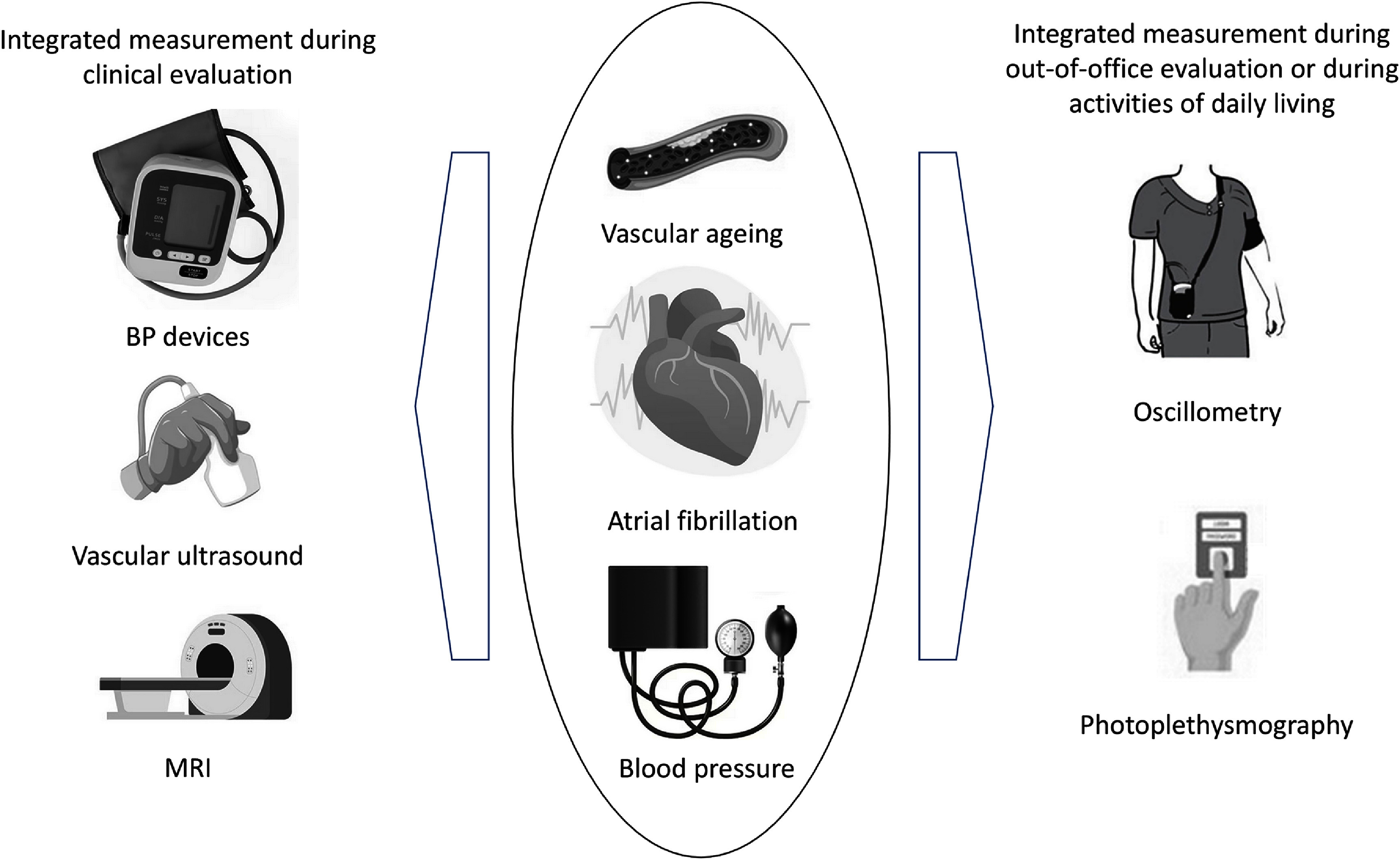
Schematic representation of an ‘ideal’ device allowing simultaneous and integrated measurement of blood pressure, vascular ageing and AF screening, and potential areas of application into devices currently used for clinical (left) or out-of-office (right) evaluation based on the analysis of biological signals recorded during activities of daily living.

The preferred screening approach may be by devices offering a high-quality ECG trace integrated in their equipment (some arterial tonometry, echocardiography, US of carotid arteries, cardiac MRI). The automated detection of AF from single-lead ECG is currently based on proprietary algorithms which identify the absence of P waves and RR irregularity, and marketed on wearable or handheld AF screening devices (figure 22).

Devices using oscillometric techniques may also contribute to AF screening. Blood pressure (BP) monitors which detect AF from oscillometry-based algorithms have been in the market for a few years, with very high sensitivity and specificity rates, ranging from 90% to 100% (Park *et al* 2019). The algorithm for diagnosis, based on pulse irregularity, could be implemented in devices measuring central BP or PWV from oscillometric cuffs.

Ambulatory blood pressure monitor (ABPM) devices, traditionally used in clinical practice to evaluate the BP behaviour over the 24 h period and under dynamic conditions, are particularly promising on this purpose. Some marketed devices already combine measurement of arterial stiffness parameters, such as PWV and central BP, with peripheral BP measurement over 24 h (Papaioannou *et al* 2013). Other ABPMs have been equipped with algorithms for automated AF detection during each automated oscillometric BP measurement (Kollias *et al* 2018).

Even if the choice of implementing ABPM with vascular ageing measures could be questionable for a large-scale application, due to its intrinsic tolerability issues, its added value in terms of accuracy and circadian assessment is not negligible. By contrast, technologies based on smartwatches/smartphone applications, while undoubtedly more tolerated and widely available, lack accuracy in vascular assessment.

Combining these technologies into a single device could not only prolong the window of AF detection over the 24 h period, but also enable simultaneous measurement of BP and arterial stiffness, together with AF detection, during conditions known to increase the risk of paroxysmal AF, such as stressful periods, with the potential to unveil novel pathophysiological mechanisms.

All these three clinical conditions are frequently undiagnosed even for very long periods because of their asymptotic behaviour. According to the WHO, an estimated 46% of adults with hypertension are unaware that they have the condition, and undiagnosed AF accounts for 20% of all AF episodes and for 3%–6% of all ischemic strokes (Liao *et al* 2007).

### Advances in science and technology to meet challenges

Any chance to screen for the presence of elevated BP, AF and accelerated vascular ageing should be regarded as an opportunity to identify individuals at very high cardiovascular (CV) risk. The possibility of detecting AF from PPG techniques is very appealing and largely tested on a variety of wearable devices, ranging from smartwatches, fingertips and smartphones (Pereira *et al* 2020). Advances are warranted in improving the accuracy of testing. Machine and deep learning algorithms may offer the possibility of reaching a clinical grade reliability of screening for these tools, which may be also included in vascular ageing measurement devices based on PPG.

AF detection, either interpreted by the physician or automatically performed by an algorithm, could become of routine use, although specific medical recommendations are lacking for implementing detection of AF in devices not designed for this purpose. The detection rate of asymptomatic or symptomatic AF could be improved both in the general and in the high-risk population if appropriate algorithms are implemented on vascular ageing measurement devices.

Another major scientific issue, which is not adequately answered by current literature, is the accuracy of vascular ageing measures in patients with established AF. Indeed, the irregularity of heart rate could influence the measurement of vascular ageing indices. This would depend on both the technique used and the baseline hemodynamic status. Tonometric PWV and Doppler based techniques seem to be unaffected by heart rate irregularity (Halfon *et al* 2018). BP oscillometric devices (ODs) are suitable for BP measurement in AF at least in presence of normal ventricular rate ranges, while the use of oscillometry may be flawed in AF for measurement of certain indices, such as ABI (Dąbrowski *et al* 2021). Further research is needed, considering the high prevalence of this arrhythmia in the population tested with these devices, for both establishing the reliability of each vascular ageing measurement tool and improving the signal quality with pulse irregularity, in order to provide a clear recommendation for the clinical use.

A long-time perspective for vascular ageing assessment is the development of a device that reliably measures BP, vascular ageing and detects AF at the same time. To date, however, there is no device available on the market combining all these technologies into a single device, and this remains an unmet need.

Oscillometry and PPG are considered the most promising techniques in this regard as they may be able to expand the opportunities to assess arterial stiffness and BP levels under dynamic conditions, such as by wearing a PPG-based device or an oscillometric ABPM. This might increase the chance to evaluate which parameter of arterial stiffness would better predict clinically relevant outcomes over the short and long term. However, the ability to accurately measure BP with PPG-based cuffless devices has been questioned and their use should be discouraged until further data and developments become available (Mukkamala *et al* 2023).

These and other challenges await developing technologies on devices allowing simultaneous multiparametric assessment of CV health markers.

### Concluding remarks

AF, elevated BP and accelerated vascular ageing, from a pathophysiological and clinical perspective, are strongly linked to each other. Given that a considerable proportion of patients are undiagnosed, further technological developments could help increase the screening and detection rate of these conditions. Screening technologies need to be accurate enough to reduce the risks of overdiagnosis and overtreatment, and accessible enough to be applied to high-risk populations without added risks and costs. Which is the most effective screening approach, and which could be the potential outcome from combining different screening approaches into one single device, are two important questions that should be answered in the future.

### Acknowledgment

This work was supported by COST Action CA18216 VascAgeNet, supported by COST (European Cooperation in Science and Technology, www.cost.eu).

## Future research directions

## 20. Young people

### Rachel E Climie^1^ and Vimarsha Kodithuwakku^1^

^1^ Menzies Institute for Medical Research, University of Tasmania, Hobart, Australia

### Status

Although overt CVD may not appear until later in life, the risk factors for CVD begin to develop in early life. Importantly, recently published findings now demonstrate a direct association between childhood CV risk factors and adult CV events and mortality (Jacobs *et al* Jr 2022). This is significant given the high prevalence of childhood CV risk factors globally. For example, in 2016, approximately 81% of adolescents worldwide (aged 11–17 years, 85% of girls, 78% of boys) were not meeting physical activity recommendations (60 min of moderate to vigorous intensity physical activity per day) (Guthold *et al* 2020). Childhood obesity has become overwhelmingly challenging, with simulated growth trajectories predicting 57% of today’s children will be obese by the time they are aged 35 years (Ward *et al* 2017) and likely as a consequence, global rates of hypertension in children and adolescents have increased by 75% from 2000 to 2015 (Song *et al* 2019).

Exposure to CV risk factors as early as during foetal life, promotes the development and accumulation of subclinical vascular changes that direct an individual towards a trajectory of EVA (Climie *et al* 2021). Indeed, the atherosclerotic process begins in early life, with an accumulation of lipid in the intima of arteries (fatty streaks) observed in most children. Postmortem studies show that this phenomenon is accelerated in the presence of traditional CV risk factors (McGill 1990). The arteriosclerosis process also commences at a young age and is influenced by risk factors such as obesity, BP and young onset type 2 diabetes. Importantly, EVA in early life is associated with subclinical disease in adulthood. Ample evidence shows that elevated BP in young people (children and adolescents) is associated with CV adaptations both cross-sectionally (Kollias *et al* 2014) and longitudinally (Juhola *et al* 2013). Children with stiff arteries (>95th percentile) have accompanying increased left ventricular mass (Urbina *et al* 2011) and reduced left ventricular function (Mehta *et al* 2018), compared to children with more favourable stiffness measures. In childhood, increased IMT is associated with increased left ventricular mass (Sorof *et al* 2003) and poorer cardiac function (Mehta *et al* 2018). Furthermore, central BP appears to be more tightly associated with CV dysfunction than brachial BP in young people (Peluso *et al* 2017). Thus, given the aforementioned worsening of health in young people globally, it is likely that increasing numbers of children and adolescents will display EVA, compared to their healthy age and sex matched counterparts, putting them at increased risk of CVD later in life. Characterising vascular ageing from youth may, therefore, provide a window into CV risk later in life (Climie *et al* 2021). While there are several methods that could be used to assess vascular age in young people, there are limitations to these, as summarised below. There are also a number of research challenges that need to be addressed.

### Current and future challenges

Before vascular age can be routinely measured in clinical practice in young people, a number of research questions need to be answered. Firstly, there is a lack of consensus in general in terms of how to define EVA and this also applies to young people, where growth and development also need to be considered. Secondly, to accommodate the perfusion needs of the developing body, vascular wall and lumen dimensions change as the child grows. However, it is currently unknown what is ‘normal’, or healthy, vascular ageing (i.e. development of the vasculature) and what is pathological, or how this differs between boys and girls, as reference values for vascular ageing in young people are currently not available. Thirdly, although it appears that EVA in early life is associated with subclinical outcomes, more data with longer term follow up is required. In line with this, it is unclear how different markers of vascular age are differently associated with cardiometabolic risk factors in young people as no direct head-to-head comparison has been performed. Furthermore, there are inconsistencies in studies examining the impact of intervening on vascular age in young people, due to differences in the types and lengths of interventions, methodologies employed to quantify the intervention, the vascular ageing biomarker examined or the health and age of the study population. Finally, there appears to be a lack of awareness regarding the potential benefit of measuring vascular age in paediatric populations among clinicians, however, this still needs to be definitively confirmed. Taken together, it remains unclear which vascular ageing biomarker/s should be the focus for technological advances. In table 2 we summarise the most common techniques for measuring vascular ageing biomarkers in clinical practice and their advantages and challenges related to use in young people.

**Table 2.** Techniques for measuring vascular ageing biomarkers in clinical practice and their advantages and challenges related to use in young people.

Technique	Method of measurement	Insights provided on vascular ageing	Current use in young people	Advantages of measuring in young people	Challenges to measuring in young people
Intima media thickness (IMT)	Ultrasound (B mode or radio-frequency ultrasound)	Large artery structure (Measured manually, semi-automatic or automatic)	Widely used in clinical settings for adults Frequently used for research purposes in both adults and young people	• Non-invasive	• Values may differ due to different ultrasound settings, the intima media edge detection algorithms, and off-line reading systems (Drole *et al* 2020) • Require technique- specific reference intervals

Carotid to femoral pulse wave velocity (PWV)	Oscillometric technique Piezo-electric method High-resolution ultrasound technique + echo-tracking system	Arterial stiffness	Frequently used in research settings	• Non-invasive • Validated in children and adolescent (Kracht *et al* 2011)	• Time consuming • Standardisation between different techniques and devices required in order to compare values • Require technique- specific reference intervals • Tolerability in young people

Distensibility and stiffness index	MRI/ultrasound + oscillometric method (BP)	Arterial stiffness at a single vascular site (e.g. carotid artery)	Mostly used for research purpose Not commonly used in clinical settings	• Non-invasive	• Requires a local pressure measurement for the calculation and this can be influenced by human error and appropriateness of the cuff

Central blood pressure	Direct intra-arterial measurement—Invasive technique (Intra-arterial catheters)	Blood pressure, waveform	In surgical procedures and intensive care Not commonly measured in children	• Constant monitoring of blood pressure in hemodynamically unstable patients • Waveform graph can be obtained • High accuracy of the blood pressure measurement	• Invasive • Time consuming
	Cuff-based device	Blood pressure, waveform	Commonly used in clinical and research settings	• Non-invasive • Waveform graph can be obtained	• Measurements can be impacted by the difficulty of finding appropriately sized cuffs for young people
Peripheral blood pressure	Korotkoff sound method (manual) Oscillometric method (automatic)	Surrogate of ventricular afterload	Most widely used in clinical and research settings	• Non-invasive	• Less precise due to the difficulty of finding appropriately sized cuffs for young people • High observer bias if using manual method • White coat effect is frequent among children (Jurko *et al* 2016).

Augmentation index and reflection magnitude	Applanation tonometry Automated oscillometry	Arterial function (quantifies wave reflection)	Used in both clinical and research settings	• Non-invasive	• Low agreement and consistency between the different devices • Central augmentation index and reflection magnitude may be poorly estimated by brachial oscillometric devices in young people (Mynard *et al* 2018).

Flow mediated dilation	Ultrasound (High-resolution B-mode)	Endothelium-dependent vasodilatory reserve of muscular conduit arteries	Used in both clinical and research settings	• Non-invasive • Specific reference intervals are available for children.	• Influenced by cuff placement

Reactive hyperemia-peripheral arterial tonometry (RH-PAT—digital)	Plethysmographic technique—EndoPAT device	Endothelium-dependent vasodilatory reserve of peripheral arterial bed	Not commonly used in either clinical or research settings	• Non-invasive	• Difficult to perform in young people due to discomfort due to upper arm compression • No specific devices for young people

### Advances in science and technology to meet challenges

There are a number of advances in technology that may aid in the routine measurement of vascular age in young people. Firstly, the development of a tool or device that is efficient, user friendly, tolerable by young people, validated, cost effective and proven to be associated with outcomes (cardiometabolic risk, subclinical CVD) would significantly advance the use. One of the key factors to consider in development of such a tool is the growth and development of the child. For example, consideration of how to account for children of different heights, heart rates and pubertal status which may influence the vascular age value. It is also important to minimise any operator interference required to improve precision and accuracy. As an example, many US systems now include an automated analysis package to ascertain vascular ageing measurements such as carotid IMT. These automated systems have proven efficiency over manual measuring techniques. However, reliability of these measurements, specifically related to young people, has not been widely explored and would be of value for the advancement of such technology (Shenouda *et al* 2018).

PPG-based devices are an advanced technique that could be considered as a more convenient approach to assess vascular ageing but are not widely used to measure vascular ageing among children. Even though this technique is used in RH-PAT, it can be uncomfortable for young people (Mueller *et al* 2017). However, PPG could potentially be developed and adopted as a convenient modality such as mobile apps/smart phones/children-friendly/portable devices to assess vascular ageing. Furthermore, ML methods have been a significant advancement in science and technology which may be applied to the measurement of vascular ageing in young people (Bikia *et al* 2021a). ML techniques are now being developed to assess other vascular ageing measurements such as PWV in routine care (Bikia *et al* 2021a). These techniques could be further developed and validated in young people, to facilitate regular use in clinical settings and for research purposes.

The establishment of reference intervals for young people for the various vascular ageing biomarkers is also required. The Youth Vascular Consortium (Fong *et al* 2021) is a collaborative effort involving more than 40 research groups worldwide and it is hoped that reference intervals for vascular ageing will be the result of the ongoing work of the Consortium.

### Concluding remarks

Characterising vascular ageing in young people will provide a window into CV risk later in life. The development of a tool or device that is efficient, user friendly, tolerable by young people, validated and cost efficient, would significantly advance the field and aid in the routine measurement of vascular age. However, a number of research questions remain regarding vascular ageing in young people that need to be addressed, perhaps alongside the development of such technology. If grounded in scientific evidence, this could make a profound impact on the number of today’s young people expected to develop CVD in future.

### Acknowledgments

R E C is supported by the National Health and Medical Research Council of Australia (reference: 2009005) and by a National Heart Foundation Future Leader Fellowship (reference: 105636).

## 21. Using Machine Learning in vascular aging assessment

### Vasiliki Bikia

Stanford University, Stanford, California, United States

Swiss Federal Institute of Technology of Lausanne, Lausanne, Switzerland

### Status

Machine Learnin (ML) is a subset of AI—defined as computers’ ability to emulate intelligence—that provides systems with the capacity to learn automatically from data without explicit human input. In the realm of ML, two fundamental paradigms, supervised and unsupervised learning, play distinct roles. Supervised learning involves training models on labelled datasets, enabling algorithms to map inputs to their corresponding outputs. In contrast, unsupervised learning deals with unlabelled data, allowing algorithms to explore underlying patterns or structures without predefined outputs. In vascular medicine, physicians have traditionally focused on synthesizing various observations to identify diagnostic patterns for guiding treatment and patient management. Sophisticated ML models can offer significant support to clinical experts in some of these tasks.

**Figure 23.**
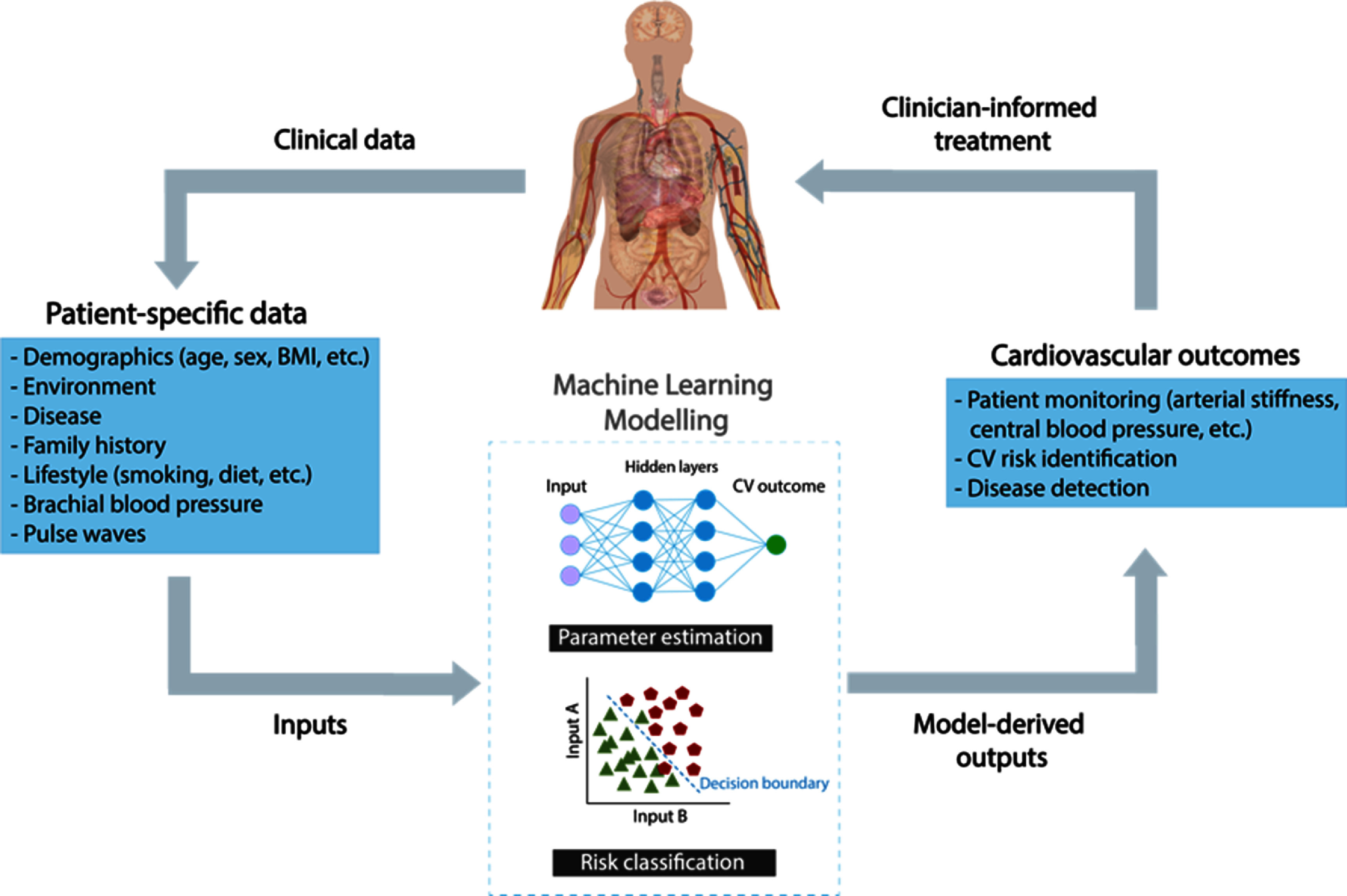
Using machine learning to assess vascular ageing from readily available clinical data. Adapted from Bikia *et al* (2021a). CC BY 4.0.

Learning from data through traditional statistical approaches (such as linear regression) has long been a part of vascular medicine. With recent advancements in ML models and the increased availability of data, there is growing potential to tackle some of the most challenging problems. In vascular aging (VA) assessment, ML is applied via two main types of models: parameter estimation models and risk classification models (figure 23) (Bikia *et al* 2021a). Parameter estimation models, for instance, can estimate a target parameter from more easily obtained measurements, such as estimating (invasive) central hemodynamic from non-invasive peripheral BP data (Bikia *et al* 2020). Risk classification models, on the other hand, might classify a subject according to their risk of a particular outcome, such as estimation the risk of a cardiovascular (CV) event event from pulse wave velocity (PWV), risk factors, and laboratory data (Garcia-Carretero *et al* 2019). While vascular ageing is primarily evaluated using classical algorithms (e.g. decision trees or support vector machines), there is a noticeable shift towards the adoption of deep learning algorithms (Bikia *et al* 2021b); a class of ML algorithms which allow for evaluating large datasets without the need for predetermined assumptions, by using multiple-layer structures to extract higher-level information from the raw data.

ML may unlock a variety of possibilities in the assessment of vascular ageing. Currently, patient management in primary care is mainly guided by BP measurement, as a number of issues limit the use of other markers of vascular age. ML-based techniques could offer a streamlined addition to primary care with minimal additional workload, such as using easily obtained clinical data to estimate central pressure or assessing vascular age from PPG pulse wave. Furthermore, these ML techniques may even have the potential to outperform traditional statistical modelling techniques in many clinical scenarios. Additional benefits of ML tools could include: (i) the analysis of data from electronic health records to provide clinical decision support and automated insights through the effective handling and analysis of data of multifaceted nature and high dimensionality; or (ii) the application of deep learning on large datasets collected through multiple registries that could improve risk stratification and enable precise long-term risk prediction.

### Current and future challenges

While healthcare systems collect vast amounts of data, vascular medicine carries distinct challenges unlike other data-driven industries where ML has thrived. Large datasets are essential to develop and validate ML-based techniques. For ML to achieve adequate performance in a clinical context, it requires training data that are not only large in volume but also diverse and reproducible. Often, datasets only record traditional risk factors as binary variables; thus, relevant features may not be captured, or lack the granularity and volume required for ML to effectively uncover relevant relationships (London 2022).

Furthermore, PW data are not routinely recorded; US methods, albeit well-established in the clinical monitoring, are not typically employed to generate large datasets of pulse wave; other PW acquisition techniques, such as applanation tonometry, are not widely adopted in the clinical setting; and, finally, although PPG-based wearables can monitor the PW effectively, accessing or storing the raw data from these devices is often challenging. In addition, although several devices for acquiring arterial pulse wave in the clinic may output data in a format suitable for analysis, they usually require a skilled operator and are not widely used for collecting datasets for research.

Uncoordinated data assembly and sharing practices can result in two major issues: the need for extensive data curation to prepare datasets for building ML models, and, more importantly, can result in models resulting in inaccurate outcomes and potentially false medical decisions. A recent review highlighted shortcomings in the methodology used to develop clinical prediction models using ML (Christodoulou *et al* 2019). A common challenge in applying data-driven algorithms for vascular health assessment and the main limitation to their application to real life clinical situations (Zech *et al* 2018), is overfitting. Overfitting occurs when an ML model learns rules that perform very well on the training data but underperform on new, unseen data (test data), leading to models that are not generalizable. To prevent biased results, it is crucial to ensure that the same data instances are not used in both the train-set and test-set.

Despite evidence suggesting that ML algorithms may improve accuracy of predicting clinical outcomes (Kakadiaris *et al* 2018), the lack of interpretability of ML models has often been considered as a limitation for the use of ML in clinical applications. This is attributed to the fact that the rules by which ML models have achieved their performance are not clear, given the high complexity of the neural networks or other mathematical structures. This lack of easy interpretability of the models’ decision-making means that it can be difficult to verify whether the learned rules have indeed generalized to real life clinical situations (Prakash and Tucker 2018).

### Advances in science and technology to meet challenges

Benchmark datasets could play a key role in meeting the need for large and high-quality datasets and provide a standardised approach for developing and testing ML-based techniques to assess vascular age. These datasets act as a reference ‘standard’ for a model to be evaluated against. They should contain data reflective of the target population and ideally contain a wide range of characteristics to allow the strengths and weaknesses of ML-based techniques to be assessed. Future research efforts should consider the establishment of a registry containing data with relevant markers of vascular ageing, that has both adequate sample size and is reflective of the target population. Concurrently, it is of great importance to ensure appropriate reporting of ML-based methods, so that it is feasible to verify the absence of overfitting or methodological misconduct. In conjunction to clear and concise reporting of the methods, further quality assessment through external validation is critical for quality assurance (Dung-Hung *et al* 2022).

Moreover, focus should be directed to those measuring techniques that can provide access to valuable clinical information from PW data, such as applanation tonometry. In this respect, PW monitoring techniques should be standardised to be suitable and effective for generating large datasets, which can then be used for training and testing ML-enabled parameter estimators and/or risk classifiers. In addition, consumer devices that obtain PW signals can be used as an alternative to the measurements derived in the clinic. For mobile and wearable technology integration, advancements should prioritize the establishment of methodological concepts and protocols to ensure that digital data is not only easily accessible but also stored and managed effectively on cloud platforms. Emphasis must be placed on maintaining high data quality standards and ensuring interoperability (namely the possibility that data can be integrated and used together with other types of data) across diverse systems. data can be integrated and used together with other types of data). Especially, a high level of interoperability is key to collect and exploit new and large digital datasets (Mandl *et al* 2024).

Research is ongoing to improve the interpretability of ML models, using innovative concepts, such as explainable ML and parallel models, where one is used for core computation and the other for interpretation (Barredo Arrieta *et al* 2020). An alternative approach could involve simulated data, generated from a computer simulator, that could significantly aid interpretability, as they are derived from deterministic models in which relationships between variables may be more easily explained (see section 9).

### Concluding remarks

ML presents high potential in developing new techniques to assess vascular ageing and enable risk stratification in vascular patients. When combined with effective interventions these new techniques could help reduce CV morbidity and mortality. The plethora of data routinely collected in healthcare settings and in daily life provides opportunity to identify individuals at risk and to monitor their vascular health. Yet, additional work is required to develop adequately validated and standardised ML-based methods. These methods should not only predict biomarkers and/or CV events accurately, but also identify the specific clinical scenarios in which their use is beneficial and cost-effective.

## 22. Sex and gender-based differences in vascular ageing

### Mai Tone Lønnebakken^1^ and Ute Seeland^2^

^1^ Department of Heart Disease, Haukeland University Hospital and Department of Clinical Science, University of Bergen, Bergen, Norway

^2^ Institute of Social Medicine, Epidemiology and Health Economics, Charitè—Universitätsmedizin Berlin, Humboldt-Universität zu Berlin, and Berlin Institute of Health, Berlin, Germany

### Status

Sex is the biological differences between women and men, while gender is the psychosocial differences leading to differences in roles, behaviours, exposures, expression and identity between women and men. Both sex and gender modify vascular ageing by influencing the gradual age-dependent changes in vascular structure and function that leads to increased arterial stiffness, arteriosclerosis, atherosclerosis, and CVD development (Seeland *et al* 2021). A meta-analysis demonstrates that EVA is associated with risk of CV events and mortality in both sexes (Ben-Shlomo *et al* 2014). However, EVA is more common in women than in men (Bruno *et al* 2020).

**Figure 24.**
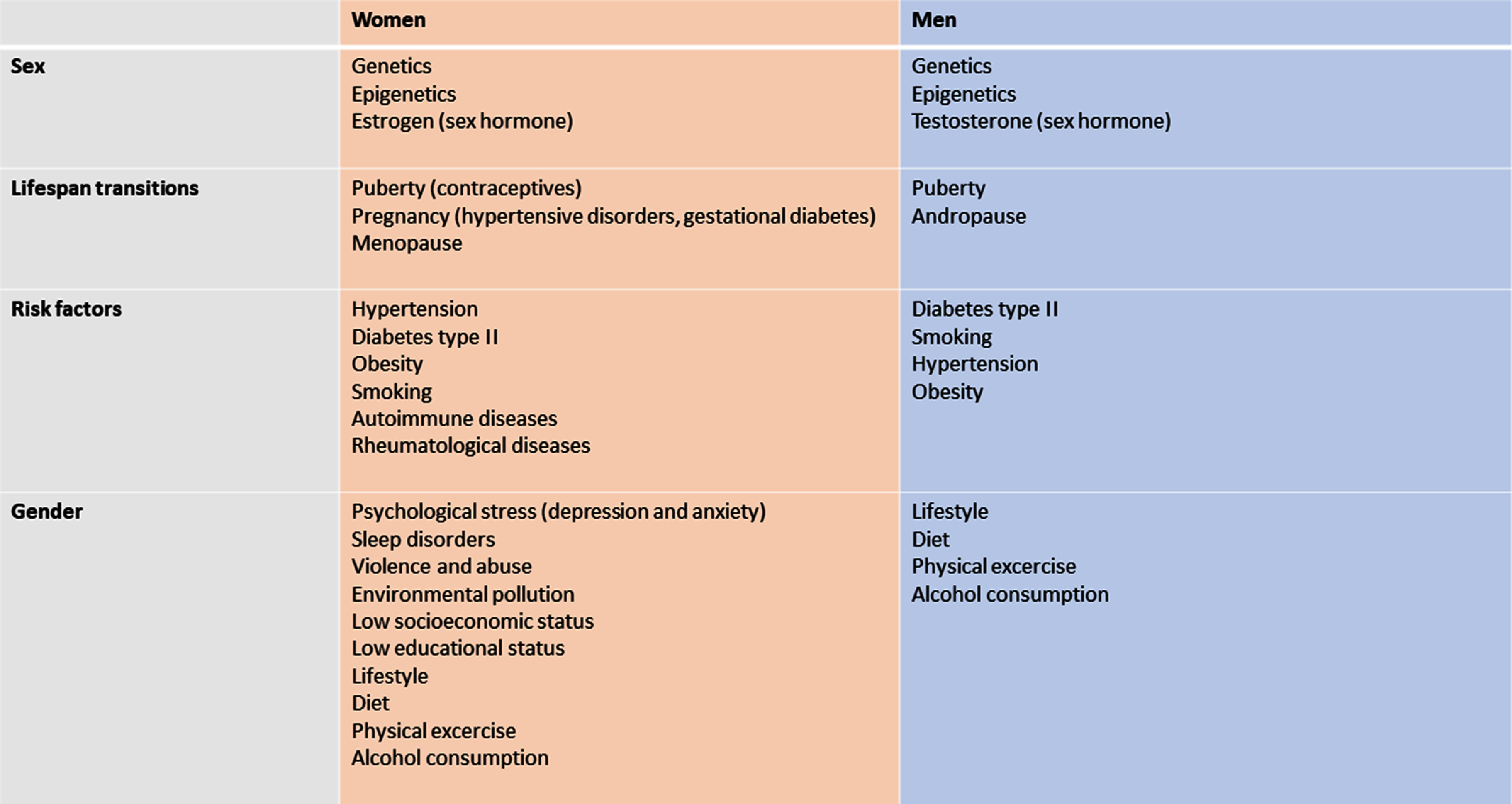
Factors contributing to vascular ageing in women and men separately.

Sexual dimorphism in gene expression (different genes and gene dosing based on XX and XY sex chromosomes), sex differences in epigenetic regulation and function, sex hormones and sex-specific lifespan transitions result in important sex differences in vascular physiology and pathophysiology (figure 24). Puberty, pregnancy, menopause and andropause, represent important lifespan transitions that impacts vascular ageing. In women, contraceptives, pregnancy associated hypertensive disorders, hormone disturbances, premature menopause and inflammatory autoimmune diseases have been demonstrated to be associated with increased arterial stiffness. CV risk factors like hypertension, diabetes, and obesity, accelerate vascular ageing differently in women and men. Furthermore, there are also sex differences in risk factor trajectories (how risk factors change with age). BP and arterial stiffness increase more rapidly with age in women than in men, and the predicted risk of CV events increases at lower BP values in women (Ji *et al* 2022). Likewise, diabetes induces more pronounced and greater age-related stiffening of the aorta in women compared to men (De Angelis *et al* 2004). These differences are further modified by gender-related factors, like sociocultural factors, environmental risk factor exposure, as well as availability and utilization of the healthcare system. In particular, psychological stress, violence and abuse, sleep deprivation, pollution, low socioeconomic and educational status are associated with accelerated vascular ageing and CVD in women (Seeland *et al* 2021, Vogel *et al* 2021).

**Figure 25.**
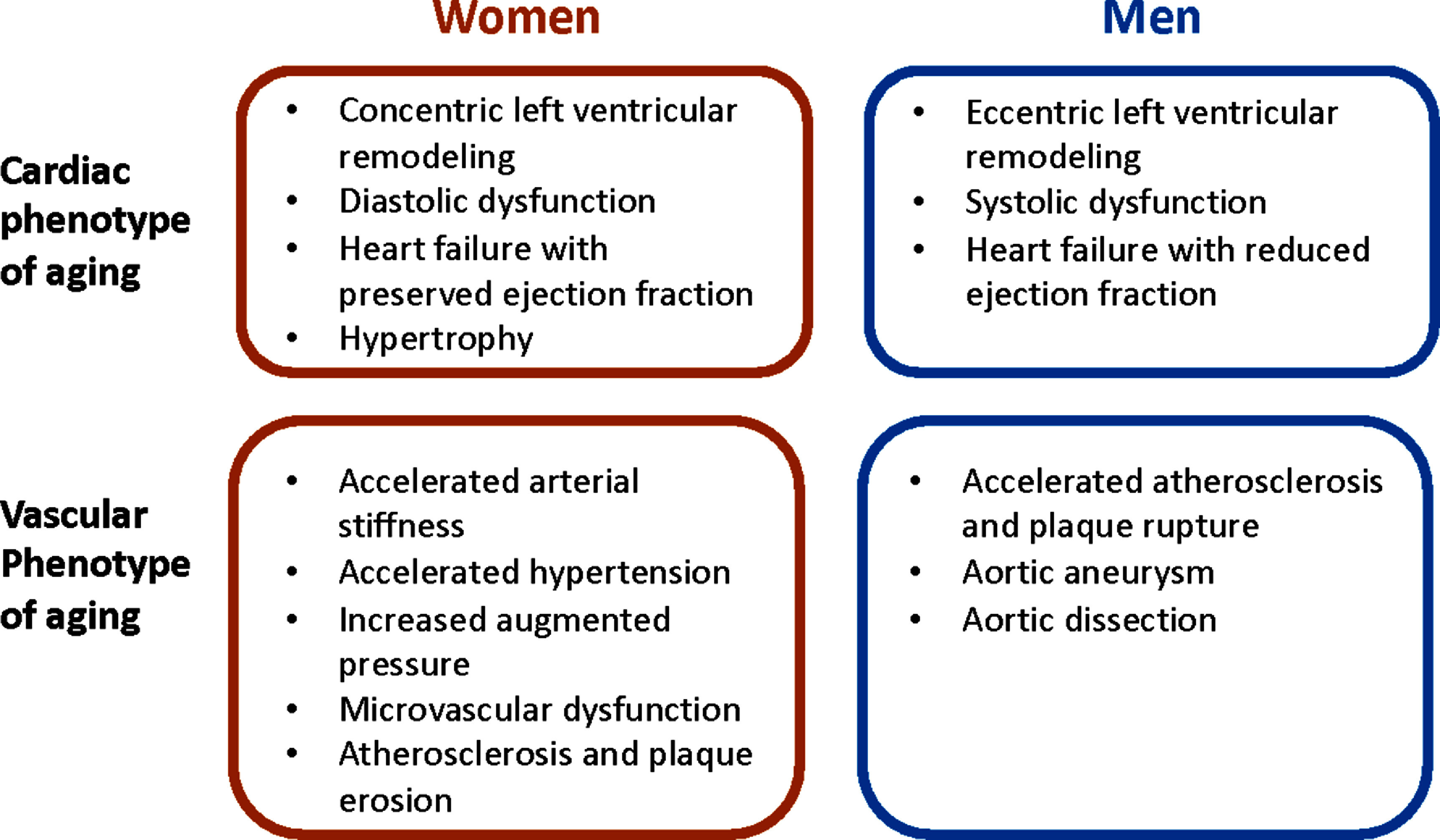
The sex-specific cardiovascular phenotype of ageing in women and men.

Sex differences in body size, fat distribution, CV anatomy and hemodynamics must be considered in assessment and interpretation of vascular ageing. However, anatomical differences due to weight and size are of secondary importance compared to the hemodynamic and functional CV differences due to genetic and hormonal modifiers of vascular ageing. Smaller aortic and arterial dimensions in women are balanced by smaller heart chambers, lower stroke volume and increased heart rate compared to men. However, the differing responses to risk factors by men and women result in the development of distinct sex-specific high-risk CV phenotypes (figure 25). Women exhibit accelerated arterial stiffening, more pronounced PW propagation, earlier wave reflection and higher augmented pressure (Seeland *et al* 2020, 2021). This suggests that both arterial stiffening and vascular tone, mediated through effects on smooth muscle cells in the arterial wall, contribute to cardio vascular ageing in women. A decline in endothelial NO-mediated vasodilation in women, in particularly in concomitant hypertension, also results in microvascular dysfunction and associated increased CV risk; while macrovascular accelerated atherosclerosis is more common in men.

Advances in the understanding of sex- and gender specific accelerated vascular ageing have the potential to improve detection of high-risk individuals, preclinical disease and disease mechanisms. The aim of this sex- and gender-sensitive medical approach is to offer the population a non-discriminatory, improved medical care and health prevention for all sexes.

### Current and future challenges

Cross-sectional studies have demonstrated that EVA is associated with poorer prognoses in mixed populations (Ben-Shlomo *et al* 2014). Prospective studies including sex-disaggregated data on the impact of EVA on the short- and long-term risk of CVD are still missing. Furthermore, age and sex-specific normal values for vascular structure and function, as well as sex-specific cut-off values associated with increased CV risk, are still to be established.

Current recommended CV risk score models underestimate risk and contribute to under treatment, particularly among women (Sedlak *et al* 2020). There is reason to believe that the inclusion of markers of EVA in current risk score models may add to CV risk prediction. In addition, including sex differences in lifespan transitions, risk factor and risk factors trajectories, sex-specific risk factors and gender-related factors in CV risk assessment may further improve risk stratification (Vogel *et al* 2021). Importantly, in clinical practise and research, gender-related factors known to contribute to accelerated vascular ageing are often ignored. The development of simple, user-friendly tools to assess gender-related risk factors has potential to add to CV risk stratification. Furthermore, current understanding of the underlying pathophysiological mechanisms resulting in EVA is spare and limits the possibility to target prevention and treatment against specific disease mechanisms.

Non-obstructive coronary artery disease and HFpEF represent key clinical challenges mainly affecting women. Sex differences in cardiac and vascular remodelling and dysfunction may be important in disease development. Non-obstructive coronary artery disease is associated with both myocardial ischemia and myocardial infarction, and this may be due to microvascular and/or vasospastic coronary artery disease. In patients with HFpEF, cardiac remodelling associated with hypertension and increased arterial stiffness, results in diastolic dysfunction and heart failure (Triposkiadis *et al* 2019). Whether intervention against accelerated vascular ageing may impact disease progression, reduce symptoms, and improve survival in these patients should be explored. Intervention to delay vascular ageing in women and men needs to be investigated through randomized trials demonstrating not only benefit on vascular structure and function but also on long-term outcome. It is not yet clear whether vascular ageing could be delayed by postmenopausal hormone therapy, new and sex-specific treatment targets, or more aggressive and early onset treatment with lifestyle intervention and antihypertensive medication at lower BP values.

### Advances in science and technology to meet challenges

Prospective studies powered to assess the separate prognostic impact of EVA in women and men, are needed to advance our current knowledge on sex differences in vascular ageing and implement this in clinical practise. Future big data analysis may also contribute to completing our understanding of the impact of sex and gender in vascular ageing. This may generate new hypotheses and contribute to the identification of sex-specific mechanisms of vascular ageing and potential sex-specific treatment targets. Specific treatment recommendations to prevent and delay vascular ageing and reduce CV risk in women and men must be established through randomized clinical trials. In particular, sex-specific indications, treatment targets and timing of therapy should be explored.

A comprehensive evaluation of both cardiac and vascular structure and function are necessary to detect preclinical and clinical vascular ageing. The current diagnostic procedures to detect signs of vascular ageing are time-consuming, lack standardisation, and are limited by availability. Technological development of new and user-friendly devices for easy and reliable assessment of vascular structure and function to improve CV risk assessment, detect subclinical vascular disease, and identify high-risk individuals, must be validated in both women and men (Qiu *et al* 2021). Furthermore, wearable devices controlled by smartphones may be used as a screening tool to detect high-risk individuals with EVA in the future. However, to diminish gender disparities, it is crucial that the devices are customized, available for both sexes, and include gender-specific questionnaires.

Deep learning and ML algorithms of indices of vascular ageing may improve the reproducibility, standardisation, and availability of vascular age assessment. Recently proposed basic ML models based on parameter estimation or risk classification to quickly and accurately assess vascular ageing have been suggested (Bikia *et al* 2021a). However, it is critically important that patient-specific input data also include sex-specific risk factors for vascular ageing and that validation cohorts include a significant number of both women and men to ensure that the algorithm is valid to assess vascular ageing in both sexes.

### Concluding remarks

The importance of sex and gender as modifiers of vascular ageing and CVD development is well documented. Future research and technological advances should focus on identification of clinical biomarkers and sex-specific vascular phenotypes associated with accelerated vascular ageing, as well as subclinical and clinical CVD. Risk score models implementing sex differences in vascular physiology, lifespan transitions, risk factors and risk factors trajectories, gender-related factors and validated sex-specific cut-off values of vascular structure and function may contribute to improving detection of accelerated vascular ageing. Accordingly, identifying high-risk CV phenotypes in combination with better knowledge of the underlying sex-specific disease mechanisms may contribute to more personalized and targeted prevention and treatment that may improve the prognosis and reduce the burden of CVD in both women and men.

## 23. Mathematical modelling for understanding of PWA

### Berend E Westerhof^ 1,2^

^1^ Department of Pulmonary Medicine, Amsterdam Cardiovascular Sciences, Amsterdam UMC, Vrije Universiteit Amsterdam, Amsterdam, The Netherlands

^2^ Department of Neonatology, Radboud University Medical Center, Radboud Institute for Health Sciences, Amalia Children’s Hospital, Nijmegen, The Netherlands

### Status

#### Waveform analysis

Waveform analysis is the area of research that aims to gain information about the cardiovascular system (CVS) by investigating features of arterial pressure (Nichols 2005), flow (Hashimoto *et al* 2018), or plethysmography waveforms (Charlton *et al* 2022b).

**Figure 26.**
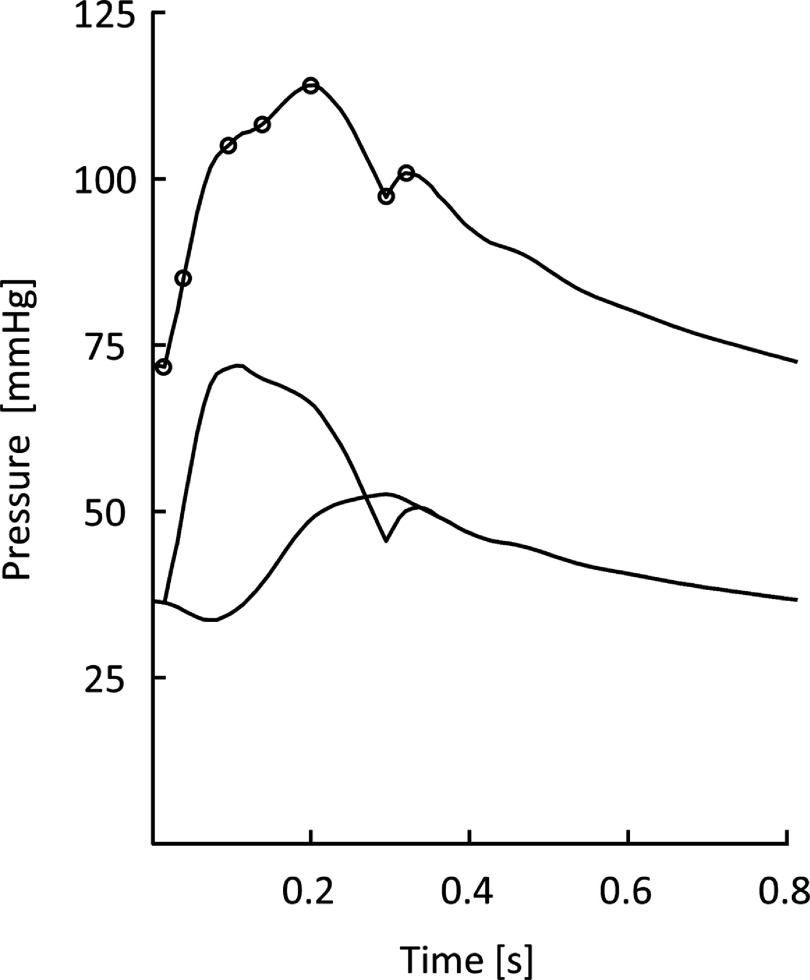
Parameters describing a pressure waveform, based on characteristic points on the pressure wave (upper curve). From left to right: start of upstroke and diastolic pressure, (Pdia), maximal rate of change (max-d*P*/d*t*), shoulder point (Psho), inflection point (Pinf), systolic pressure (Psys), incisura (Pinc), dicrotic wave (Pdic). The augmentation index AIx = [(Psys − Psho)/(Psys − Pdia)] · 100%. Wave separation provides the forward and reflected pressure waves (lower curves).

Several parameters to describe a wave shape exist (figure 26), with varying degrees of clinical relevance, and varying degrees of physiological underpinning. A well-known example is the augmentation index (AIx), quantifying the secondary rise in BP that is frequently seen in early systole (Nichols 2005). It has commonly been regarded as a measure of pressure wave reflection and arterial stiffness, and, accordingly, as a measure of vascular ageing. The AIx derives its acceptance as a useful surrogate end-point from studies where relations with clinical outcome were shown in specific patient groups. The case for the AIx is thus largely empirical, although the following simple physiological model has been suggested for its origins: a forward wave is generated by the ventricle, travels down the aorta, reflects at the aortic bifurcation, and the reflected wave travels back to the heart. When the aorta becomes stiffer with age or disease, PWV increases and the reflected wave arrives back at the heart sooner, increasing BP in systole.

#### Mathematical modelling

Mathematical modelling is the approach to describe (with formulae, e.g. physics-based differential equations) a system such that it produces the outputs that are measured in real life.

Many different forms of models are used in CV research. Zero-dimensional models describe variables over time but not in space, like the ‘Windkessel model’, relating pressure and flow at a certain point in the circulation (Alastruey *et al* 2023). One-dimensional models do take distance into account, for instance the single tube model to interpret wave reflection with a distinct reflection site, or for the calculation of wave speed. More complex one-dimensional models use a network of arteries to represent reality more closely (Alastruey *et al* 2023).

Models can assist in understanding of a certain system. The parameters of the system, for example the radii of the vasculature, or wall stiffness, can be easily changed in the model, and the effect on the BP waveform can be studied. Sometimes, the outputs do not entirely conform to reality, prompting rethinking of the assumptions that were made, and perhaps concluding another or more sophisticated model is needed.

When a sufficiently accurate model is used, and its output fits to the measurements, it can be assumed that the model has been correctly ‘parameterised’. Parameters may then be interpreted, e.g. the arterial stiffness of the vascular system.

Nowadays, measurements and waveform analyses are frequently performed, and many mathematical models, simple and complex, are available (Alastruey *et al* 2023). However, determining the parameters of the model so that the measurements are correctly reproduced, a process called ‘system identification’, is not widespread.

### Current and future challenges

In general, the challenge with modelling aimed at getting a better insight into the physiological basis underlying pulse wave, is to find the balance between obtaining a good description of reality versus system complexity, or number of parameters. Very simple models as for instance the Windkessel model may reproduce the global features such as systolic and diastolic pressures, but are not adequate to explain wave shape parameters like the AIx. Even the single tube model with a reflection site at the end is too simple to understand wave reflection in the aorta (Westerhof and Westerhof 2018). Reflections occur not at one main reflection site, but at many sites (Westerhof and Westerhof 2012), and reflections are not ‘complete’ as would be the case at a closed end, but partial and with a phase delay (Westerhof *et al* 2008). Comprehensive distributed models, incorporating these refinements, do explain actual findings, for instance the limited decrease of the return time of the reflected pressure wave with ageing (Baksi *et al* 2009, Westerhof and Westerhof 2012). A single tube model for the calculation of carotid-to-femoral PWV is obviously an oversimplification since the wave does not travel from the carotid artery to the femoral artery. Another parameter that is typically neglected is the radius of the tube in which PWV is determined. Radius may differ less between subjects than vascular stiffness, but mathematically they have equal impact on PWV.

Currently, there are several challenges that need to be addressed to advance the use of models underlying the generation of the arterial waveform. A first challenge, as introduced in the previous paragraph, is that comprehensive models are needed for the correct interpretation of arterial pulse wave. This comes with a second challenge: more comprehensive models have more parameters, and how can these be determined? Additional challenges are presented by the introduction of new devices and measurement techniques. Wearables can provide continuous waveform data, however, may be corrupted artefacts, e.g. caused by motion. Novel modelling techniques are needed to improve the signal quality and reliability. At present, PPG is used more frequently, but it is not related to arterial pressure in a straightforward manner and may require modelling to retrieve the underlying physiology.

It makes sense to first develop comprehensive models on a population level: the model is correct for the ‘average patient’. The main future challenge is the individualisation of a model to represent a particular patient. Such an individualised model is referred to as: ‘digital twin’.

### Advances in science to meet challenges

#### Comprehensive model

Many models developed for specific applications are already available. Combining these models to reach the required level of refinement could advance the successful use of models to explain arterial waveforms. Improvements can be achieved by taking in geometry of the vasculature into account with more detail. The distribution of stiffness may be seen as a part of geometry, the aorta being more compliant than conduit arteries. Stiffness increases with age and elastic vessels stiffen more with age than conduit vessels. Also, stiffness of all vessels is highly dependent on distending pressure, in a nonlinear manner. Then, there is the influence of ventricular function on the arterial wave shape, with heart rate and ventricular preload (Van De Velde *et al* 2018) major contributing factors. Cardiorespiratory interaction introduces a beat-to-beat variation in the waveform. Also, physical and mental stress, and autonomic function, have effects on the wave shape.

Models with more complexity need more measurements to determine the additional parameters. To better describe geometry of the vasculature, MRI imaging may be used. PWVs may be established over diverse trajectories and for different ages. Heart rate and respiration rate may be obtained from a simple ECG to improve hemodynamic modelling.

#### Individualisation/digital twin

Simple patient data such as age, sex, height and weight may help towards individualisation of a certain model. An ECG is easily obtained, but due to costs, MRI measurements to improve the geometry of a specific individual model are not feasible for all patients. Better descriptions of the evolution of parameters with ageing, and of deviations with pathologies may help improve models at population level, reducing the additional measurements needed for individualisation. Citizen science and the use of wearables may present a new avenue for the accumulation of large datasets.

ML can be deployed as a powerful tool for parameter estimation and system identification. With present day computing power, large datasets can be easily analysed. Currently, ML is mainly used to develop black box models helpful for prediction or classification, however, a system identification approach (white box model) would allow finding the affected ‘parameter’ related to pathology in a group and eventually in an individual.

A very interesting approach is to generate wave shapes with comprehensive models, in which parameters are varied over wide a range of physiological and even pathophysiological values. In this way, large populations of ‘virtual’ healthy subjects and ‘virtual’ patients can be created (Charlton *et al* 2019). Measured wave shapes could be matched to the waveshapes in the virtual database to identify the most likely underlying set of parameters, thus giving insight into the origins of the waveshape.

### Concluding remarks

Waveform analysis of arterial pressure, flow, or plethysmogram can reveal information about the CVS; mathematical modelling can be used to find the physiological basis of the observed waveform features. The used models should be sufficiently detailed for a correct description of the CVS; however, higher levels of complexity require more model parameters. The ultimate goal is the individualisation of models; a suitable digital twin will make it possible to predict clinical outcome and to virtually test the effect of therapeutic interventions. Where measurement and modelling meet, real knowledge of the CVS can be found.

## 24. Using multiple sensing modalities to assess vascular age

### Panicos A Kyriacou and Parmis Karimpour

Research Centre for Biomedical Engineering, City, University of London, London EC1V 0HB, United Kingdom

### Status

It is estimated that 7.6 million people are living with CVDs in the UK, and the annual healthcare costs are over £9 billion (British Heart Foundation UK Factsheet 2018, NHS England Long Term Plan: Cardiovascular Disease 2019). Blood vessels begin to deteriorate with age, a phenomenon known as vascular ageing. vascular ageing can cause changes to the structure and function of the blood vessels, increasing the likelihood of an individual developing a CVD. Regular monitoring of vascular health and cardiac function could reduce mortality, morbidity, and unplanned hospital admissions, in addition to improving exercise capacity, quality of life and psychological well-being. However, at present, there is no medical grade technology that can provide the precision, resolution, and patient comfort necessary to achieve regular, repeated, long-term monitoring of vascular health, particularly outside of a hospital environment.

Researchers over the years have been working on the utilisation of non-invasive sensing modalities for the assessment of vascular disease (Kramer 2020, Kyriacou and Allen 2021b, Charlton *et al* 2022b). Despite the very encouraging research outcomes from many studies there is still no reliable and clinically acceptable non-invasive sensor modality that is used routinely for the rapid assessment/screening of vascular ageing and disease.

Primarily the assessment of vascular ageing nowadays remains reliant on rather costly, impractical, and expert-dependent imaging modalities (diagnostic US, MRI) or other more invasive and resource intensive interventions such as angiography which can only be implemented in a clinical setting. Even though such technologies are currently available, in the UK patients must be referred by their GP to undergo such investigational procedures in a hospital setting which can cause significant delays and added anxiety to patients.

The development of reliable and accessible non-invasive sensor technologies that will allow for the early screening and diagnosis of vascular ageing at a GP level could bring about transformative solutions in the early assessment of CVD.

### Current and future challenges

One of the most common techniques that clinicians use for the assessment of vascular disease is the ABI test. This technique is the preferred first test for symptomatic patients, which could provide information relating to the location and extent of PAD. This test requires a Doppler US probe to locate the artery, and pressure cuffs for BP measurements in the upper and lower limbs. ABI is calculated by dividing the BP measured in an artery of the ankle by the BP measured in an artery of the arm. Normal calculated ABI values are between 0.9 and 1.4. An abnormal ABI below 0.9 could act as a strong indicator of CV risk.

While the ABI technique is non-invasive and relatively inexpensive, it is not very practical, and it requires a degree of expertise especially when using the Doppler US probe and hence it could be operator depended. If further tests are required, clinicians can refer the patients for further diagnostic US tests, an angiogram, or other imaging modalities.

Diagnostic US, an acoustical based imaging modality, is one of the main methods for assessing vascular age. It can provide arterial visualisation non-invasively, without radiation exposure. Measurements can be taken from deep tissue, asynchronously without the need for anaesthetics, and without interruption to blood flow circulation (Haschek *et al* 2010). However, diagnostic US techniques can be costly and more importantly require experts to perform the measurement/scanning. Vascular health can also be monitored with perhaps a less costly technique using a laser Doppler flowmeter, however this technique has been proven unfavourable as it can suffer from motion artefact and signal processing limitations (Obeid *et al* 1990).

Angiography is also popular for investigating vascular ageing. It provides the anatomical and structural images of the vascular system. A contrast agent is injected into the vessel and the vessel is projected on a series of x-rays, allowing the inner vessel wall and flow in the lumen to be visualised (Omeh and Shlofmitz 2022). Also, CT coronary angiography is a non-invasive technique, which accurately evaluates luminal and outer vessel wall dimensions, and classifies high-risk plaques. However, it suffers from radiation exposure and has a limited imaging resolution, causing difficulties in accurately specify between lipid and fibrotic tissue components (Mushenkova *et al* 2020). Also, other more advanced imaging modalities can be employed for the diagnosis of arterial disease, such as PET and MRI, however such modalities are very expensive, less practical, and patients can be impacted from radiation exposure (Kyriacou and Allen 2021b).

While the above technologies used for the assessment of vascular ageing are well established, there is a gap in technologies which could be utilised for the early diagnosis on vascular health especially at a GP level. Such technologies could enable prompt detection of pathologies relating to vascular ageing and prompt early-stage interventions. This could be accomplished by innovation on non-invasive sensor technologies which are currently growing exponentially in the field of health and wellbeing applications.

### Advances in science and technology to meet challenges

Over the years many researchers have attempted to develop sensor technologies to assess PAD and vascular ageing. These were primarily based on the optical technique of PPG (Kyriacou and Allen 2021b). PPG is a non-invasive optical technique widely used in the study and monitoring of the pulsations associated with changes in blood volume in a peripheral vascular bed. This technique is widely used in both medical devices, such as pulse oximetry where it is used for the non-invasive measurement of arterial blood oxygen saturation (SpO2), and in the majority of wearable technologies (bracelets, wrist watches, etc) where it is used to monitor various physiological parameters, including pulse and respiratory rate, relating to health and fitness.

The main driver of how PPG could be utilised for the assessment of vascular ageing is based on the hypothesis that PPG provides information relating to both changes in haemodynamics and vascular mechanics, both of which are directly related to vascular health. Such an approach seems very attractive for the non-invasive and rapid assessment of vascular ageing.

PPG can be used to assess PWV and PTT, both of which relate to vascular ageing. Such approaches utilise two PPG signals measured at different sites or one PPG and one ECG signal. More recently researchers have also developed PWA and ML models in an effort to identify features of PPG signals which are related to vascular health (Kyriacou and Allen 2021, Charlton *et al* 2022a). Despite the high volume of publications in this field, where promising findings are presented, there is still no available and clinically routinely used technology based on PPG for the assessment of vascular ageing (Alnaeb *et al* 2007).

While there is robust evidence that the PPG relates to changes in vascular ageing, there are still many barriers that limit the utilisation of PPG-based technologies to reach a commercial clinical device used for the routine assessment of vascular ageing.

These could be related to:

• Lack of standardisation of the PPG technology (optical-front-end). This included the selection of optical wavelengths used, optical component topology, and site(s) of application.
• Lack of standardisation of the PPG technology, analogue-front-end. This includes the electronics needed for the optimal acquisition of PPG signals.
• Lack of standardisation of the PPG signals processing and analysis. This could include:
  * PPG signal processing (pre-processing, motion artefact removal, signal quality assessment, calculating derivatives, decomposition)
  * Methods for feature-based PPG signals analysis (Fiducial point detection, Feature extraction)
  * Methods for PPG time and frequency domain analysis
  * Optimal and relevant PPG ML models used for vascular ageing assessment.
• Lack of standardisation of signal acquisition protocols used for the assessment of vascular ageing. Authors have engaged in diverse studies in an effort to unravel the relation of PPG and vascular ageing. These include *in vivo* studies on healthy volunteers, small groups of patients, utilisation of simulated PPGs, utilisation of diverse existing databases, including *in vitro* studies utilising PPG phantom technologies. Such approaches have failed to secure the correct protocol or approach needed for such an assessment.

All of the above challenges have resulted in diverse and sometimes inconclusive results. There is clearly a need for a more systematic approach of how best we can use PPG for the assessment of vascular ageing.

Another potential approach of sensors technologies used for the assessment of vascular ageing which has not be considered, at least in detail, is a multi-sensor approach. While we know that PPG has clear relations with vascular ageing, using PPG alone might not be the only or the best solution. The addition of other sensors in combination with PPG might be a more attractive option. A multimodal approach could perhaps mitigate some of the challenges of PPG and yield a more robust sensor technology used for vascular ageing.

An additional sensor technology that could be used in such multimodal applications is acoustical sensing.

The human body and organs generate acoustic signals, including sounds and vibrations, that propagate through the surrounding tissues and reach the body surface. These signals can be measured and monitored transcutaneously by acoustic sensors such as microphones, accelerometers, and gyroscopes. These signals provide information about the mechanical aspects of CV activity, and thus may contain diagnostic information. With the employment of advanced computational techniques (feature extraction, ML, wavelet transform, power spectral density, etc) these signals representing CV sounds can help into the classification of healthy and diseased groups. Also, acoustic pressure fluctuations have been recognized as the primary flow-induced source of the sound through arteries. These pressure fluctuations, and their correlation with highly turbulent shear stresses and vibration on the internal arterial wall can be further excited by blood flow turbulence due to medical conditions such as stenosis, vascular disease, and aneurysms (Cook *et al* 2022).

These signals may also provide complementary information to other monitoring methods. For example, while an ECG test, assesses the electrical activity of the heart, acoustic signals such as sounds and vibrations can provide a more in-depth understanding of the mechanical activity of the CVS. This complementary information can lead to the detection of early signs of CVDs.

In addition to the above, the emergence of US based photoacoustical (PA) sensors can provide an alternative means of developing the next generation CV monitoring technology (Zhao *et al* 2022). An US based PA imaging sensor delivers diffused light through tissue, where the photons are absorbed by dominant chromophores and converted to sound waves for acoustic imaging. Also, there has been strong evidence that PA can provide more accurate measurement of blood oxygenation levels compare to pure optical methods (such as widely used clinical pulse oximeter) which can be significantly affected by the skin tone (Mantri and Jokerst 2022). Such sensors not only will work in a complementary fashion with optical sensors but they will also contribute toward the real time imaging of vessels and circulation which will be an invaluable tool in the assessment of vascular disease.

Of course, the sky is the limit of what other sensors can be incorporate in this multimodal approach used for obtaining additional physiological information which can contribute further and more holistically in the assessment of vascular disease. These could include dry electrodes (designed and fabricated using flexible light-weight nano materials (e.g. carbon nanotube and/or graphene aerogel fibre electrodes)) for ECG measurements and temperature sensors, etc.

### Concluding remarks

In the current market, established diagnostic techniques have been widely used for the diagnosis and evaluation of vascular ageing. However, their limitations such as their high costs, bulky sizes, usability for early screening at GP level, and patient discomfort inhibit their application to certain patient groups and prevents their use outside of a hospital environment.

Advancements in wearables and sensing technology, coupled with the developments in computing, signal processing, and ML methods have enabled the long-term, real-time measurement and analysis of these signals even outside of healthcare facilities, which may result in the earlier diagnosis of diseases and the timely referral of the patients to their caregivers. Many of these devices are both practical and affordable, and enable monitoring of vulnerable populations from the comfort of their homes, while at the same time providing critical alerts for events requiring prompt medical attention or hospitalization.

The research community needs to grasp the opportunities provided by non-invasive sensor technologies and collectively focus their efforts, using a systematic approach, to innovate multimodal non-invasive sensor technologies for the early assessment of vascular ageing. Such transformative sensor solutions will enable the early diagnosis of vascular disease and prompt the timely treatments to both symptomatic and asymptomatic patients.

## Data Availability

The data that support the findings of this study are available upon reasonable request from the authors.
